# Home-based reach-to-grasp training for people after stroke: study protocol for a feasibility randomized controlled trial

**DOI:** 10.1186/1745-6215-14-109

**Published:** 2013-04-25

**Authors:** Ailie J Turton, Paul Cunningham, Emma Heron, Frederike van Wijck, Cath Sackley, Chris Rogers, Keith Wheatley, Sue Jowett, Steven L Wolf, Paulette van Vliet

**Affiliations:** 1 Department of Allied Health Professions, University of the West of England, Bristol, BS351NS, UK; 2 Glasgow Caledonian University, UK; 3 University of East Anglia, UK; 4 University of Bristol, UK; 5 University of Birmingham, UK; 6 Emory University, Atlanta, USA; 7 Newcastle University, Australia

**Keywords:** Stroke, Hand, Arm, Physical therapy, Occupational therapy, Rehabilitation, Home, Reach, Grasp, Task-specific training

## Abstract

**Background:**

This feasibility study is intended to assess the acceptability of home-based task-specific reach-to-grasp (RTG) training for people with stroke, and to gather data to inform recruitment, retention, and sample size for a definitive randomized controlled trial.

**Methods/design:**

This is to be a randomized controlled feasibility trial recruiting 50 individuals with upper-limb motor impairment after stroke. Participants will be recruited after discharge from hospital and up to 12 months post-stroke from hospital stroke services and community therapy-provider services. Participants will be assessed at baseline, and then electronically randomized and allocated to group by minimization, based on the time post-stroke and extent of upper-limb impairment. The intervention group will receive 14 training sessions, each 1 hour long, with a physiotherapist over 6 weeks and will be encouraged to practice independently for 1 hour/day to give a total of 56 hours of training time per participant. Participants allocated to the control group will receive arm therapy in accordance with usual care. Participants will be measured at 7 weeks post-randomization, and followed-up at 3 and 6 months post-randomization. Primary outcome measures for assessment of arm function are the Action Research Arm Test (ARAT) and Wolf Motor Function Test (WMFT). Secondary measures are the Motor Activity Log, Stroke Impact Scale, Carer Strain Index, and health and social care resource use. All assessments will be conducted by a trained assessor blinded to treatment allocation. Recruitment, adherence, withdrawals, adverse events (AEs), and completeness of data will be recorded and reported.

**Discussion:**

This study will determine the acceptability of the intervention, the characteristics of the population recruited, recruitment and retention rates, descriptive statistics of outcomes, and incidence of AEs. It will provide the information needed for planning a definitive trial to test home-based RTG training.

**Trial registration:**

ISRCTN: ISRCTN56716589

## Background

Stroke is a major cause of disability worldwide [1]. In England alone, approximately 110,000 people have a stroke each year, and many of these patients require continued rehabilitation after discharge from hospital [2]. However, the UK National Stroke Strategy published in 2007 stated that only 50% of patients with stroke receive rehabilitation that meets their needs in the first 6 months post-discharge and only 20% in the next 6 months [3]. This inconsistency of service provision was also identified by the Care Quality Commission [4], and has led to a UK-wide drive to increase the provision of community-based rehabilitation services for people with stroke [5,6].

Up to 85% of stroke survivors experience hemiparesis, which results in impaired movement of the arm [7]. Of these survivors, a large proportion (46 to 95%) is estimated to have continuing problems at 6 months after onset [8]. Loss of arm function has been shown to adversely affect quality of life and subjective wellbeing after stroke [9,10]. To improve outcomes, it has been recognized that research to determine the most effective interventions for promoting arm recovery should be a priority [11,12].

A Cochrane systematic review was recently conducted to address whether home-based upper-limb therapy programs are effective for recovery of function for people after stroke. The review found only four trials that met the inclusion criteria, and these provided insufficient evidence to answer the question [13]. Furthermore, none of the four tested interventions were suitable for participants with a severely affected limb. The review concluded that high-quality randomized controlled trials (RCTs) of therapy programs specifically targeting the upper limb are needed, and that these should be interventions in which participants are visited by health professionals at home.

Theoretically, home-based rehabilitation may be more beneficial than hospital-based or outpatient treatment by allowing repeated practice of occupationally embedded tasks in the person’s own environment, in accordance with the ‘specificity of learning’ principle [14]. This principle predicts that learning of a new skill is enhanced when conditions of practice match those of the task in real life. Practicing movement within tasks or actions has been termed ‘task-specific training’, and there is mounting theoretical evidence for its use in neurological rehabilitation of the upper limb, with massed practice of tasks as its central tenet [15]. The theory is supported by robust findings from research on skill acquisition, showing experience-dependent behavioral and neural changes in animals and humans [16-19]. Many motor functions are mediated through specific neural networks [20]. Therefore, if a specific action is to be improved, it would seem important to train and strengthen a network to regain that action rather than expecting training to generalize to many and varied upper-limb tasks [21-23]. The effectiveness of repetitive task training is partially supported by the findings of a Cochrane systematic review, in which the authors found clear benefits of repetitive task training for the function of the lower limb , but not for that of the upper limb [24]. However, the authors stated that the latter finding was ‘very tentative’ owing to the paucity of studies and methodological limitations, and recommended further research. Another review of stroke rehabilitation trials encompassing a wider spectrum of therapy interventions concluded that interventions focusing on high-intensity and repetitive task-specific practice showed the most promise for improving motor recovery [25]. There is a need for a well-designed trial to test the hypothesis that task-specific upper-limb training at home provides a significant improvement over the usual care currently provided for patients with arm impairment in the months after stroke.

Based on the theoretical framework underpinning task-specific training, it is reasonable to hypothesize that to be effective, training must target actions that are used frequently in a person’s everyday life. When stroke survivors have been asked about their goals for a therapy program for their affected arm, many of the goals involved RTG activities [26,27]. Reaching to grasp is an essential action used to perform everyday functions such as retrieving objects (for example, clothes, food, and drink), and is used more frequently than other arm actions such as gesturing, stabilizing objects, or providing support [28]. An effective intervention specifically targeting RTG tasks therefore has the potential to improve a stroke survivor’s ability to perform activities of daily living.

A fundamental problem for RTG task-specific training for stroke is that many patients do not have sufficient motor control to perform the whole action, therefore a definition of the intervention that will suit a wide range of impairments is needed. More severely impaired individuals could practice ‘part’ of an RTG task; therefore, for the purposes of this study, RTG task-specific training will be defined as a combination of whole-task training and part-practice. Part-practice will be undertaken through segmentation, that is, the RTG action will be broken down into components (for example, shoulder flexion, elbow extension, finger opening) that can be practiced separately or in combination, providing they are goal-directed.

Hubbard has recommended that to have the best chance of being effective, task-specific practice should be intensive [15]. Decisions about the frequency of the intervention therefore need to reflect the amount of therapy that can be reasonably delivered by community therapy services and how much patients can be asked to practice in the absence of a therapist. The Extremity Constraint Induced Therapy Evaluation (EXCITE) trial of constraint induced movement therapy for the upper limb, which resulted in significant improvement in outcome, delivered 60 hours of supervised therapy over 2 weeks to participants at 3 to 9 months post-stroke [29]. To approach the same number of hours with a schedule that is more likely to fit within the constraints of UK community rehabilitation, it was decided that a prescription of 14 visits of 1 hour each over the course of 6 weeks with up to 1 hour a day of self-monitored practice should be used. Combined with the therapist-supervised sessions, the total prescription will be 56 hours of practice.

Before designing a definitive trial, the acceptability of the intervention, the characteristics of the participating population, recruitment and retention rates, descriptive estimates of outcome, and incidence of adverse events (AEs) need to be determined. This paper outlines the objectives and design of a feasibility study, and describes how the data generated will be used to inform a Phase 3 trial.

### Study objectives

The aims of this feasibility trial are as follows.

1. To determine the characteristics of the sample entering the feasibility trial for its representation of the target population and to determine the most appropriate primary outcome measure for the sample, that is, the Action Research Arm Test (ARAT) or the Wolf Motor Function Test (WMFT).

2. To estimate identification and recruitment rates for the trial across multiple sites.

3. To determine the frequency and content of upper-limb treatment delivered to both the intervention and control groups.

4. To estimate the adherence of RTG group participants to the prescribed schedule.

5. To estimate the completeness of outcome data.

6. To calculate sample sizes for a subsequent definitive trial, based on measured changes in performance for both the control and intervention groups.

7. To collect and synthesize the views from participants in the intervention group to determine acceptability of the RTG therapy.

8. To determine the frequency of AEs in both groups.

9. To collect data on health and social care resource use to inform data-collection methods for an economic evaluation in the subsequent definitive trial.

## Methods

### Study design

**Figure 1:**
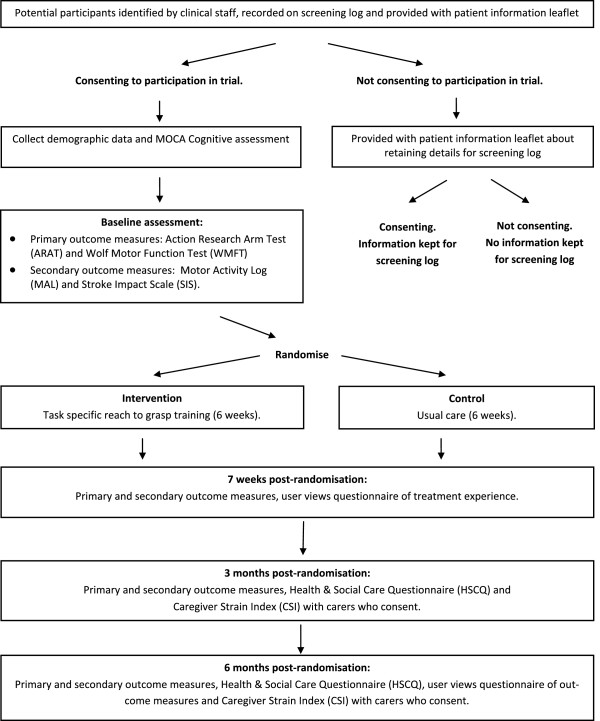
Design of the feasibility randomized controlled trial.

This study is a two-arm, multicenter, assessor-blinded feasibility RCT of task-specific RTG treatment. The control group will receive arm therapy in accordance with usual care. The trial design is summarized in Figure 1.

### Participant recruitment

Participants will be recruited from the stroke services of three National Health Service (NHS) Hospital Trusts and community therapy services, covering an area with a population of 918,300 [30]. This trial intends to recruit 50 participants over a period of 15 months. Eligibility criteria are designed to be as inclusive as possible to promote the applicability of the evidence to service delivery.

### Inclusion criteria

Patients will be eligible for the study if they: have been diagnosed as having had a stroke (the stroke does not have to be the first in their lifetime); are discharged home (that is, their permanent address, which may be a care home or sheltered accommodation); and have a residual deficit in upper-limb movement, defined as being unable to pick up a 6-mm ball bearing from the table top, between index finger and thumb, and place it on a shelf 370 mm above the table (pinch task from the ARAT [31]). This disability will be judged to be the consequence of the recent stroke.

To participate, patients must provide informed consent in accordance with the Mental Capacity Act, 2005 [32]. Non-English speakers will be included if the aid of an interpreter is available via the NHS. Patients with aphasia can be included, and the consent procedure will be facilitated using pictorial aphasia-friendly participant information booklets and communication other than spoken language (for example, gestures, strategic use of environmental cues).

### Exclusion criteria

Patients will not be eligible if they: have pre-stroke pathology of the stroke-affected upper limb preventing RTG, are unable to lift their hand off their lap when asked to place their hand behind their head (a Gross Motor task from the ARAT), have severe fixed contractures of elbow or wrist (that is, grade 4 on the modified Ashworth scale [33], or are more than 12 months post-stroke.

Potential participants will be identified by a clinician who will give the person an invitation letter and patient information sheet describing the study. The clinician will then ask the person’s permission to pass their contact details onto the research team. If the patient agrees, a member of the research team will arrange to visit the patient at home to undertake the consent procedure. Demographic data will be collected at the time of recruitment to allow description of the sample. These data will include age, gender, hand dominance, stroke classification, date of stroke, and co-morbidities. As a further descriptor, cognitive impairment will be assessed with the Montreal Cognitive Assessment [34].

Any patients who are eligible to take part in the trial but do not wish to participate will be asked if they consent to demographic information about them being kept on a screening log. This is to describe the sample with respect to the target population. These individuals will be given an information leaflet to help them to decide what, if any, information they consent to being recorded and kept by the research team. Retaining personal details of people declining participation is particularly important in this study, because people will be allowed to join the study up to 1 year post-stroke and they may encounter therapists in different services over the course of this time. Keeping the identifying information will help to ensure that the wishes of people who ask not to be approached about the trial again are honored. The screening log will allow data collection in accordance with the 2010 CONSORT statement [35], and help to ensure that we do not approach patients who have been recruited to other research studies that would be confounded by later participation in our trial.

Patients deemed eligible for the trial but who decline to participate will be asked if they consent to the following information being recorded on the centrally held screening log: initials, NHS number, date of birth, gender, date of stroke, reason for non-participation, and level of post-stroke upper-limb impairment at the time of approach. A quick and easy criterion for clinicians to use for logging impairment level was chosen. They will be asked to determine whether the individual is able or unable to carry out two tasks while seated with their back against a chair back and their affected hand in their lap: 1) ability to reach to touch their ipsilateral knee; and 2) ability to pick up a can of drink from a table.

These data will help to determine whether people with or without a basic level of hand function are declining to participate.

### Recruitment of carers

In addition to the people with stroke, their carers will be recruited for interview using a structured questionnaire: the Caregiver Strain Index (CSI [36]). In all cases, the trial participant must be in agreement for their carer to be approached. Information for the carer will be left with the participant. The carer may then indicate their willingness to be contacted by the research team by returning a tear-off slip in a stamped addressed envelope. The person with stroke may participate even if the carer declines to be interviewed.

### Outcome measurement

Baseline assessments will be conducted within 1 week of recruitment and before randomization. Outcome assessment will be performed at 7 weeks post-randomization, and follow-up assessments at 3 and 6 months after randomization. The assessments will be carried out in the participant’s home by an assessor who is blinded to group allocation and who is trained in the assessment procedures. All of the following assessments will be undertaken at all four time points: ARAT, WMFT, Motor Activity Log (MAL), and Stroke Impact Scale (SIS). The health and social care questionnaire and the CSI will be undertaken only at the follow-up assessments.

One of the objectives of this feasibility trial is to determine which functional measure should be the primary outcome measure in a subsequent definitive trial. Two measures of arm function will be tested to see which will be most suitable for the sample given the level of functioning of the recruited sample and participants’ preferences: the ARAT [31] and the WMFT [37]. The ARAT consists of 19 items focusing on reaching and grasping objects of different shapes and sizes, and lifting them onto a shelf, and also has a section for rating gross arm movements. The ARAT is best suited to people who have hand function; those who are unable to grasp and release will only be able to score points in the Gross Motor section. Item scoring is ordinal (0, 1, 2, or 3), with higher values indicating better performance. The test has high inter-rater reliability (intra-class coefficient (ICC) = 0.98) and test–retest reliability (ICC = 0.99) and good validity, and, in those with hand function, is sensitive to therapy-related changes after stroke [38,39]. A standardized protocol for the ARAT will be followed [40], for which high reliability scores have been established in a multi-center study [41]. The WMFT assesses both isolated arm movements and performance of functional tasks, and so will suit low-functioning participants better. In the WMFT, 17 actions are measured according to time taken to complete them and a quality rating of the use of the affected hand in attempting the task (graded 0 to 5). The WMFT has high inter-rater reliability for time scores (ICC = 0.97) [37], for quality of performance score (ICC = 0.88), and test–retest reliability (ICC = 0.90 for performance time and ICC = 0.95 for quality ratings) [42]. To help determine the most appropriate primary outcome measure for a definitive trial, participants will be asked their opinions of the outcome measures, and which they consider best reflects meaningful change. This will be performed using a structured questionnaire administered by the assessor at the time of the final follow-up. Assessors will keep a log of aspects of administration of the tests that threaten their reliability.

There will be three secondary outcome measures.

1) The MAL. Participants are asked to rate on a six-point scale the level of use and quality of their arm movement for performing 28 everyday tasks. The MAL is a reliable and valid measure for people up to 12 months post-stroke [43].

2) The SIS. This is an interviewer-administered measure, used to obtain self-report ratings on a five-point scale of difficulties or restrictions in eight domains: limb strength, memory and thinking, mood and emotions, communication, activities of daily living, indoor and outdoor mobility, hand function, and social participation. Participants are also asked to rate their perception of percentage of their recovery. It has excellent internal consistency (α = 0.83 to 0.90) and adequate to excellent test–retest reliability (ICC = 0.7 to 0.92 with the exception of the Emotion domain (ICC = 0.57)) [44].

3) Caregiver burden will be assessed using the CSI, a 13-question tool that measures strain related to care provision in the following domains: employment, financial, physical, social, and time. Participants are asked to agree or disagree with the statements. The scale has established internal consistency (Cronbach’s α = 0.86) and correlates with caregivers’ subjective perceptions of the care giving relationship and the physical and emotional health of the caregiver [36].

In addition, an assessment of health and social care costs will be undertaken. Patients will be asked to complete a health and social care questionnaire at 3 and 6 months to estimate all condition-related primary and secondary care costs and use of any social care services. The cost of delivering task-specific RTG training and usual care will also be estimated, and details such as actual time spent by the therapist with the patient, travel costs, frequency of visits, and equipment used will be recorded. This is in preparation for economic analysis in a future Phase 3 trial.

### Data management

Data will be collected and retained in accordance with the UK Data Protection Act 1998 [45], and managed in accordance with the trial-specific standard operating procedure for data management.

With their consent, patient details will be passed between NHS services and the research team by telephone or in person (that is, not electronically via email or text message). All patients who provide consent will be allocated a unique study ID. The information linking each participant’s study ID to their personal details will be kept securely at the University of the West of England (UWE). All other patient-related paper records will be anonymized and stored separately from the personal information.

The electronic database for the trial will be stored on the secure servers of the University of Bristol with password-controlled access provided for the UWE research team by the Bristol Clinical Trials and Evaluation Unit (CTEU). Single data entry with extensive in-built validity checks will be used to reduce the risk of transcription errors. The study database will include prompts for missing data, and warnings to alert staff when values are entered that are outside of the expected range or are inconsistent with other data already entered, or if the type of value entered is incorrect (for example, a numeric value entered rather than text).

### Randomization

Participants will be randomly allocated to the intervention or to the usual care groups in a 1:1 ratio after baseline assessment. Randomization will occur via an independent internet-based service, and the procedure will use minimization to balance time since stroke, severity of upper-limb impairment, and group numbers between the two arms. Time since stroke will be categorized as less than or equal to 3 calendar months post-stroke, or more than 3 calendar months post-stroke. This 3-month cut off was chosen to balance between groups the number of participants within 3 months of stroke, who might be recovering at a faster rate [46]. Baseline severity will be categorized using scores on the ARAT in three subgroups defined by Morris *et al*. [47] (group 1: score 0 to 3; group 2 score 4 to 28; group 3 score 29 to 56).

The data for the randomization procedure will be entered by the trial manager. He will have no influence over the allocation to group because the randomization procedure is independent, and he will not be involved in measuring outcome. Post-randomization, the trial manager will contact the patient and any NHS services involved in the patient’s care to inform them of the treatment allocation.

### Blinding

All research personnel responsible for assessing outcome will be blinded to the participant’s group. Owing to the nature of this behavioral intervention, those involved in the delivery of the treatment will not be masked and nor will the participant. Measures will be taken to minimize the potential for the assessor to become unblinded. These will follow the guidelines recommended by Siemonsma and Walker [48], and include the assessor and research physiotherapist having separate offices, telephones, and document-storage facilities, and avoiding discussion about participants in front of the assessor. The trial manager will make appointments for the assessment by telephone, and will use this opportunity to remind participants not to reveal details of any treatment they have received to the assessor. Incidents of unblinding will be recorded immediately after the assessment in a note to file in the case report form. With reference to these notes, the assessor will be asked to fill in a questionnaire at the end of their involvement in the study to identify whether they know or have guessed each participant’s group. These data will be compared with the actual group allocation to determine the success of the blinding, as recommended by Minns Lowe *et al*. [49].

### Intervention group

To determine the content of the RTG training, the therapist will initially assess the patient’s attempt of the whole task, and analyze which of its components need to be prioritized for practice. For example, a patient with weak shoulder flexion may practice reaching by sliding the hand forward over a non-resistant surface to touch an object or grasp it. The training tasks should be challenging and progressively adapted by chaining; that is by practicing first one part, then practicing that part along with the next [50]. Patients showing sufficient improvement may progress in this way to mastery of the whole task.

To optimally standardize the intervention, a detailed description of the underlying principles of the task-specific RTG intervention has been developed using the ‘essential components’ of the RTG movement as a structure [51] (see Additional file 1). An exercise manual containing 122 exercises has been produced for therapists to follow (see Additional file 2). Each exercise involves interacting with objects and the environment, and is set up to promote practice with joint range and speed of the movement, that is, close to those characteristics of the task when performed as a whole RTG action. The therapist can individualize exercises for patients by advising on or providing environmental adaptations; for example, by choosing meaningful objects of different sizes, weight, and shape, or by recommending a starting position for the patient. Little or no manual guidance will be used. Over the course of the feasibility study, further exercises may be incorporated as new ones are determined by working with the study participants.

Starting in the week after baseline assessment, participants allocated to the RTG training will receive 14 visits, each lasting 1 hour, from a research therapist over a period of 6 weeks. This will replace any usual care training for the upper limb. The frequency of the research therapist’s visits will be three times a week for the first 3 weeks, twice a week for each of the next 2 weeks, then once in the final week. The frequency of visits is tapered with the aim of increasing self-efficacy in practice. The intensity of practice within each 1-hour session will be dependent on individual participant’s capabilities, but high numbers of repetitions will be encouraged, with the aim of delivering between 100 and 300 repetitions within each 1-hour session, in accordance with the findings of a recent feasibility study into frequency of task-specific training [52]. To facilitate such a high number of repetitions, clinical experience has indicated that it is appropriate to limit the number of exercises prescribed within each session to a maximum of six, and to a maximum of four for participants to continue to practice independently. The research therapist will keep a log of which exercises from the treatment manual are performed, and of the number of repetitions of each exercise.

From the beginning of the intervention phase, the participants will be instructed to practice in the absence of the therapist for 1 hour/day. They will be asked to record on a log sheet the number of repetitions of each task that they complete in their independent practice. There is no method in place in this feasibility study to check on the fidelity of these records, as this would have required resources beyond those available for this study. The therapist will stress to the participant that an accurate record is important to enable us to determine the feasibility of the amount of practice prescribed. They will be requested to record reasons for non-adherence to the practice schedule. This information will be collected at the end of the intervention period.

To help participants understand the importance of practice for recovery and to promote compliance with the RTG task-specific training, a booklet about recovery from stroke will be provided (see Additional file 3). The booklet describes in simple terms the mechanisms of stroke and the potential for recovery by ‘rewiring’ in the brain through practice, and also provides contact details of local resources and services for people with stroke. A recent Cochrane review on information provision for patients with stroke and their caregivers showed that providing information improves patient and carer knowledge of stroke, improves aspects of patient satisfaction, and reduces patient depression scores [53].

Participants receiving the RTG training will be asked their opinions of the intervention using a structured questionnaire administered face to face by the research physiotherapist after their final intervention visit. With limited personnel to carry out these interviews, the risk of bias inherent in using the research therapist for these interviews was considered preferable to requiring the assessor to do these interviews, as this could cause unblinding. Data from the interviews will inform the acceptability of the intervention content and frequency.

### Control group

The control group will receive arm therapy in accordance with usual care, which will be delivered by NHS service therapists. In usual care, the frequency and content of physiotherapy or occupational therapy for improving upper-limb function is variable. From consultation with local therapy services, we found that provision of therapy varies in intensity, depending on the individual’s care pathway and the range of community services available. For example, after discharge from early supported discharge services, patients with care needs are usually referred to community intermediate care or rehabilitation teams. These teams typically provide a service for 6 weeks, and patients may receive approximately two visits per week from a qualified therapist, with 30 minutes of the visit focused on the arm or hand. There may be supplementary visits from support workers. Other patients may be seen by a community therapist once a fortnight with no supplementary visits from support staff, and some patients may not receive any therapy at all. It is unusual to receive arm therapy beyond 6 months after discharge from hospital. One of the aims of this feasibility study is to determine the frequency and content of treatment delivered in both arms of the trial. Usual care will be captured on recording sheets that will be completed by NHS service physiotherapists and occupational therapists. They will be asked to record the content, duration, and frequency of upper-limb treatment, including the number of repetitions of any functional task training on treatment recording sheets developed by Donaldson *et al*. [54].

Participants allocated to the control group will be provided with a booklet about recovery after stroke, which is the same as the booklet which will be given to the intervention group, with the exception that, instead of information about the RTG training, it will provide details of the frequency of assessment visits for the study (see Additional file 4).

Participants in the control group who do not receive any therapy aimed at improving upper-limb function during their participation will receive a single visit from the research physiotherapist after their final assessment. The physiotherapist will discuss the patient’s functional limitations and goals with them and, after assessing their movement and function, will provide specific exercises and advice. If indicated, the physiotherapist is able to refer to appropriate local therapy services.

### Adverse events

Pain in the shoulder, upper arm and hand and oedema in the hand are common sequelae of hemiparesis, and are therefore expected AEs in the study. In addition, falls and equipment failure leading to injury requiring a visit to hospital or general practitioner will be treated as expected AEs. It is possible that increased use of the upper limb may lead to an increase in general activity in the home or community, and could result in increased likelihood of falling. The ARAT and WMFT assessments require that equipment is taken into the participant’s home. Although every effort will be made to minimize risk, accidents may happen when using any equipment. Participants will be specifically asked at each of the assessment visits if they have experienced any of the expected AEs.

All staff involved in contact with participants will receive training on identification and reporting of AEs. Any serious AEs that occur will be reported to the trial sponsor, and if the chief investigator (CI) considers any of these to be related to the RTG intervention, these AEs will be reported immediately to the research ethics committee that granted approval for the trial. Incidence of AEs will be collected by the trial manager and reported quarterly to the trial steering group.

### Data analysis

As this is a feasibility study to test the levels of recruitment and retention of participants, and completeness of data that can be expected within a definitive multicenter trial, there will be no formal statistical testing, and results will instead be summarized using descriptive statistics.

Analysis of the centrally held screening log data will lead to a description of the characteristics of the recruited sample against the target population, and the percentage of refusals at the recruitment stage. Recruitment rates per month for each site will be determined. The number of withdrawals and reasons for withdrawal from the study will be assessed.

Compliance with the RTG intervention will be assessed by the number and duration of physiotherapy visits and the proportion of participants completing the 6-week intervention. The frequency of use of the exercises, number of repetitions, and duration of practice of each exercise, both within the physiotherapy sessions, and the independent practice will be summarized and compared with the intended exercise frequency and content. The frequency and content of the treatment received by each group will be summarized, as will the incidence of expected AEs and serious AEs. Responses to the participant questionnaire about the RTG training will be synthesized and examined to determine if any changes should be made to the intervention.

Completeness of outcome data will be determined, and data for each of the endpoints will be summarized; the mean differences between the arms, with their confidence intervals, will be presented. Assessment of the sample scores on the outcome measures, combined with a summary of participant preference for ARAT and WMFT, will be carried out to determine which arm function test should be used as the primary outcome measure for a phase III trial. Sample sizes for a subsequent phase III trial will be calculated, based on observed changes from baseline in performance, for both the control and intervention groups.

### Trial monitoring and management

The trial manager will manage the day-to-day running of the study. Before the study commences, training sessions for clinical staff involved at each site will be organized by the UWE research team. These sessions will ensure that personnel involved fully understand the research protocol, delegation log, and standard operating procedures for the study.

The UWE research team will meet regularly (ideally fortnightly) throughout the trial. The trial steering group, CI and trial manager will have a teleconference every 4 months to discuss progress, including recruitment, withdrawals, treatment compliance, and AEs. The steering group will provide advice on amendments to protocol if necessary.

The Bristol CTEU, a clinical trials unit registered with the UK Clinical Research Collaboration, will specify the randomization scheme, develop and maintain the study database, and carry out trial analyses in collaboration with the investigators. An independent data monitoring and safety committee will not be convened for this pilot study, as there is very low risk of intervention-related AEs and a short recruitment, intervention, and follow-up period.

### Ethical considerations

The study has been approved by the National Research Ethics Service, South West Southmead Research Ethics Committee (REC) (ref number: 10/H0102/83) and the sponsor’s REC at the Faculty of Health and Life Sciences, UWE, Bristol. The study has been approved by the Research and Development (R&D) departments responsible for each site. This study will be conducted in accordance with the principles of the International Conference for Harmonisation of Good Clinical Practice (ICH GCP) guidelines [55] and the Research Governance Framework for Health and Social Care [56]. Information about possible benefits and risks of participation will be described in the patient information sheets. Any amendments to the trial documents must be approved by the sponsor before submission to the REC and R&D departments.

The study will be monitored and audited in accordance with the sponsor’s policy, which is consistent with the Research Governance Framework [56]. All study-related documents will be made available on request for monitoring and audit by the sponsor or funder and the relevant REC. This is a UWE-sponsored research study, working in collaboration with NHS trusts. The University's Clinical Trials insurance cover provides either legal liability cover or non-negligent/no-fault compensation cover.

## Discussion

Recovery of the upper limb after stroke may take many months, particularly in those more severely affected [46]. Effective home-based therapy programs are needed to help improve hand and arm function in the stroke population. An important part of developing and evaluating interventions is to describe them well. There is acknowledged ambiguity in definitions of the content of home-based therapy programs [13]. This paper, in conjunction with the publication of the exercise manual (see Additional file 1; see Additional file 2) and participant booklets (see Additional file 3; see Additional file 4) aims to provide a detailed description of the development and planned implementation of the RTG intervention.

At present, few studies have evaluated home-based therapy for the upper limb [13], and designing a trial to be conducted in a community setting can present new challenges. Community rehabilitation and therapy services within the UK are variable in their provision and fragmented in their organization [57], and similar difficulties have been reported internationally [58,59]. The stroke pathway passes through multiple services and organizations, and varies between catchment areas, so the number of clinical personnel involved is considerable (about 100 for the area used in this feasibility study), with high staff turnover. Therefore, putting into place procedures that are sufficiently flexible for identifying and recruiting potential participants in hospital stroke services and in the community is important. Devising viable procedures for recording the demographic information of people who have been approached but who decline participation is also essential in order to avoid duplication and to enable assessment of the characteristics of the recruited sample with respect to the target population.

Careful planning to overcome the challenges of community-based stroke-rehabilitation research is essential to prepare for a trial of adequate size and quality to assess a home-based upper-limb therapy program. In previous studies of upper-limb repetitive task training, significant methodological limitations have been acknowledged, for example, refusals to recruitment varied from 4 to 73% of potential participants [24]. Before designing and conducting an adequately powered, high-quality RCT of home-based upper-limb repetitive task training, a feasibility study should be completed to provide the information necessary. This paper has defined the aims and objectives of a feasibility study of home-based RTG training for people after stroke, and provided a detailed description of the intervention and study design.

### Trial status

The first participant was enrolled onto the trial in December 2011. Recruitment is ongoing, with 46 participants enrolled to date.

## Abbreviations

ARAT: Action research arm test; CI: Chief investigator; CTEU: Clinical trials and evaluation unit; MAL: Motor activity log; RCT: Randomized controlled trial; REC: Research ethics committee; RTG: Reach-to-grasp; SIS: Stroke impact scale; UWE: University of the West of England; WMFT: Wolf motor function test.

## Competing interests

The authors declare that they have no competing interests.

## Authors’ contributions

AT, FvW, CS, KW, SJ, SW, PvV are grant holders. Additional intellectual contributions were made by CR, PC and EH. All authors contributed to the development and writing of the protocol to its final version. All authors have been involved in the drafting and revision of this manuscript, and have given approval of the final manuscript.

## Supplementary Material

Additional file 1 Description of intervention. Click here for file

Additional file 2 Reach-to-grasp exercise manual. Click here for file

Additional file 3 **Intervention group *Recovery after Stroke *** **booklet.** Click here for file

Additional file 4 **Control group *Recovery after Stroke *** **booklet.** Click here for file
