# Supplementary material for: Home-based reach-to-grasp training for people after stroke: study protocol for a feasibility randomized controlled trial
Source: Trials. 2013 Apr 25;14:109. doi: 10.1186/1745-6215-14-109 (PMC3675391; doi:10.1186/1745-6215-14-109)
Supplement: Additional file 1 — Description of intervention. [file 1745-6215-14-109-S1.pdf]

University of the
West of England
BRISTOL

Therapy Manual

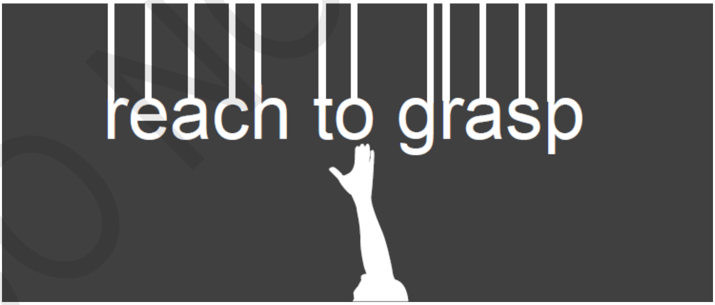

DO NOT PRINT

Contents

1. Shoulder	a. Protraction: 1 DoF	1
	b. Flexion: 1 DoF	1-6
	c. Extension: 1 DoF	1-2
	d. Abduction: 1 DoF	1-4
	e. External rotation: 1 DoF	1-14
2. Shoulder and elbow	a. Combined shoulder flexion with elbow flexion for transport: 2+ DoF	1-4
	b. Combined shoulder flexion with elbow extension for transport: 2+ DoF	1-3
	c. Combined shoulder flexion and external rotation with elbow flexion for transport: 3+ DoF	1-5
3. Elbow	a. Extension: 1 DoF	1
4. Forearm	a. Pronation/supination - forearm supported: 1 DoF	1-3
	b. Pronation/supination - forearm unsupported: 1+ DoF	1-5
	c. Pronation/supination with elbow flexion for transport: 2+ DoF	1-2

DO NOT PRINT

Contents

5. Wrist	a. Extension: 1 DoF	1-7
	b. Radial deviation: 1+ DoF	1-3
	c. Extension and radial deviation maintaining static forearm position: 2 DoF	1
	d. Extension, radial deviation and elbow movement: 3+ DoF	1-6
6. Hand opening	a. Finger extension: 1+ DoF	1-6
	b. Thumb abduction: 1 DoF	1-6
	c. Finger extension and thumb abduction maintaining static forearm position: 2+ DoF	1-4
	d. Finger extension, thumb abduction and elbow movement: 3+ DoF	1-3
7. Pinch grasp	a. Pinch grip between index finger and thumb only: 2 DoF	1
	b. Pinch grip with transport maintaining static forearm position: 2+ DoF	1-6
	c. Pinch grip with transport including elbow movement: 3+ DoF	1-11
8. Intrinsics	a. Cupping of hand: 3+ DoF	1
	b. Conjunct rotation: 3+ DoF	1-2
	c. Fine motor tasks: 3+ DoF	1-6
9. Task	a. Whole task practice: 3+ DoF	1-9

DO NOT PRINT

Assessment

Initial Analysis of Reach-to-Grasp (RtG)

To decide which components to practice and the mode of practice for the whole task, the therapist performs an initial analysis of the movement, by comparing the biomechanical features of the patient's performance of reach-to-grasp to a healthy model of reach-to-grasp performance. The therapist uses the following checklist to check individual components of reach-to-grasp, and aspects of whole movement performance against their knowledge of the healthy model, taking note of whether these components are present or absent, and the amplitude of each component's contribution to reach-to-grasp.

RtG components checklist:

- Shoulder flexion
- Scapular protraction and lateral rotation
- Shoulder external rotation
- Elbow extension
- Forearm supination
- Wrist extension and radial deviation
- Thumb abduction and opposition
- Finger extension with interphalangeal joints in some flexion

Some reaching tasks may also involve:

- Cupping of hand
- Conjunct rotation

DO NOT PRINT

RtG whole movement:

- Speed
- Accuracy
- Smoothness
- Coordination

The therapist decides which of these components/whole movement aspects requires training most urgently. Up to 3 components/whole movement aspects may be chosen per training session.

Ongoing Analysis

The therapist repeats the analysis briefly at the beginning of each one to one training session and adjusts their choice of aspects to train accordingly.

The therapist should observe for compensator y movements and aim to minimise by making the patient aware or through alteration of the task or environment .

DO NOT PRINT

Treatment

Choosing the Exercise

For each of the components/whole movement aspects, the exercise manual contains several exercises. After identifying the aspects to be trained, the therapist chooses from the exercises available in the manual. The exercises are ordered proximally to distally based on the RtG components checklist. Within each section the exercises are categorised according to the main degrees of freedom involved in the exercise. Where an exercise is focussed on specific degrees of freedom but it is acknowledged that other degrees of freedom may be required to complete a movement a + plus sign has been added.

E g. the task: ‘Pick up small object then replace’ is labelled as ‘Pinch grip - static forearm: 2+ DoF’ as the main movements being trained are finger extension and thumb abduction but it is acknowledged that this movement also involves, finger flexion, thumb abduction and radial deviation.

In the context of motor control, a degree of freedom (DOF) can be described as the smallest number of coordinates, required to describe the state of a system. For example, the proximal interphalangeal (PIP) joint of the 4th digit has one degree of freedom, as it has one axis of movement, around which it is “free” to move (i.e. flexion/ extension). Therefore just one coordinate (e.g. 45° degrees flexion from the anatomical position) is sufficient to describe the position of this system. The knee joint has two degrees of freedom, as it has two axes, (being a pivotal hinge joint, it permits flexion and extension as well as a slight medial and lateral rotation), while the hip joint has three – and so on.

DO NOT PRINT

The particular exercise is chosen by the therapist bearing in mind the following:

- *Amount of weakness present in the target muscle groups*
- *Amount of compensatory muscle activity when performing the exercise*
- *Information processing capacity of the patient*

The therapist can focus any of the exercises on a specific range of movement dependent on individual patient's abilities

Some of the exercises can be for patients to do themselves, and others could be to do with therapist. The therapist prescribes appropriate exercises from the manual for home practice .

Note: For any exercises involving shoulder flexion or abduction above 60 degrees, patients must have at least neutral external rotation range to reduce the risk of shoulder impingement.

Adverse events

Participants in the study should be advised to report any adverse events to the therapist. These may include upper arm pain, hand pain, oedema or falls. The therapist should record these events and manage appropriately, involving the participant's GP or other services as required. Other adverse events are possible and participants should be encouraged to report any concerns they have to the therapist.

DO NOT PRINT

Variation of Exercises

The exercises provided in the manual can be varied in the following ways, at the discretion of the therapist, to suit the needs of the patient and to encourage maximal progression in performance of the movement:

- *Amplitude of the movement required (e.g. the distance to be reached by extension of elbow and flexion of the shoulder*
- *Direction of movement (e.g. shoulder flexion in a medial direction with adduction, or in a lateral direction with abduction)*
- *Load (e.g. weight object being carried)*
- *Size/dimensions of object*
- *Height of the target (e.g. shoulder flexion - could start with enough to reach an object on a small stool on the floor and progress to a higher shelf)*
- *Degrees of freedom being used (e.g. shoulder flexion with or without elbow extension or external rotation included) Type of muscle contraction required – concentric, eccentric, isometric*
- *Speed of muscle contraction (utilising fast or slow twitch muscle fibres)*
- *Joint range (e.g. practice is stronger or weaker part of joint range)*
- *Amount of friction to overcome (e.g. sliding arm along a table, could use powder, slippery material, or skate underneath arm to lessen friction, to make movement easier)*
- *By using both arms together if appropriate to the task*
- *By using physical (e.g. seatbelt) or environmental restraint (object to discourage particular movements)*

DO NOT PRINT

Information Processing Demands

In addition to the variations above, task complexity can also be increased by requiring greater information processing. This can be achieved by adding an extra task, called ‘dual tasking’, for example talking while reaching, doing one thing with hand and another with the other, or standing up to reach for something. A subsequent task could also be added so the movement is performed in a natural sequence such as grasping and lifting a comb off a table and combing the hair, picking up a pen and writing or picking up a jug and pouring.

Information processing demands can also be increased by increasing speed and accuracy demands, such as moving the arm within a narrow defined pathway at progressively increasing speed.

Position of Patient

For all exercises, assume the patient is in sitting unless otherwise stated. Other suitable positions for exercises include standing or side-lying. Sitting or standing are more usual for reach-to-grasp movements in everyday life, but it may be necessary for the patient to be in side or supine lying if their muscles are too weak to perform the movement in sitting or standing.

After completing an exercise the patient can actively return their affected arm to the starting position or they can use their less affected arm to assist.

DO NOT PRINT

Use of Objects in the Hand

An everyday object (such as a cup, remote control, or ruler) can be held in the hand whilst practising the exercise, or alternatively, an object might be placed on a surface and the hand can reach for, or interact with it.

It is preferable to be interacting in some way with an object. The patient can place the object into their hand using their less affected arm if necessary, or the therapist can tape the object into the patient's hand with suitable tape such as micropore.

Number of Repetitions

The number of repetitions of each exercise per treatment session will be recorded by the therapist. The patient is to be asked to complete a log of the number of repetitions they do of each exercise in their self-monitored practice sessions. Recent studies have shown that the number of repetitions of upper extremity functional task training people with stroke are able to perform in each one hour session can vary between 32 to 300. The number of repetitions should be maximised within the training session, to promote motor learning, according to the capability of the patient.

DO NOT PRINT

DO NOT PRINT

This trial is funded by the

Stroke Association

And supported by the

Stroke Research Network
