# Supplementary material for: Home-based reach-to-grasp training for people after stroke: study protocol for a feasibility randomized controlled trial
Source: Trials. 2013 Apr 25;14:109. doi: 10.1186/1745-6215-14-109 (PMC3675391; doi:10.1186/1745-6215-14-109)
Supplement: Additional file 2 — Reach-to-grasp exercise manual. [file 1745-6215-14-109-S2.pdf]

1.a.1

Reach up to touch light cord

- Lay on your back
- Start with your elbow straight and hand pointing up to the ceiling
- Try to stretch up towards the ceiling
- Your carer / therapist may need to assist you to complete the movement

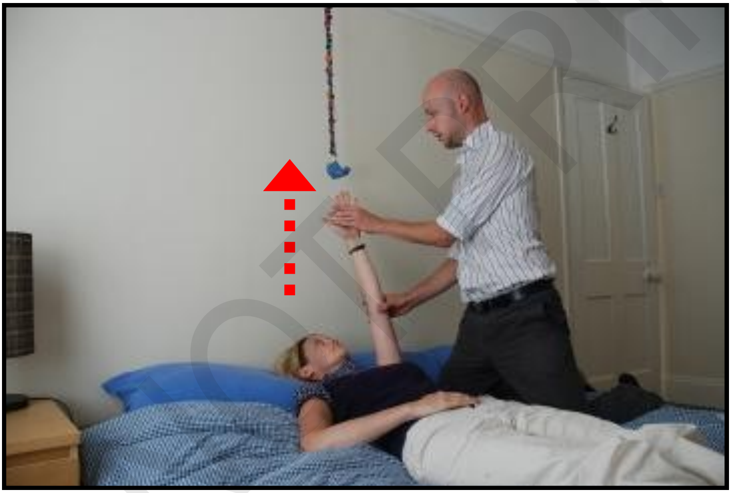

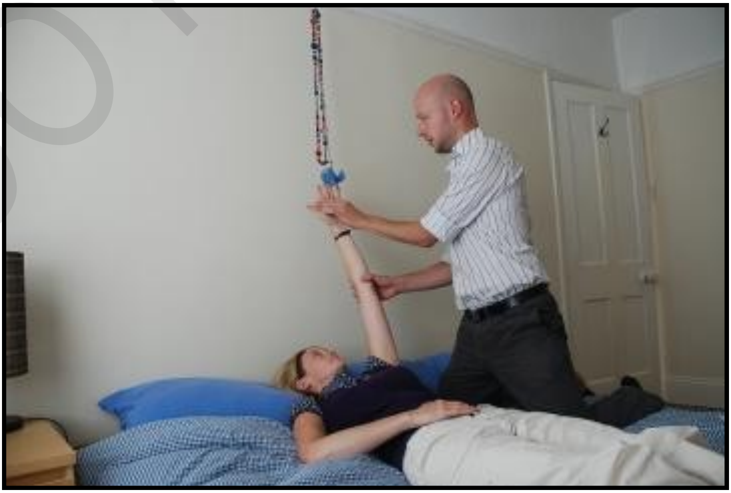

DO NOT PRINT

Reach for light cord

- Lay on your back
- Start with arm resting on bed
- Try to reach to touch light cord
- Your carer / therapist may need to assist you to complete the movement

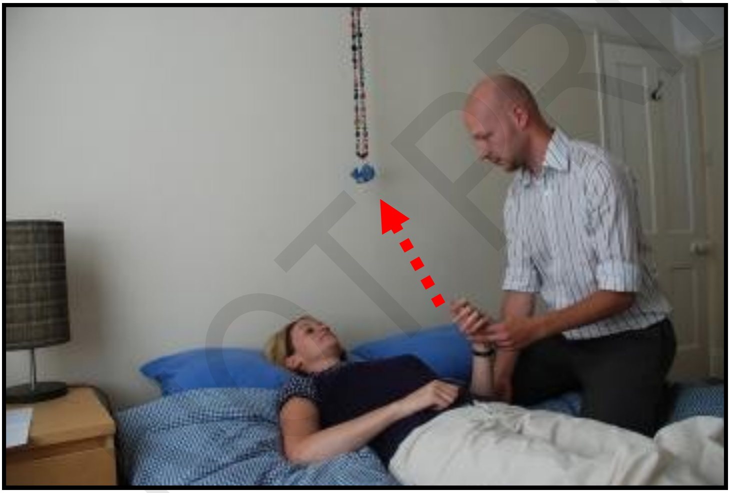

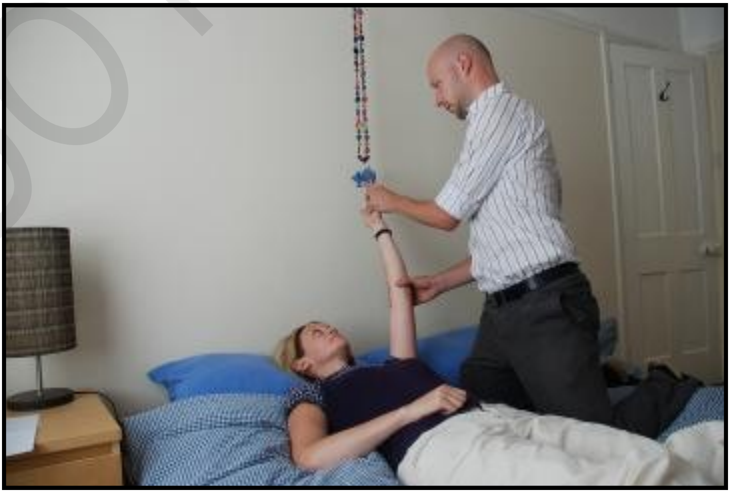

DO NOT PRINT

Reach to touch head board or wall

- Lay on your back
- Start with arm resting on bed
- Try to reach to touch the headboard or wall above you
- Your carer / therapist may need to assist you to complete the movement

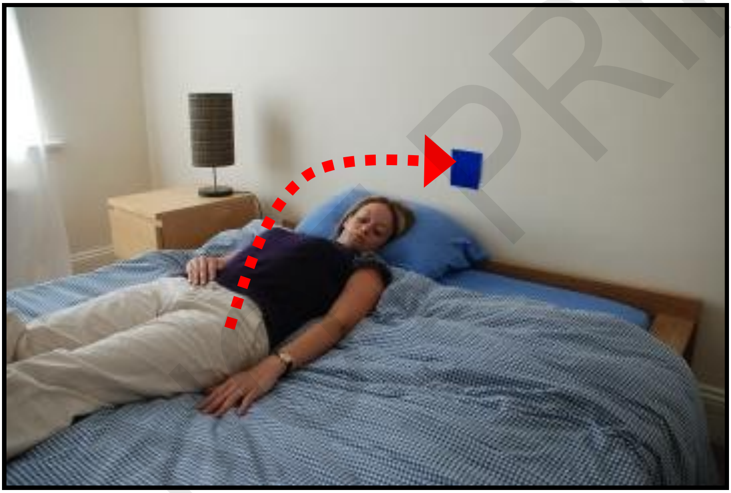

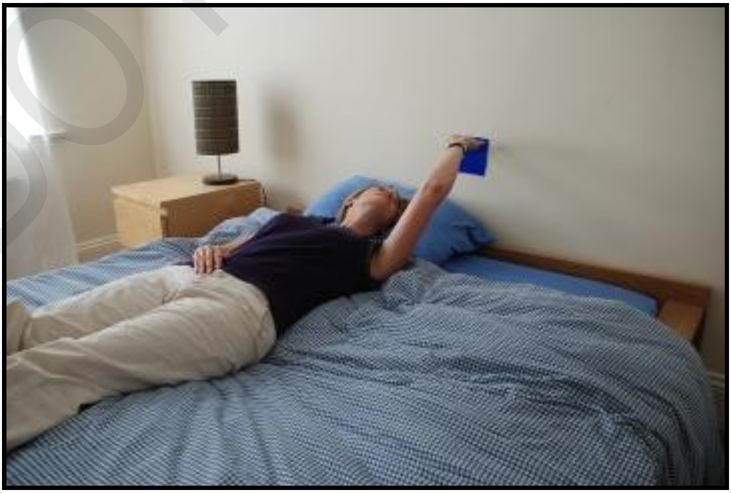

DO NOT PRINT

1.b.3

Reach to touch alarm clock

- Lay on your side with your weaker arm upper most and supported on a pillow
- Reach out to the side to try to touch your alarm clock / bed side table / etc.
- Your carer / therapist may need to assist you to complete the movement

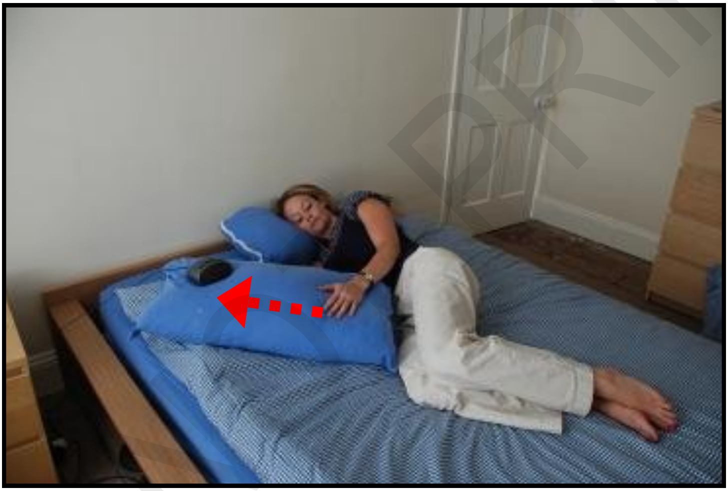

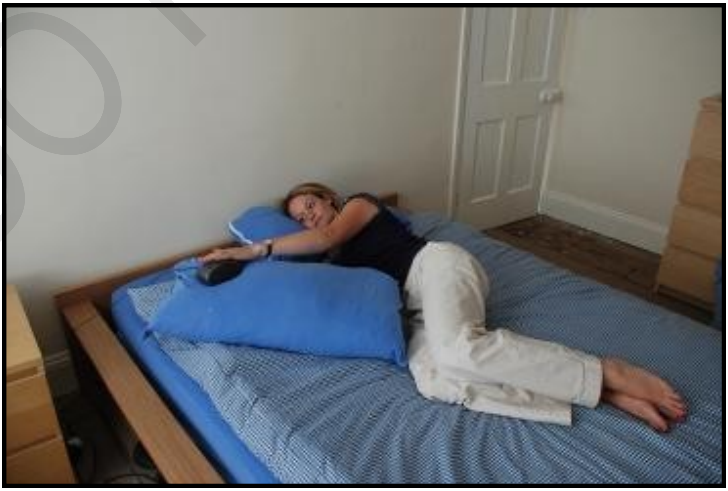

DO NOT PRINT

1.b.4

Moving straight arm between 2 'target's

- Lay down on your back with your arm raised up vertically
- Your carer / therapist can provide two 'target's with their hands.
- Move your hand from one 'target' to another by moving your arm back and forth.
- Ensure that your elbow is kept straight.

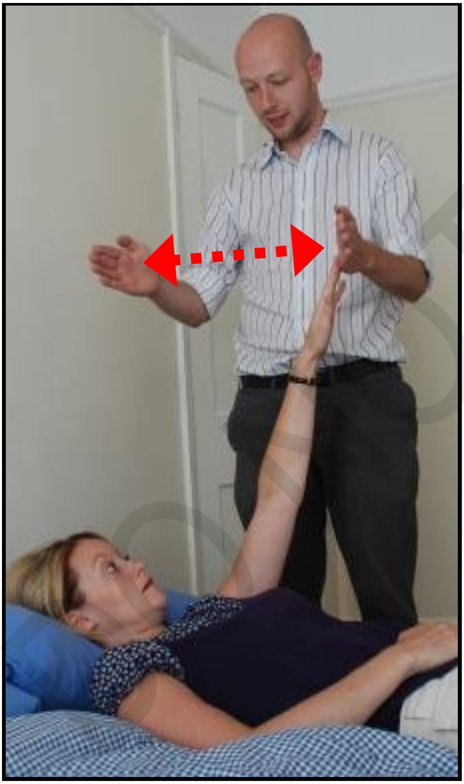

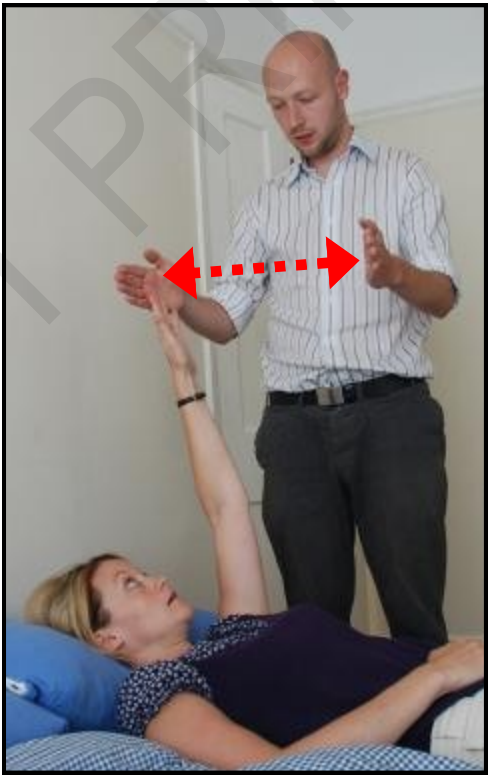

DO NOT PRINT

1.b.5

Touching hand to ‘target’

- Sit on a kitchen chair with your hand hanging freely at your side
- Try to swing your arm forwards to touch something placed just behind your knee, for example, a wooden spoon handle.
- Keep your back against the chair and try to keep your elbow straight

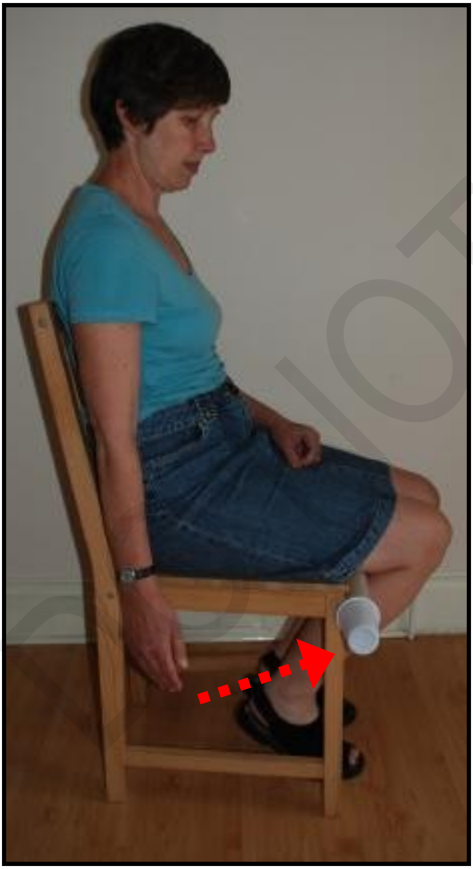

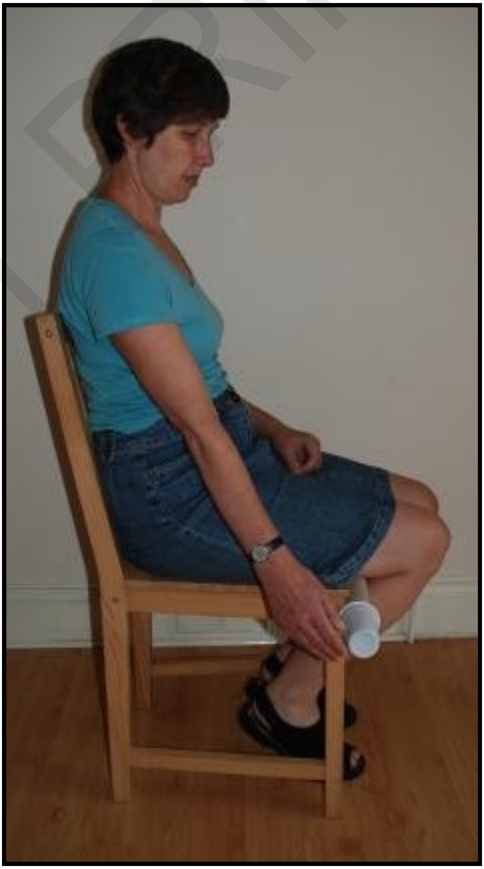

DO NOT PRINT

Lifting up an object

- Sit at a table with a long vertical tube in front of you with a slightly wider but shorter tube over the top of the other tube - your therapist will show you how to set this up
- Lift the shorter tube so it slides up the longer tube
- During the exercise, you should stay sat up straight, your shoulders should stay level and your elbow straight
- Lower your hand back down to the table **slowly**

DO NOT PRINT

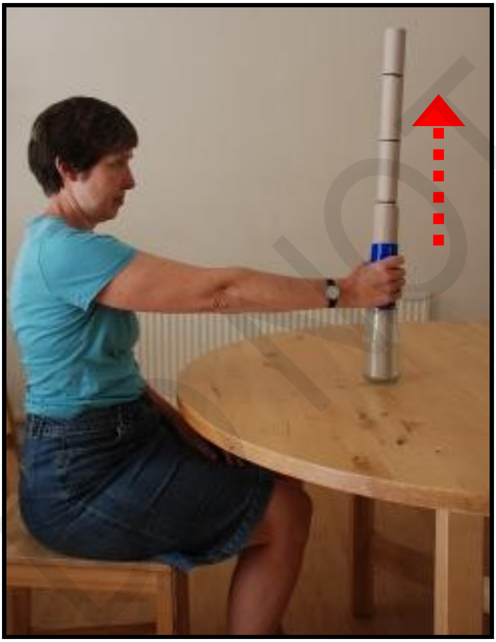

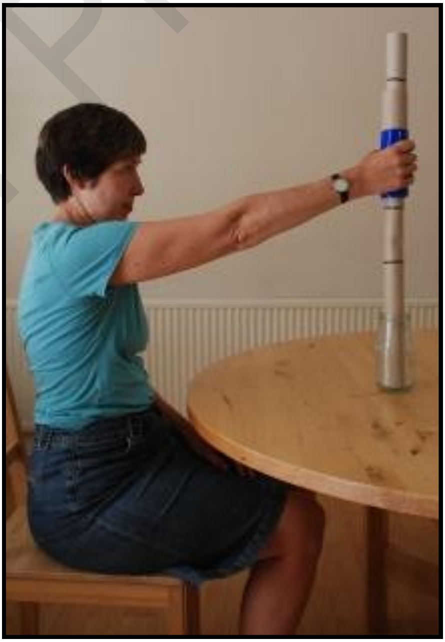

1.c.1

Reach back for door handle

- Stand with your back to a door
- Keep your elbow straight and reach back to touch or grasp the door handle
- Keep standing up tall throughout

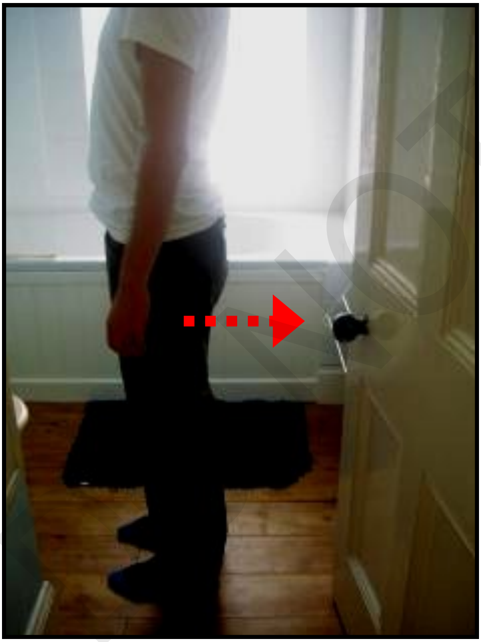

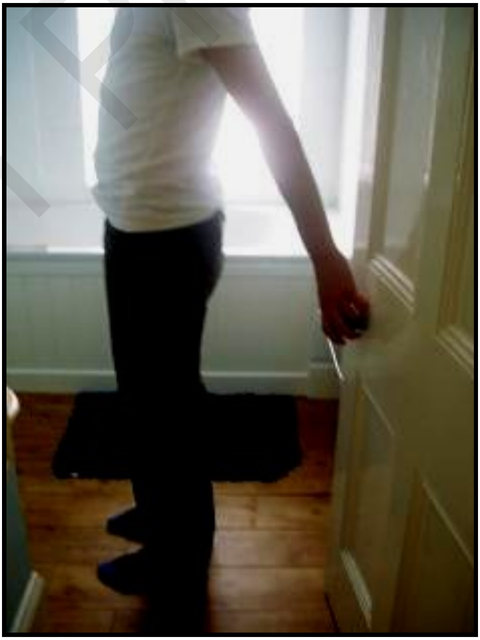

NOT TO PRINT

Pull drawer out

- Sit on a kitchen chair with your hand resting on the edge of a drawer or around the drawer handle
- Pull the drawer out towards you then slide back again
- Try to stay sat upright with your back touching the chair

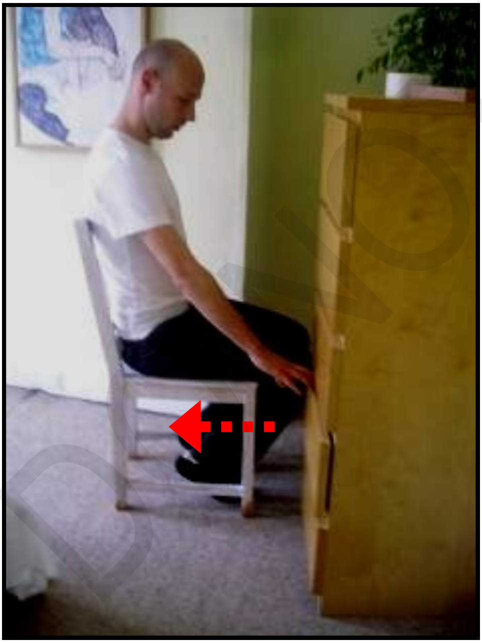

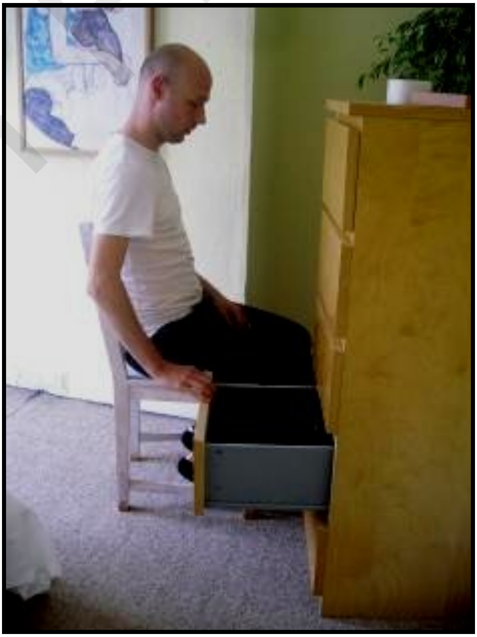

1.d.1

Reach to touch alarm clock

- Lay on your back with your arm resting on the bed - hand up
- Reach out to the side to try to touch your alarm clock / bed side table / etc.
- Try to keep your elbow straight and the back of your hand in contact with the bed
- Your carer / therapist may need to assist you to complete the movement

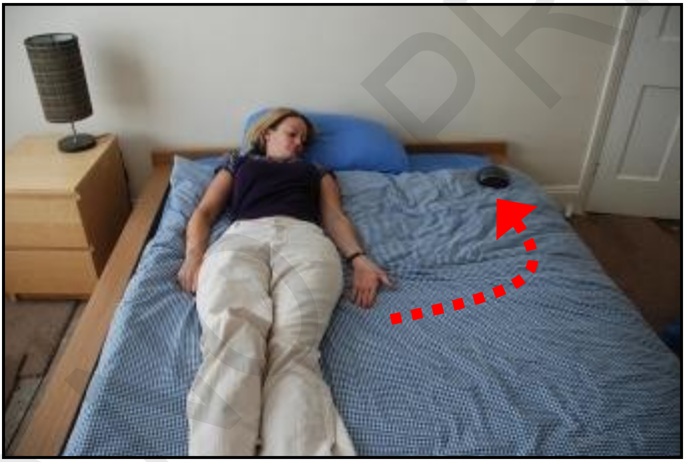

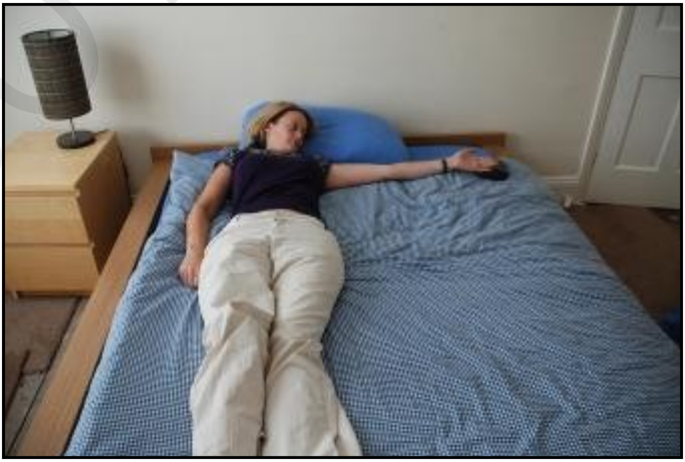

DO NOT PRINT

Slide arm along bed up to wall or headboard

- Lay on your back with your arm resting on the bed - hand up
- Trying to keep the back of your hand in contact with the bed, reach above your head to touch the headboard or wall.
- Your carer / therapist may need to assist you to complete the movement

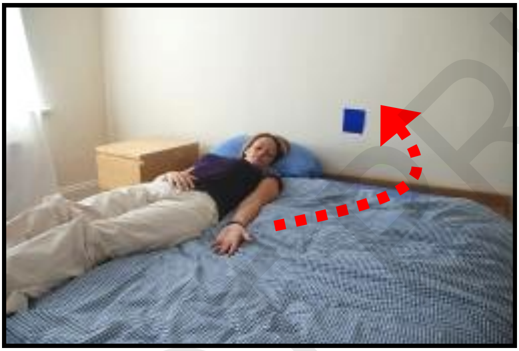

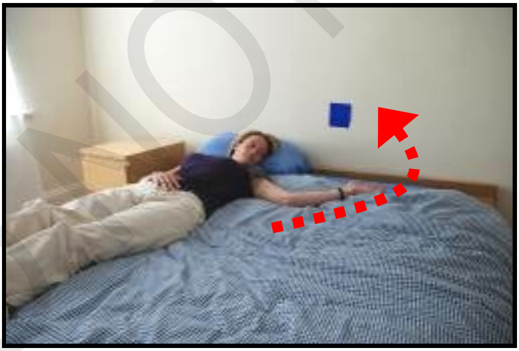

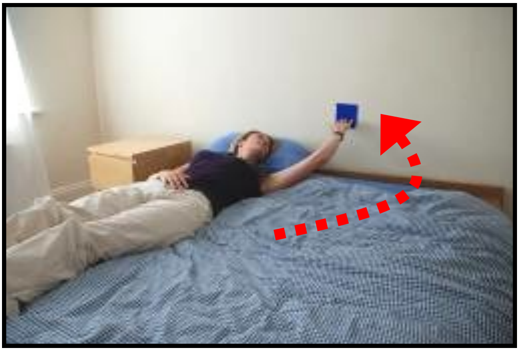

DO NOT PRINT

1.d.3

Push book away

- Sit side on to a table with your forearm supported on the table
- Place an object, for example a book, next to your forearm
- Try to slide your forearm along the table to push the object to a ‘target’. The ‘target’ could be the edge of the table or another object placed on the table.
- Try to keep facing forward throughout the exercise

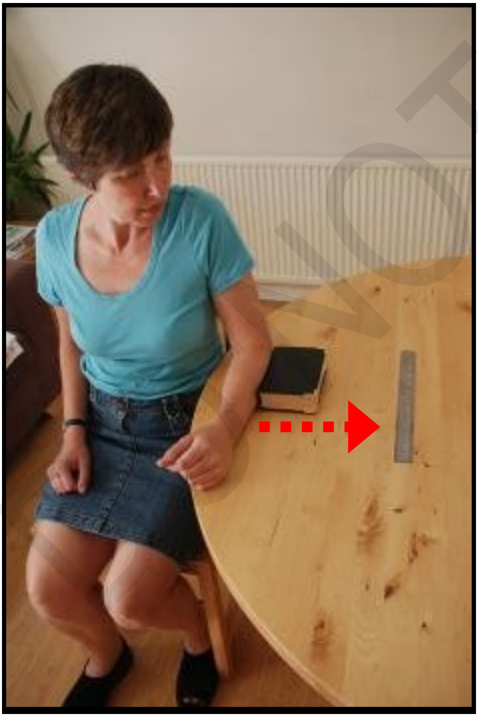

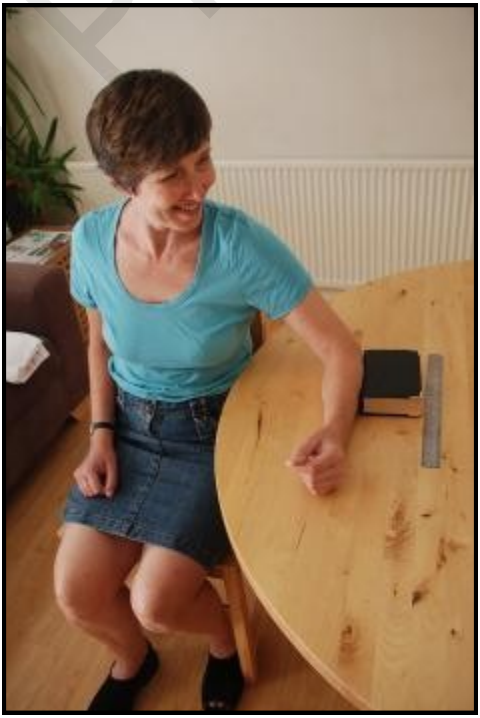

1.d.4

Standing and reaching to the side

- Stand with your feet slightly apart and a ‘target’ at shoulder height next to your.
- Practice reaching out to the side to touch or grasp the ‘target’.

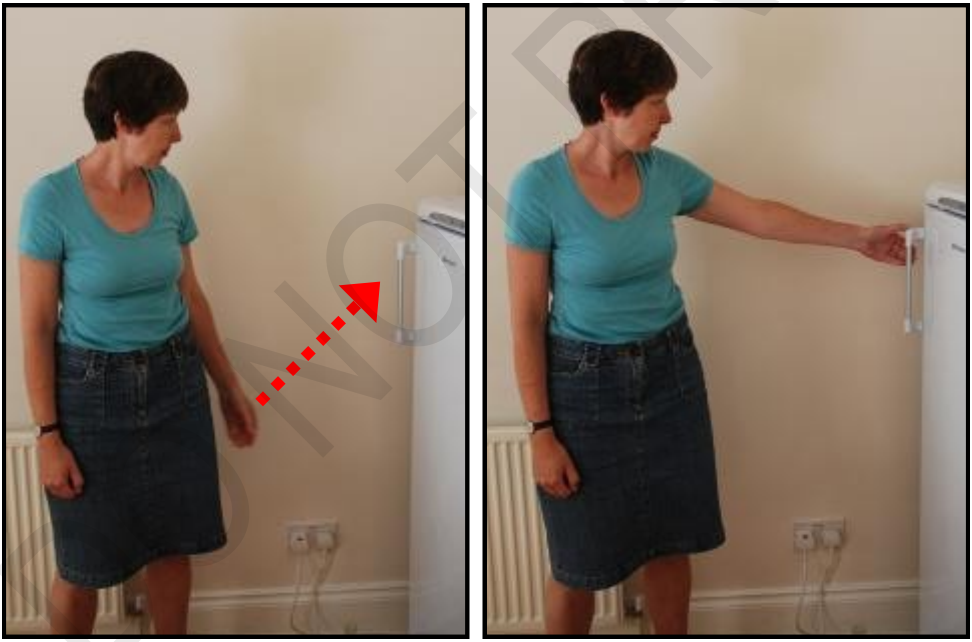

DO NOT PRINT

1.e.1

Turn hand over to tap 'target's

- Lay on your back
- Place 2 objects either side of your hand, for example, 2 tumblers.
- Hold a longer object in your hand, for example, a wooden spoon.
- Keeping your elbow straight, tap the objects either side with the longer object.

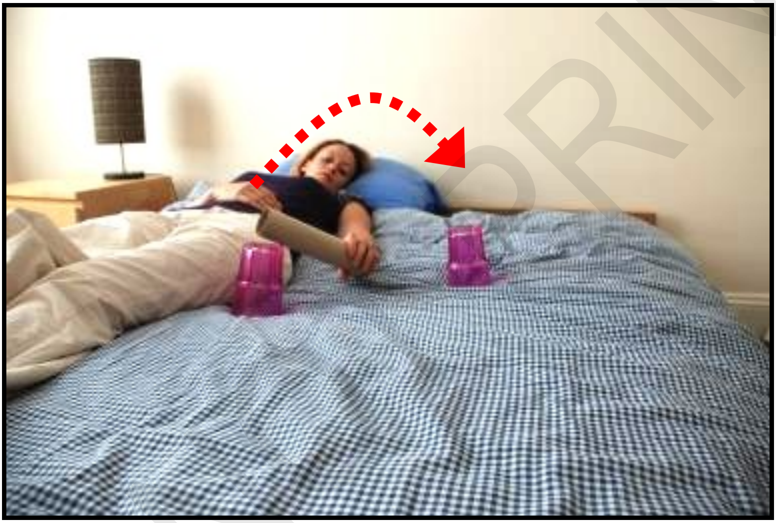

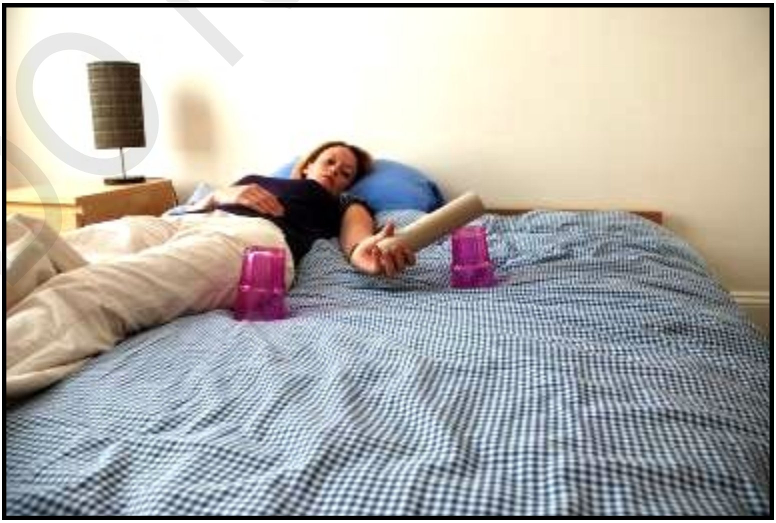

DO NOT PRINT

Knock on wall

- Lay on your back with a wall or the back of the sofa to your side
- Start with your elbow bent and your hand resting on your abdomen
- Keeping your elbow tucked in to your side move your hand to touch the wall / back of sofa then return to the starting position

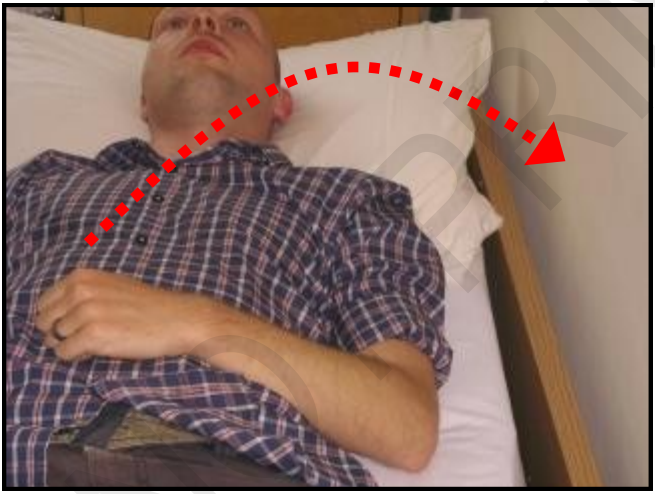

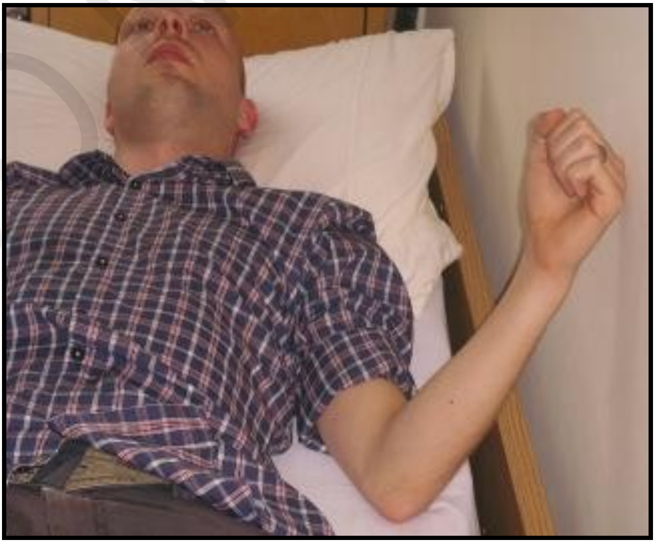

DO NOT PRINT

1.e.3

Knock on headboard

- Lay on your back with your headboard or the wall above you
- Start with your elbow out to the side and your hand resting on the bed
- Keeping your elbow in contact with the bed move your hand to touch the headboard / wall above you then return to resting on the bed

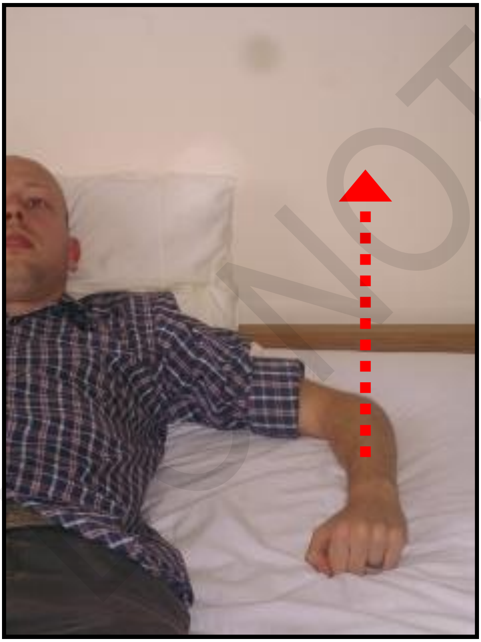

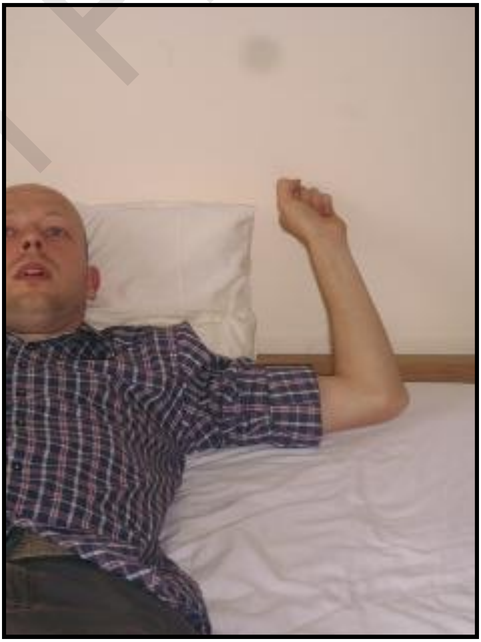

DO NOT PRINT

1.e.4

Knock object off table

- Sit at a table with your elbow supported on the ‘arm skateboard’ provided by your therapist
- Position an object, for example, a plastic bottle to the side of you by the edge of the table
- Try to keep your back in touch with the chair and move your hand to knock the bottle off the table.

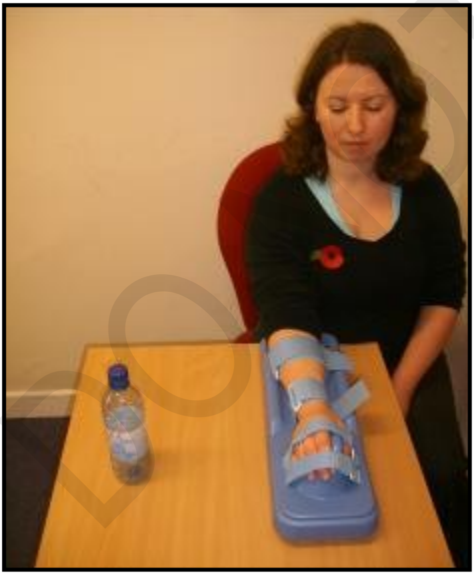

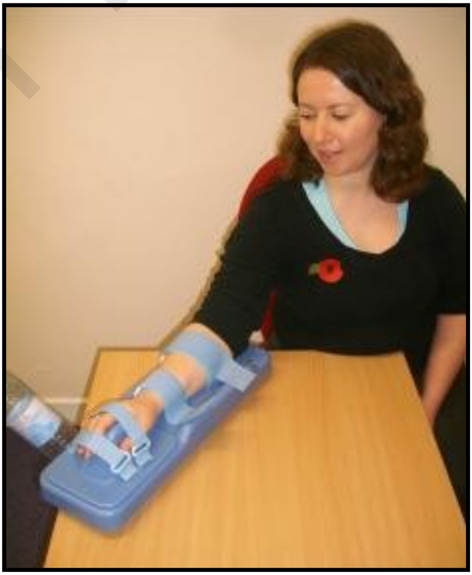

DO NOT PRINT

Move hand out to a 'target'

- Sit with your elbow bent at right angles and a piece of paper in between your upper arm and the side of your chest.
- Holding a long object practice moving your hand outwards to a table beside you keeping the long object upright
- Ensure that your back is kept against the chair and the piece of paper is not dropped

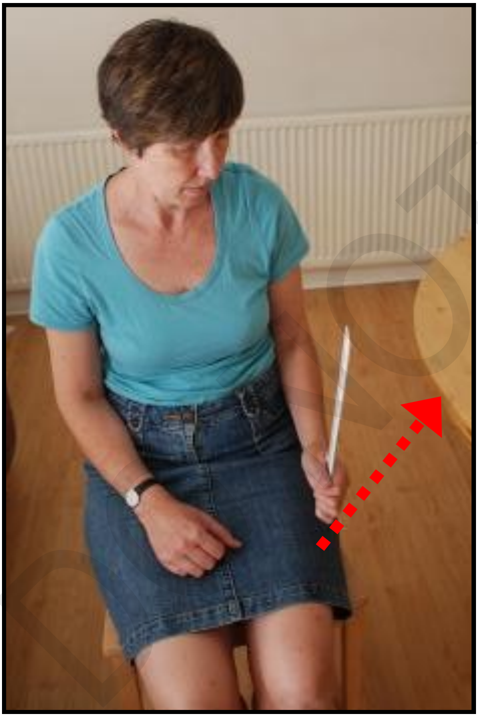

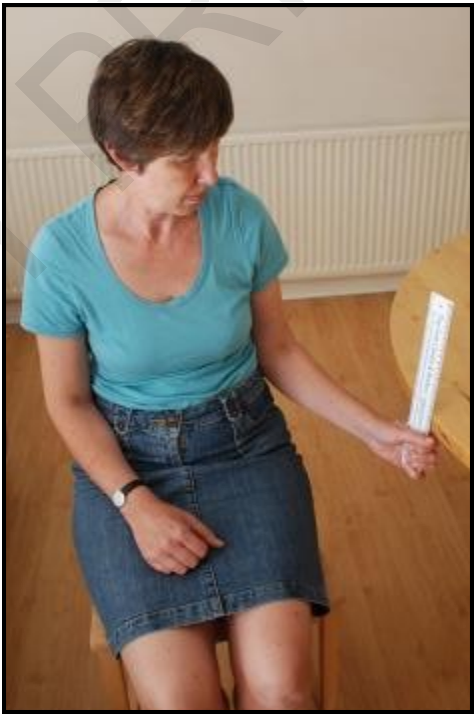

DO NOT PRINT

1.e.6

Move cup to table

- Sit with your elbow bent at right angles and a piece of paper in between your upper arm and the side of your chest.
- Practice moving a cup of water outwards to a table beside you without spilling it.
- Ensure that your back is kept against the chair and the piece of paper is not dropped.

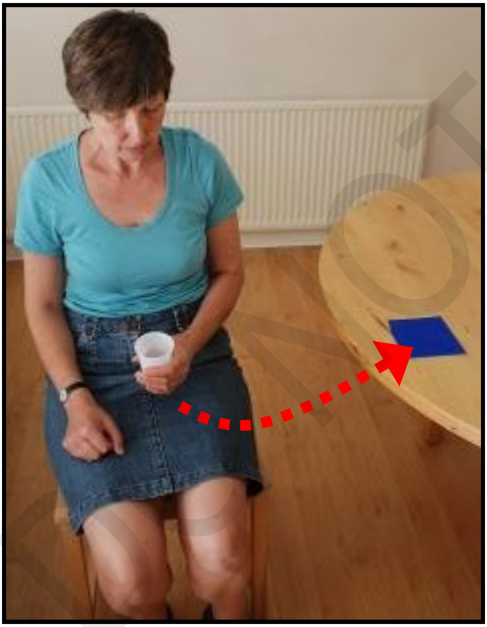

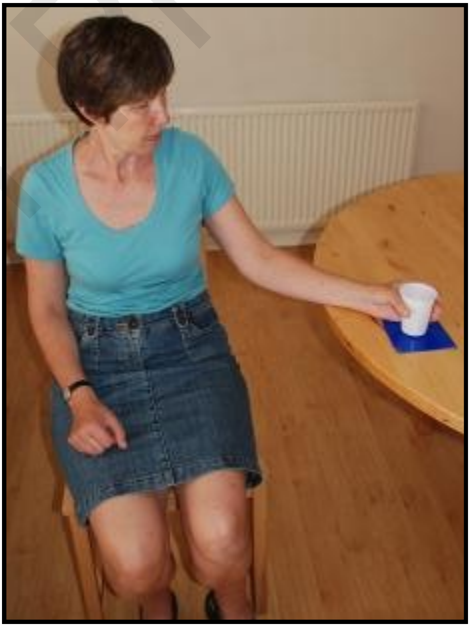

DO NOT PRINT

Polishing table

- Sit at a table with your forearm supported on the table and a polishing cloth under your hand
- Position a 'target' in front of you and slightly to the side
- Keep your elbow tucked in to your side and polish the table by moving the cloth to the 'target'

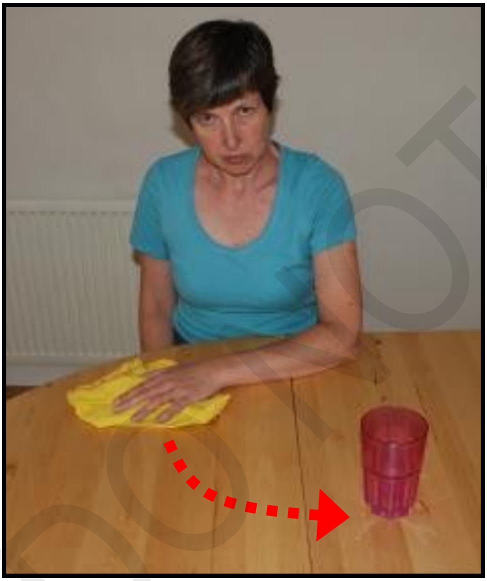

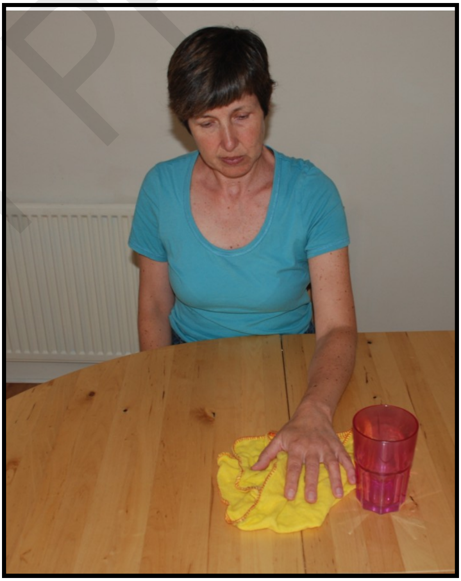

DO NOT PRINT

Knock rubbish into bin

- Sit at a table with your elbow supported on the table
- Position a bin to the side of you by the edge of the table
- Arrange a number of items on the table in front of you to knock into the bin, for example , scrunched up newspaper.
- Keep your elbow still and knock the 'rubbish' into the bin.

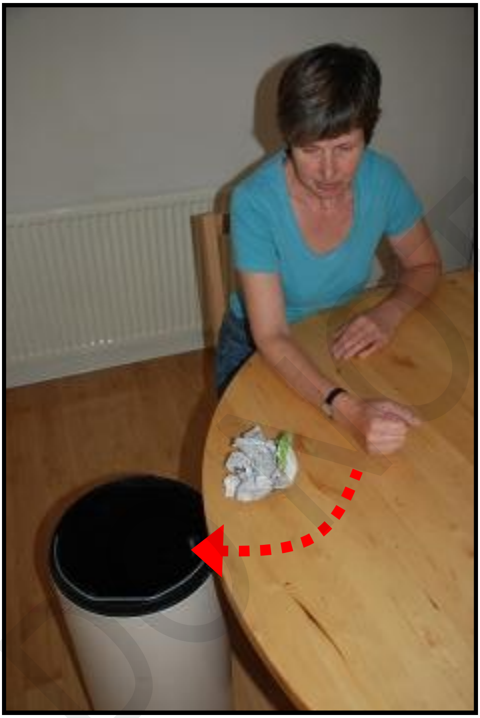

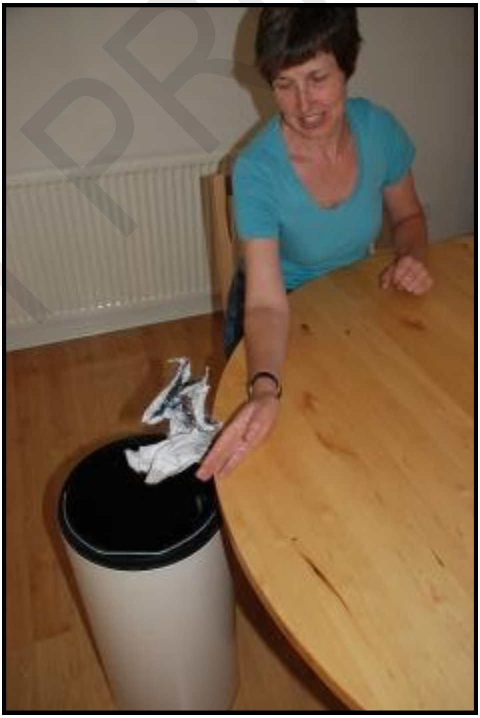

DO NOT PRINT

1.e.9

Move cup around ‘protractor’

- Sit side on to a table with your forearm supported on the table
- Place ‘protractor’ under forearm with edge in line with forearm.
- Place object, for example a cup, in your hand
- Keeping elbow in corner of ‘protractor’ move cup around protractor as far as you are able

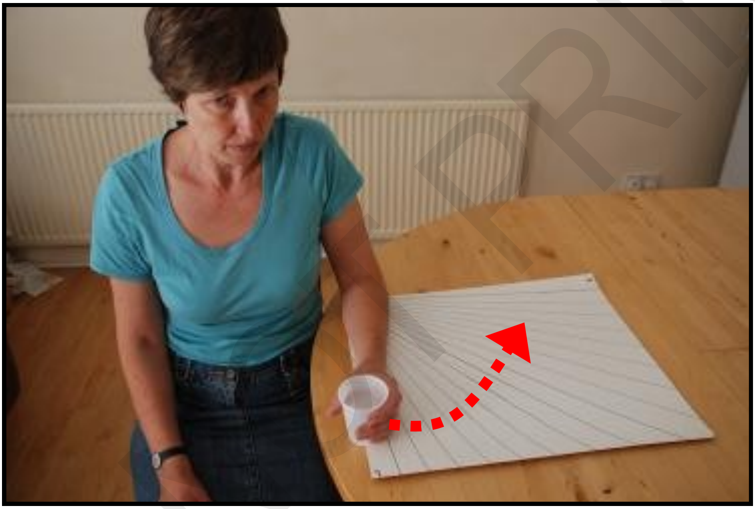

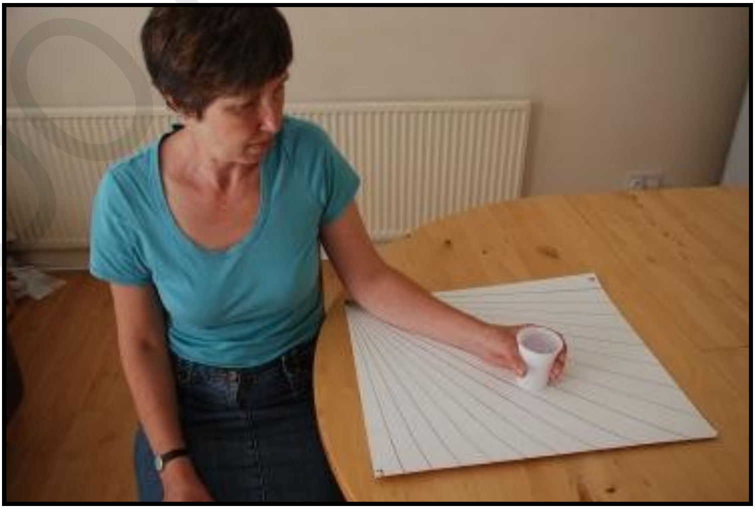

DO NOT PRINT

Knock on door

- Stand side on to a door with your elbow bent and your hand touching your abdomen
- Keeping your elbow tucked in move your hand to touch the door and then back to your abdomen

DO NOT PRINT

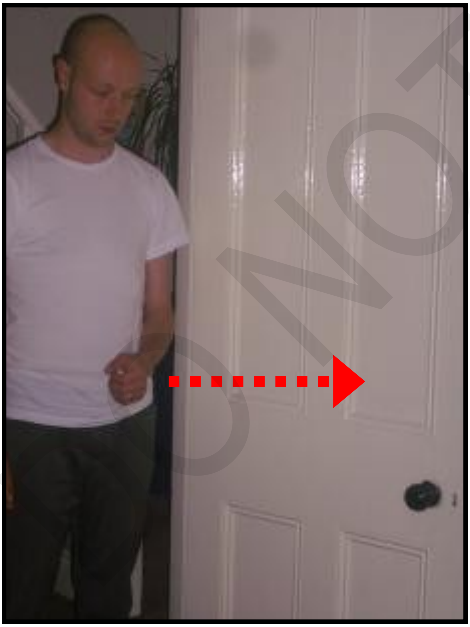

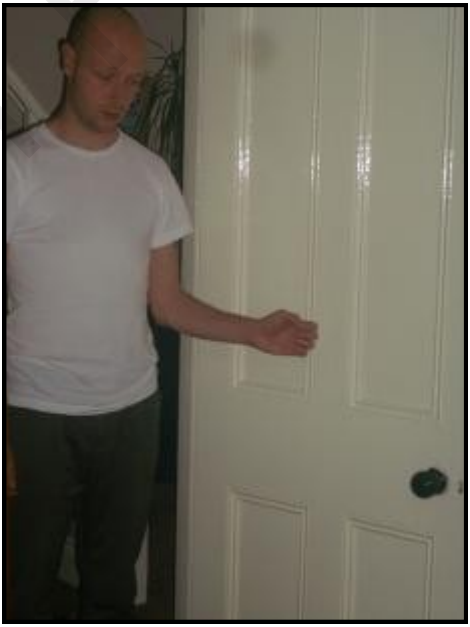

Draw curved lines on paper

- Sit at a table with your elbow supported on the table
- Underneath your forearm place a piece of paper with 2 'target's drawn on that your therapist will have prepared for you.
- Keeping your elbow still, draw curved lines on the paper between the 2 'target's
- You may need to prevent the paper sliding with your other arm.

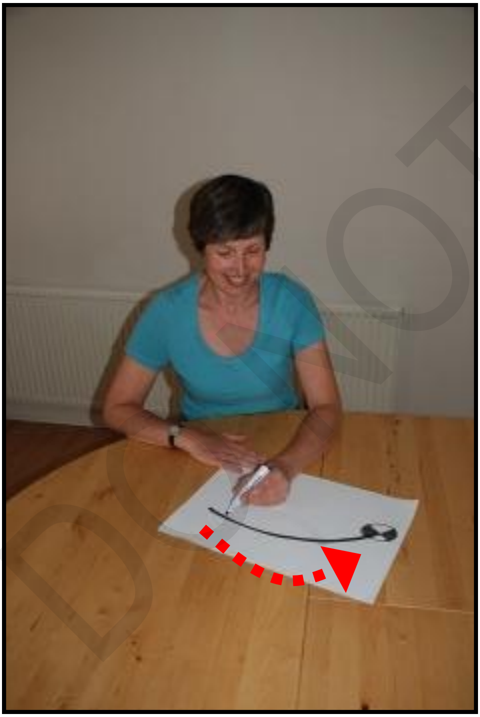

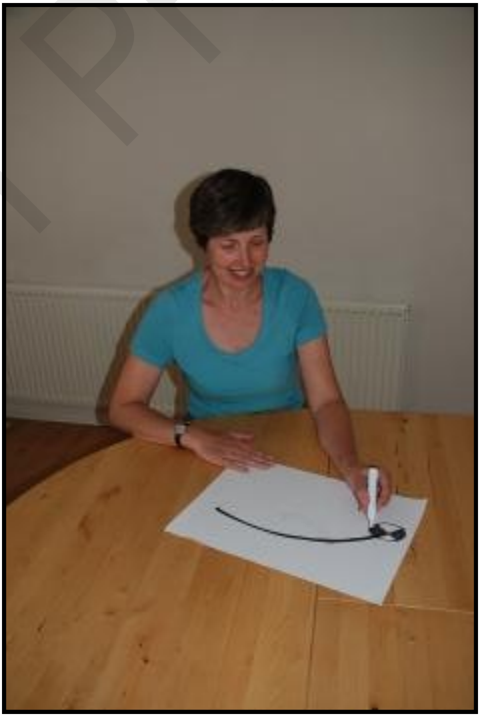

DO NOT PRINT

1.e.12

Pull out tape measure

- Sit at a table with your elbow supported on the table and hold the ends of a tape measure in your hands.
- Position a ‘target’ in front of you and slightly to the side
- Keeping your elbow still and pull out the tape measure to touch the ‘target’

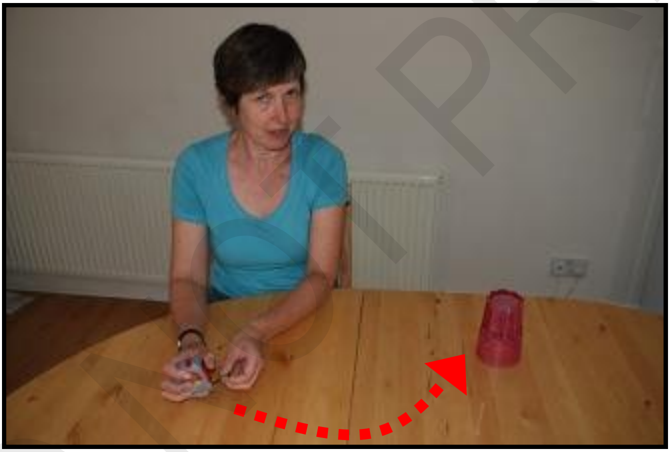

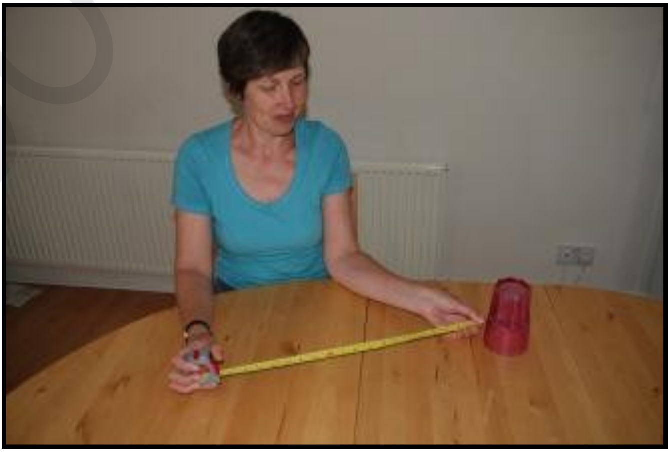

DO NOT PRINT

Pull drawer out

- Stand side on to a set of drawers
- Place your hand on the edge of a drawer or around the drawer handle
- Keeping your elbow tucked in pull the drawer out to the side then slide back again

- **Mind your fingers to ensure they do not get trapped in the drawer**

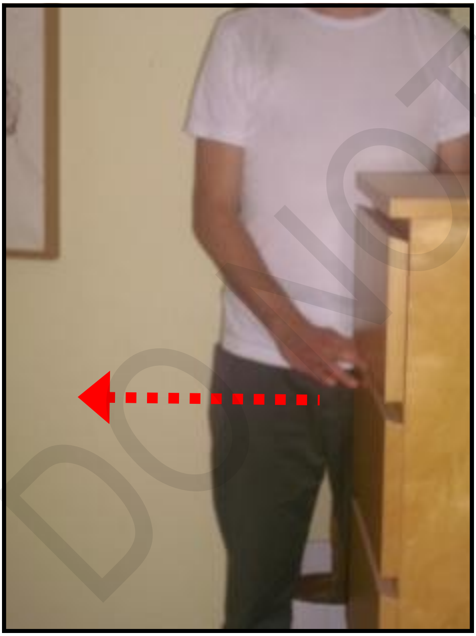

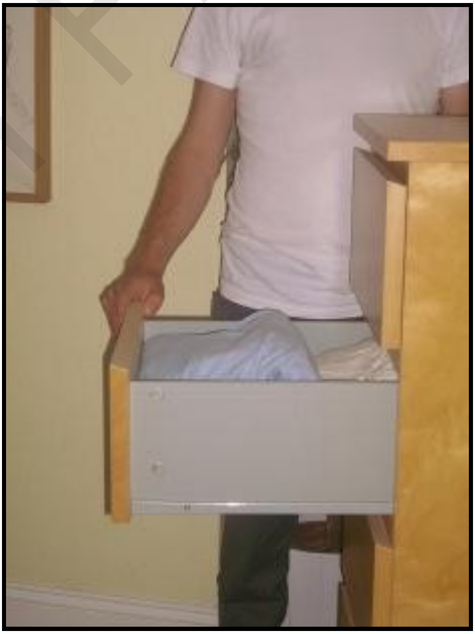

DO NOT PRINT

Move object along table

- Sit at a table with your elbow supported on the table and a cup in your hand
- Position a saucer in front of you and slightly to the side
- Try to keep your elbow still and place the cup onto the saucer.

DO NOT PRINT

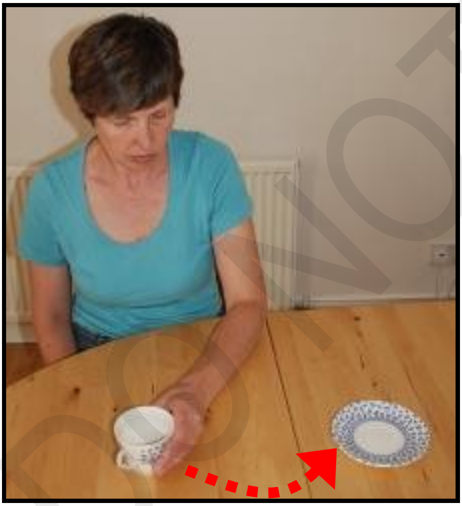

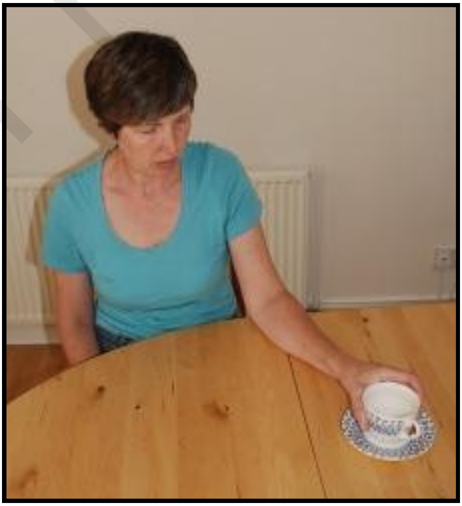

Slide hand up and down broom handle

- Sit on a chair with your weaker hand around a broom handle
- You can support the broom with your other arm to keep it vertical if needed
- Slide your hand as far up the broom as possible
- Lower your hand back down the broom as **slowly** as possible.

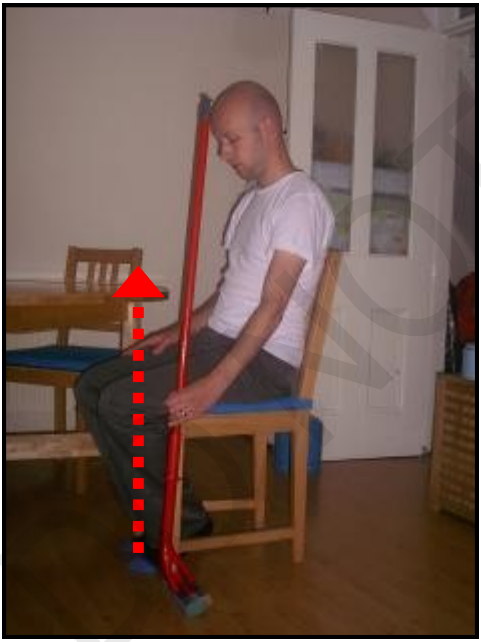

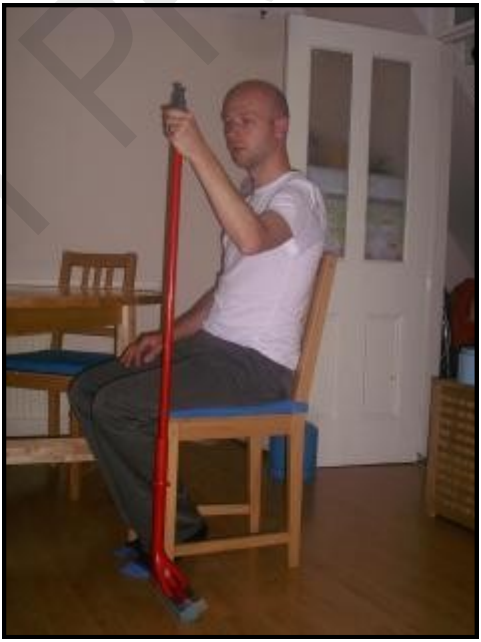

2.a.2

Pick up and replace phone

- Sit at a table with a telephone on
- Place your hand onto the telephone handset
- Lift the handset and take it to your ear
- Replace the handset as slowly and carefully as possible

DO NOT PRINT

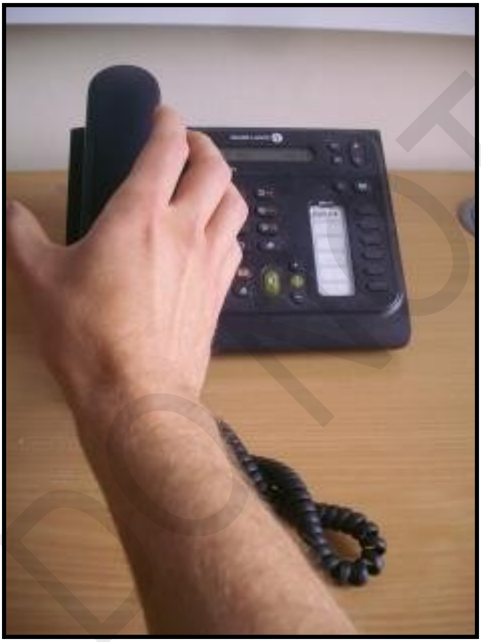

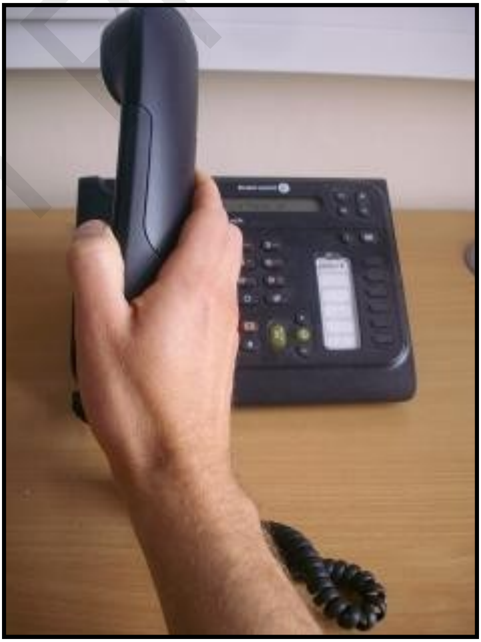

2.a.3

Move pan forwards and backwards on oven hobs

- Stand in front of a cooker
- Hold a pan in your hand
- Move the pan forwards and backwards between the front and back hobs

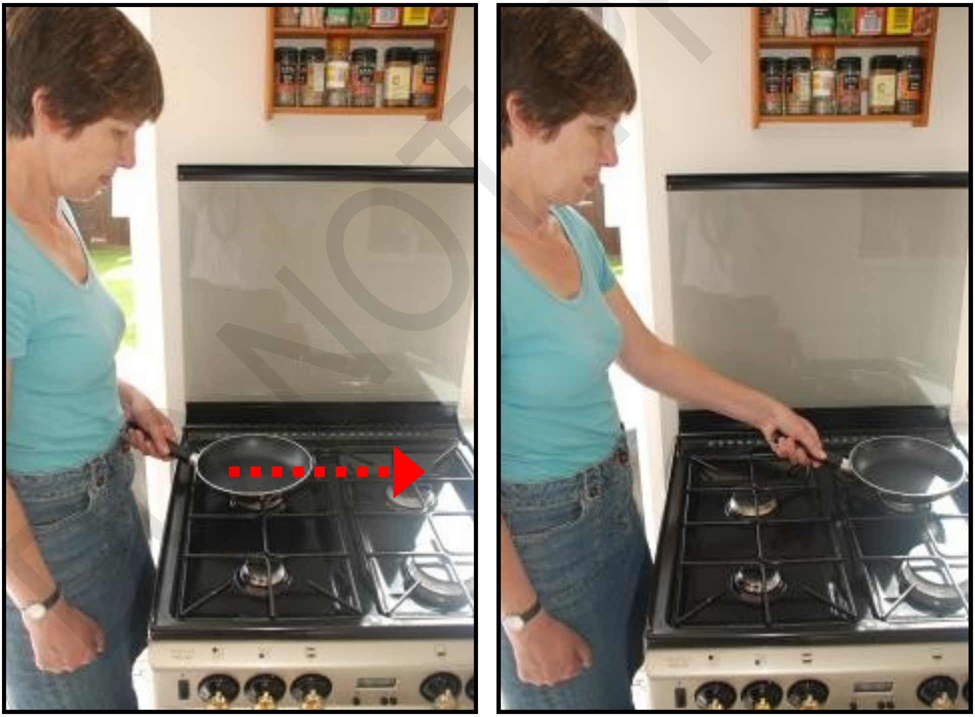

Move object between shelves

- Sit or stand in front of a set of shelves
- Place an object, for example a book, in your hand
- Move the object between different height shelves

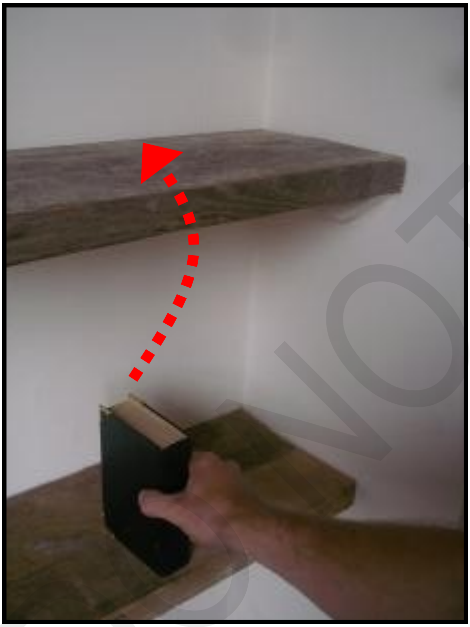

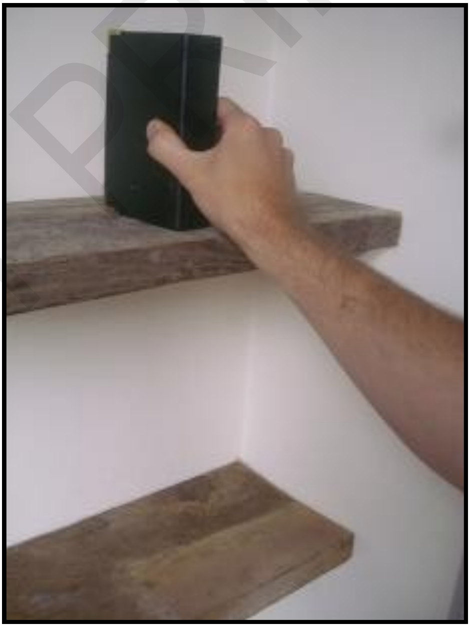

DO NOT PRINT

2.b.1

Sliding arm forward

- Sit at a table with your forearm supported on a towel or low resistance fabric
- Place an object in your hand
- Try to slide your hand forward to touch the object to a ''target''. This could be the edge of the table or an object placed on the table in front of you

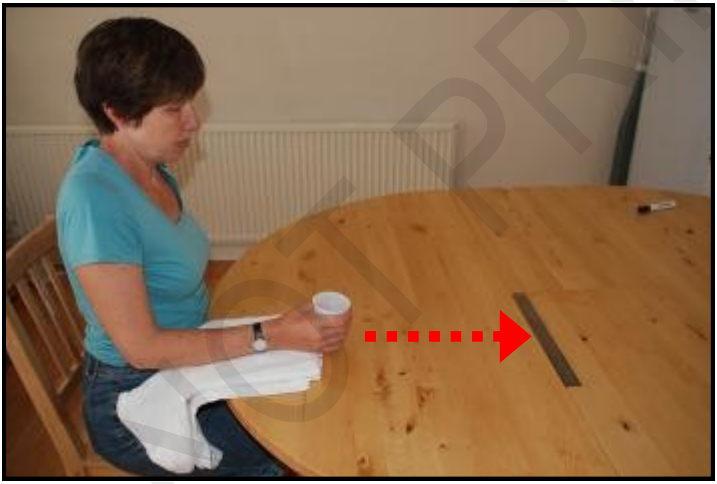

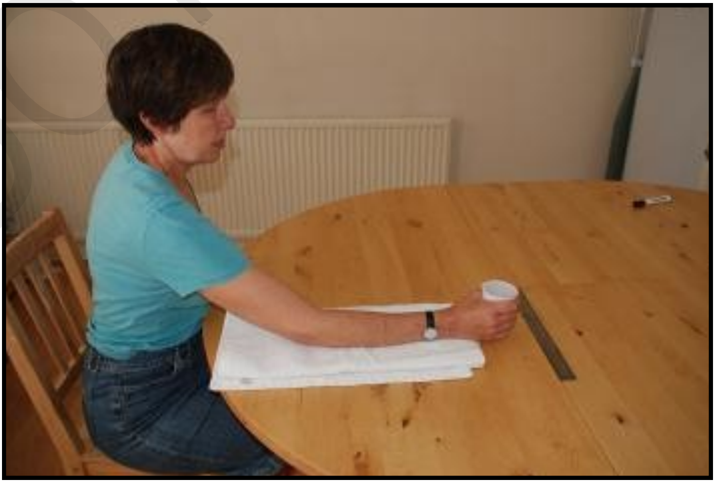

DO NOT PRINT

2.b.2

Push object forward

- Sit at a table with your forearm supported on the table and your hand resting on an object
- Try to slide your hand forward to touch the object to a ''target''. This could be the edge of the table or an object placed on the table in front

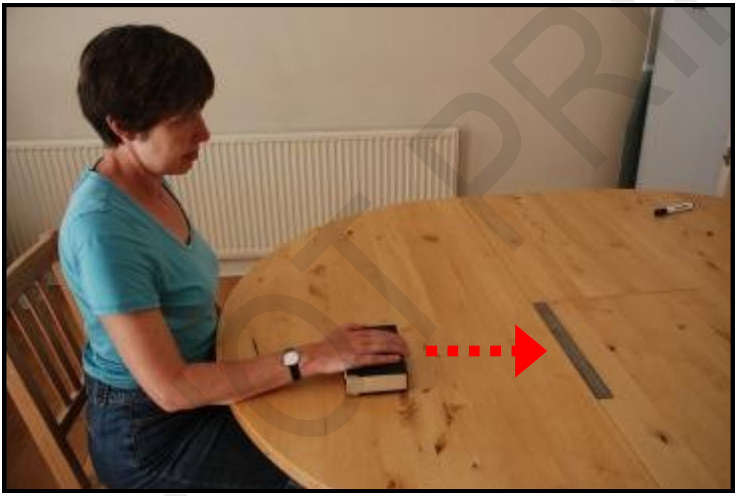

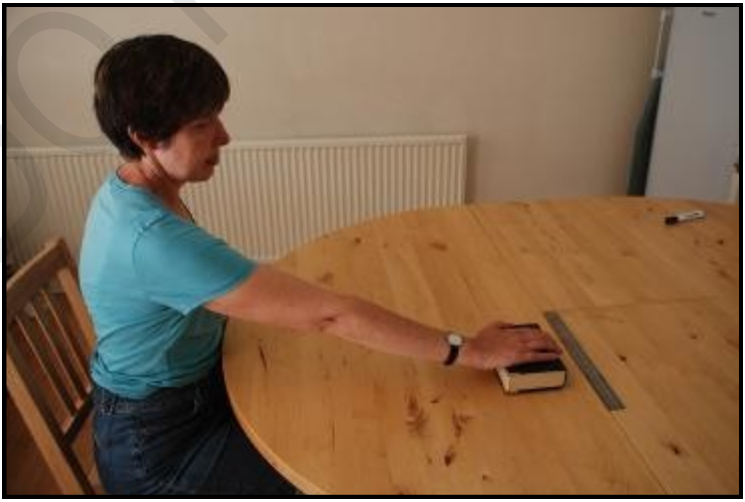

DO NOT PRINT

2.b.3

Lift tumbler onto table

- Sit side on to a table with your forearm resting on lap and a tumbler in your hand
- Lift your forearm and the tumbler onto the table
- Return your forearm and tumbler to your lap as **slowly** as possible.

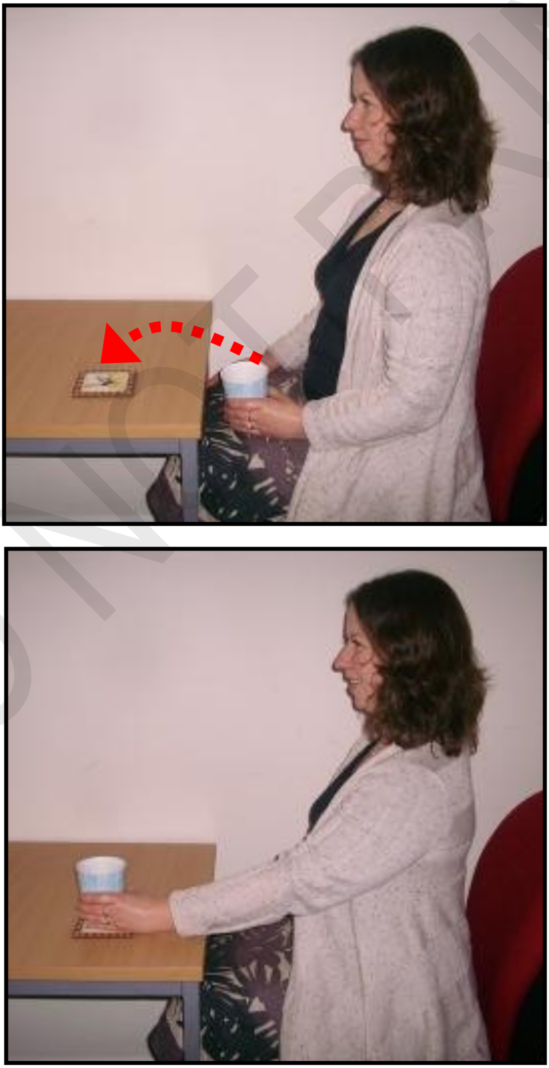

DO NOT PRINT

Reach for cupboard handles

- Stand in front of a set of cupboards
- Start with your arm resting by your side
- Reach to touch handles
- Try to finish with your elbow straight
- Keep your shoulders down and level
- Lower your arm down by your side after **slowly**

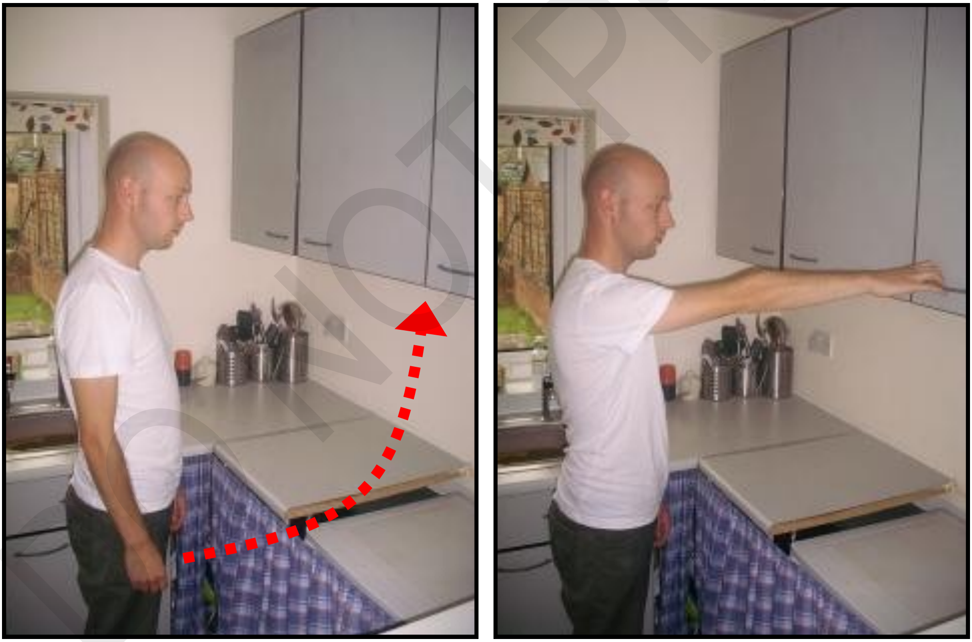

DO NOT PRINT

Move pan sideways on oven hobs

- Stand in front of a cooker
- Hold a pan in your hand
- Move the pan side to side between the hobs

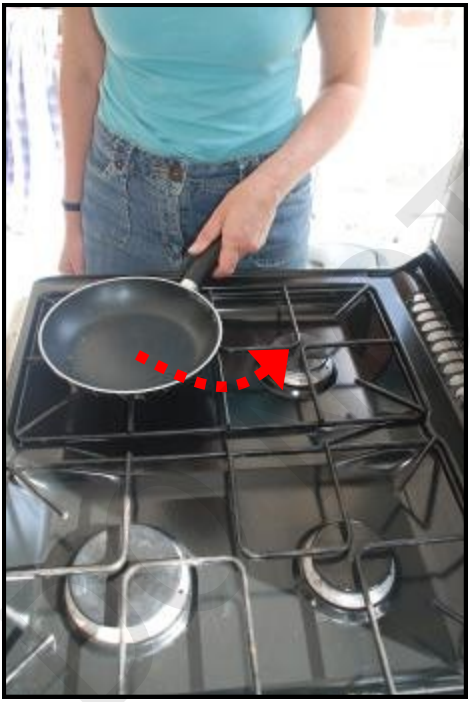

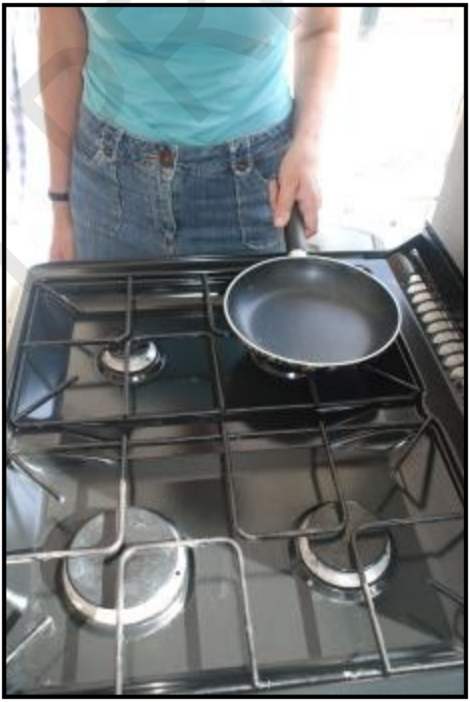

2.c.3

Move books between and along shelves

- Sit or stand in front of a set of shelves
- Place an object, for example a book, in your hand
- Move the object between different height shelves and along the shelves trying to straighten your elbow by reaching out to the side

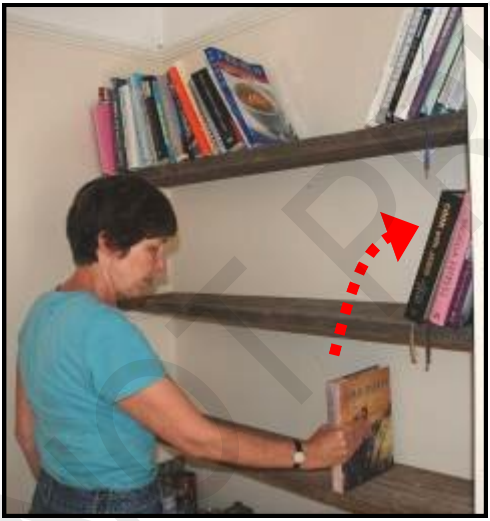

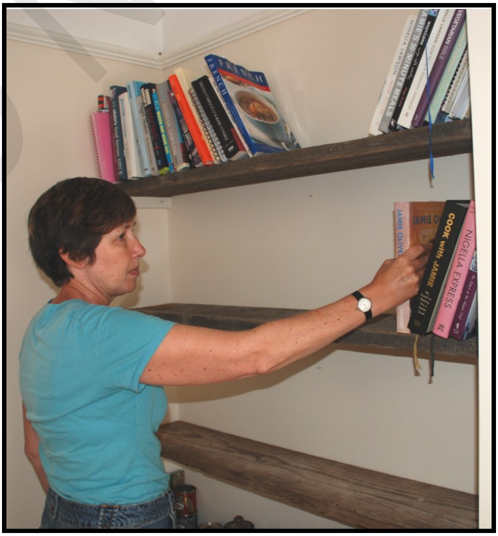

DO NOT PRINT

Reach to different 'marker's

- Sit in front of a table
- Your therapist will provide you with a large piece of paper with 'markers' on it.
- Place an object, for example a tumbler, in your hand.
- Practice moving the object from one marker to another

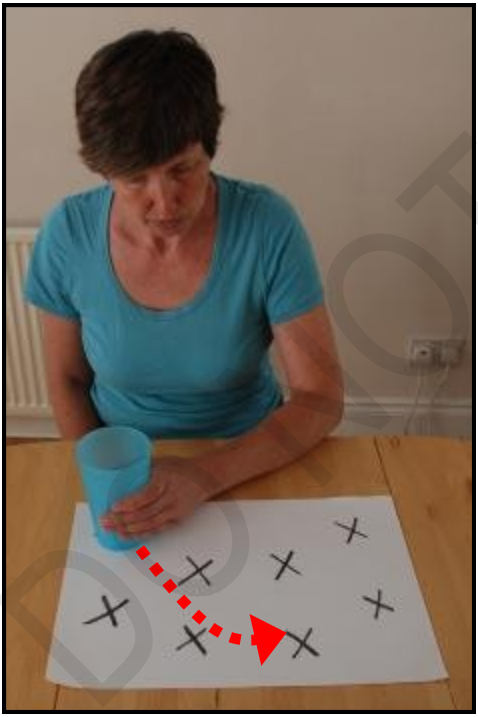

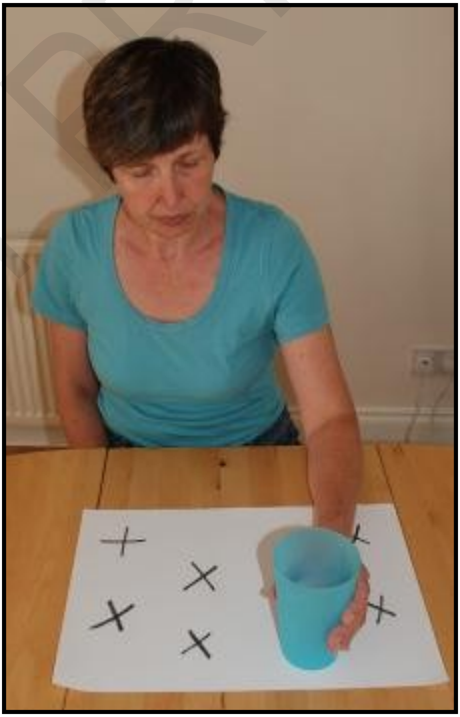

DO NOT PRINT

2.c.5

Move object between work surfaces

- Stand between 2 tables or worktops
- Practice lifting an object from one table /worktop to the other without dropping it.

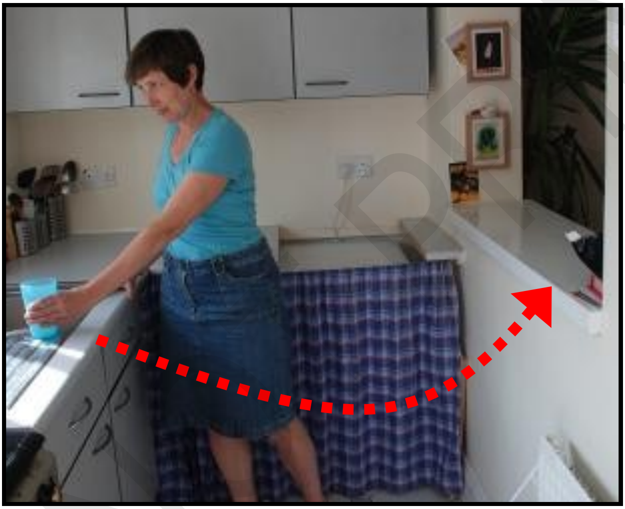

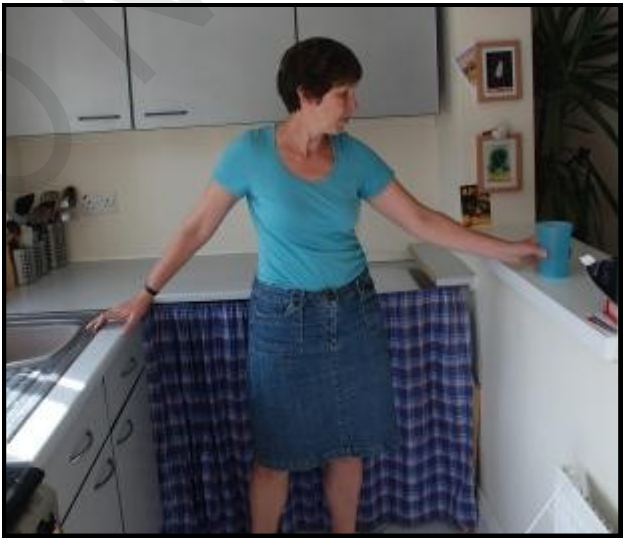

DO NOT PRINT

3.a.1

Reach from forehead to light cord

- Lay on your back
- Start with your hand on your forehead - your carer / therapist may need to assist you to achieve this position.
- Try to straighten your elbow to touch light cord
- Lower your hand back to your forehead slowly by bending your elbow
- Your carer / therapist may need to support your elbow while you do this exercise

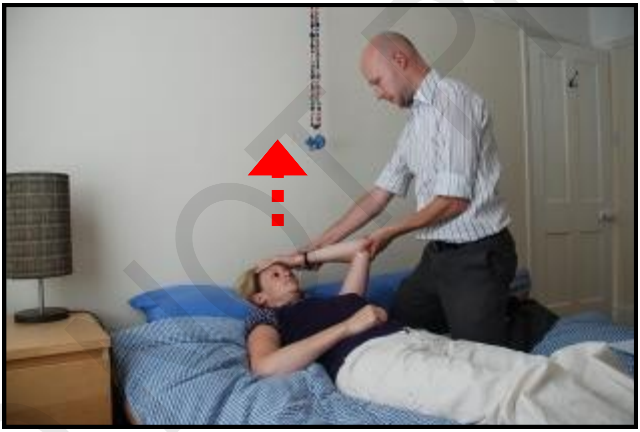

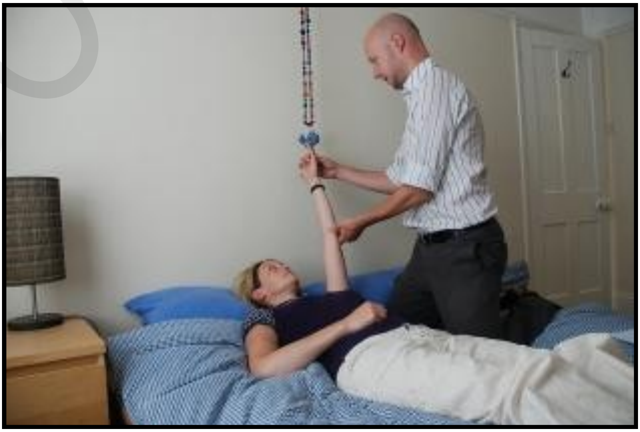

DO NOT PRINT

4.a.1

Rotate forearm to tap wall with object

- Sit next to a wall with a ruler in your hand and your forearm supported either on your lap or on a table
- Practice rotating your forearm back and forth to touch the ruler to a 'target' on the wall.
- Try to keep you elbow tucked in throughout

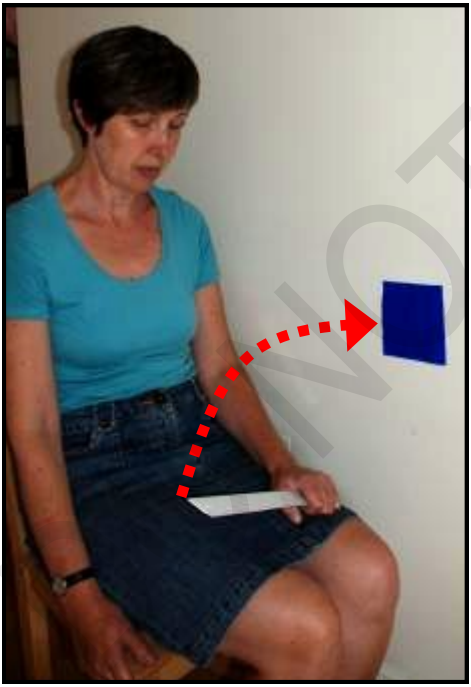

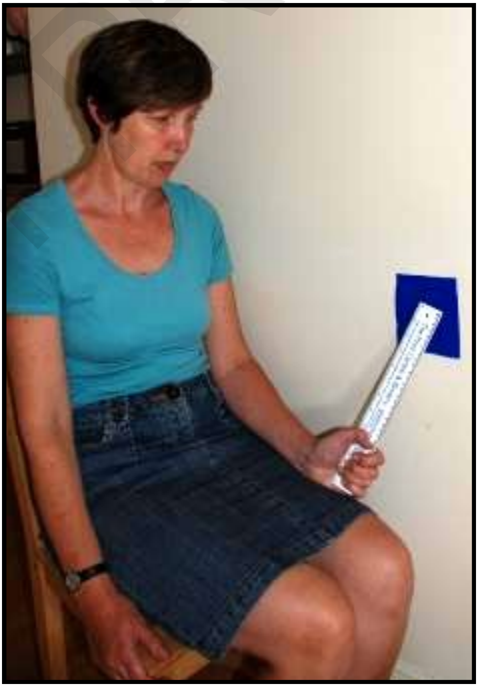

NOT TO PRINT

4.a.2

Rotate forearm to tap ‘target’s with object

- Sit in front of a table with your elbow on the table, a ruler in your hand and two ‘target’s either side of your wrist.
- Practice rotating your forearm to touch the ruler on each of the ‘target’s.
- Try to keep your elbow still and not to move your shoulder.

DO NOT PRINT

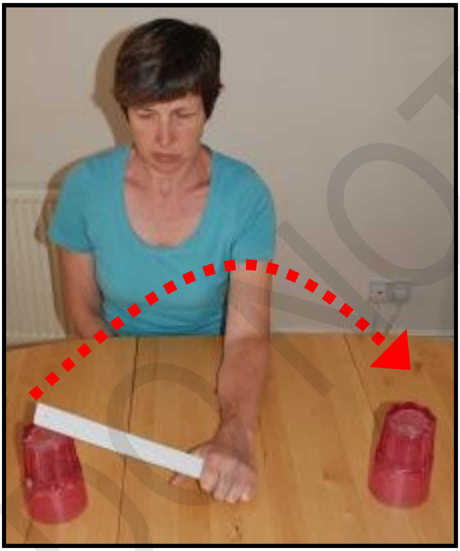

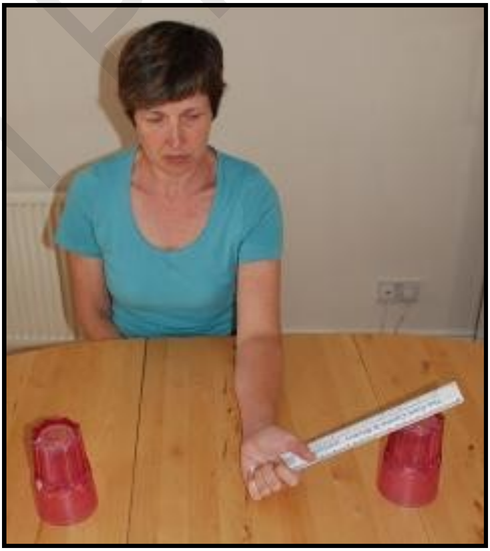

4.a.3

Turn an object over

- Sit at a table with your elbow supported
- Place an object, for example a book, in your hand
- Practice turning the object over

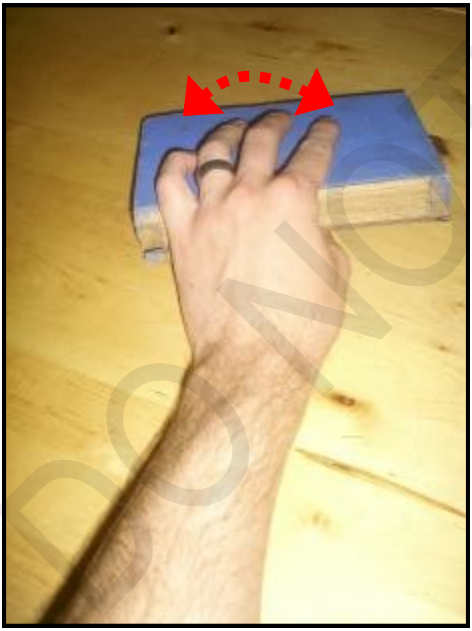

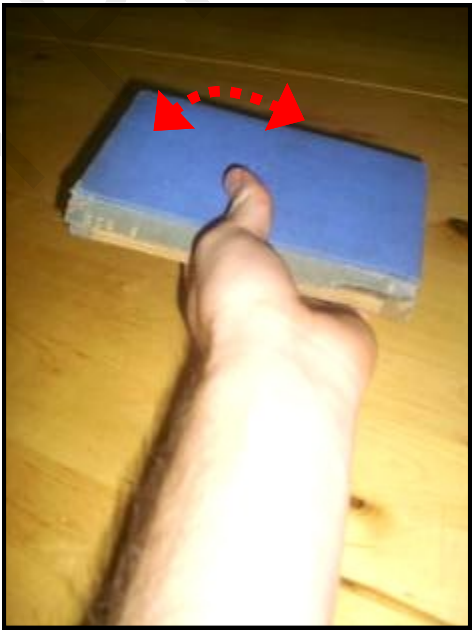

DO NOT PRINT

Empty pot of coins onto table

- Sit at a table with your elbow supported
- Hold a tumbler with coins in
- Rotate your forearm to empty the coins onto the table

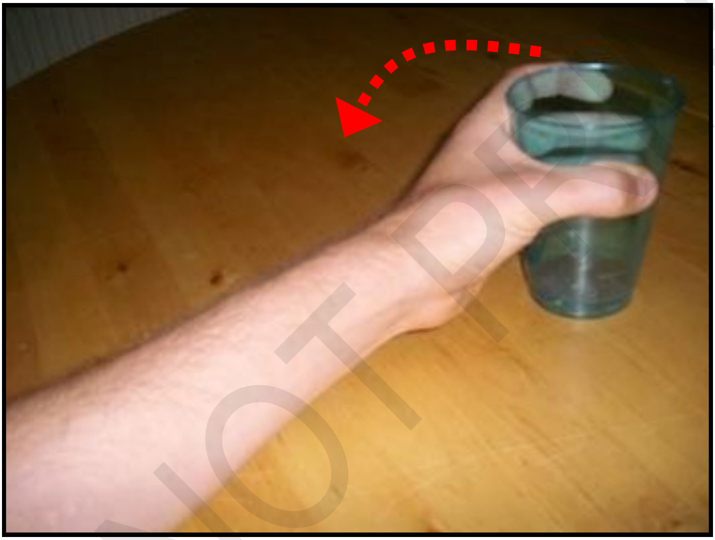

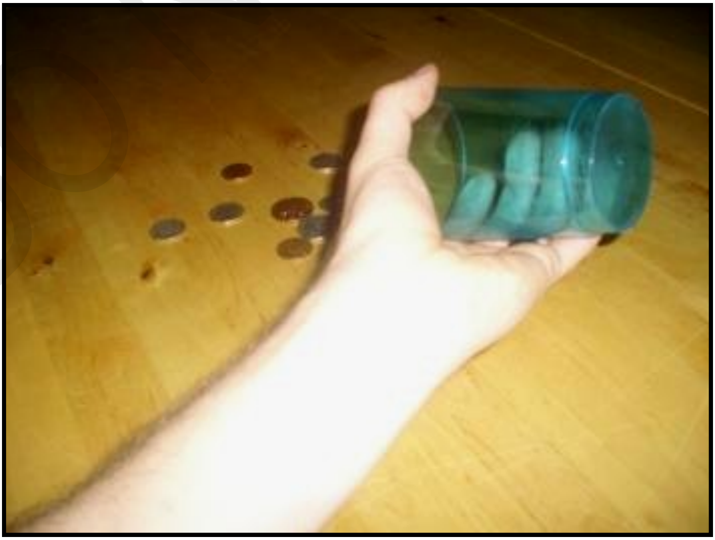

DO NOT PRINT

4.b.2

Empty water out of tumbler

- Sit at a table with your elbow supported and a large bowl as a 'target'
- Hold a tumbler with water in
- Rotate your forearm to pour the water into the bowl

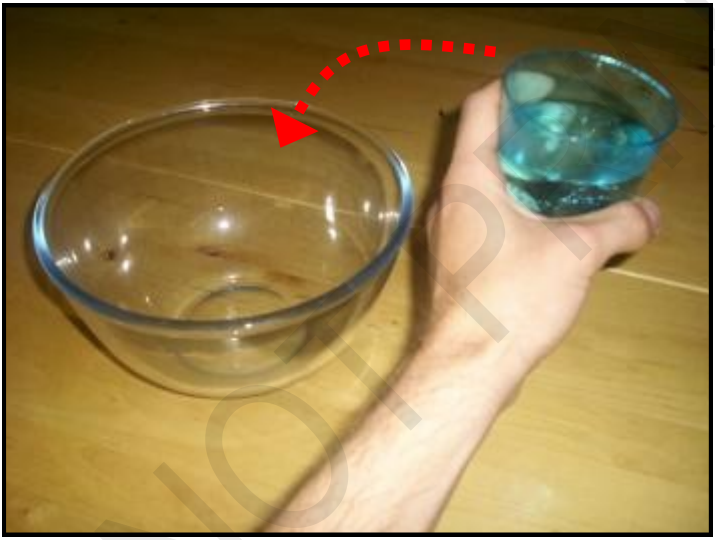

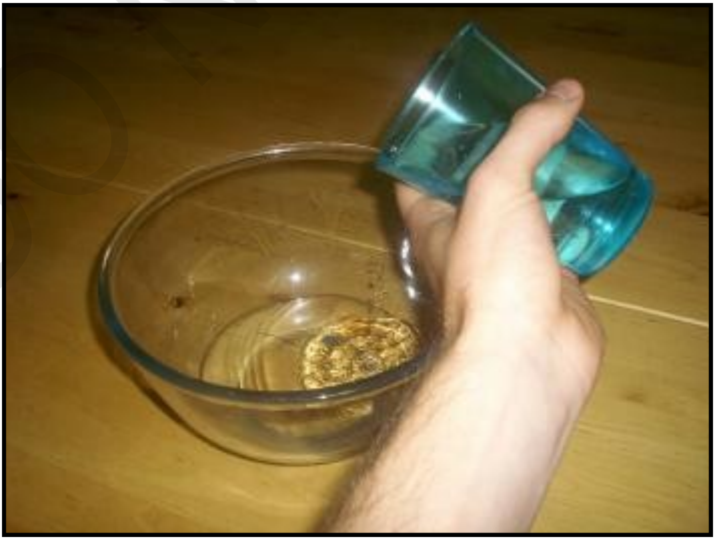

DO NOT PRINT

Dry tumbler with tea towel

- Sitting or standing
- Hold a tea towel in your weaker hand, place inside a tumbler and rotate to dry
- Stabilise the tumbler with your other hand

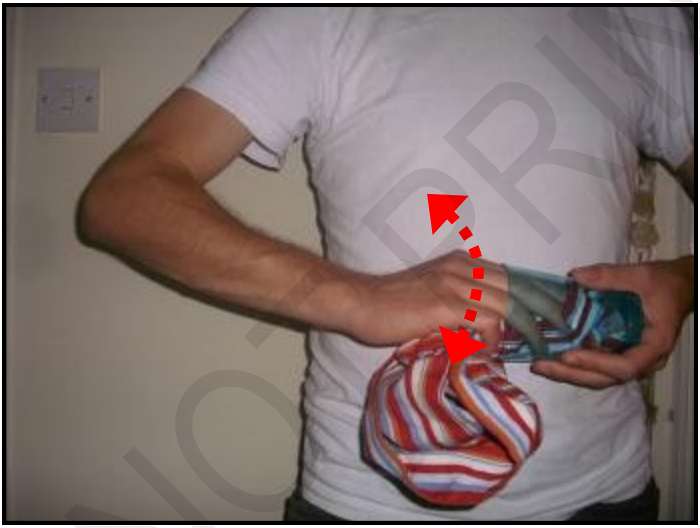

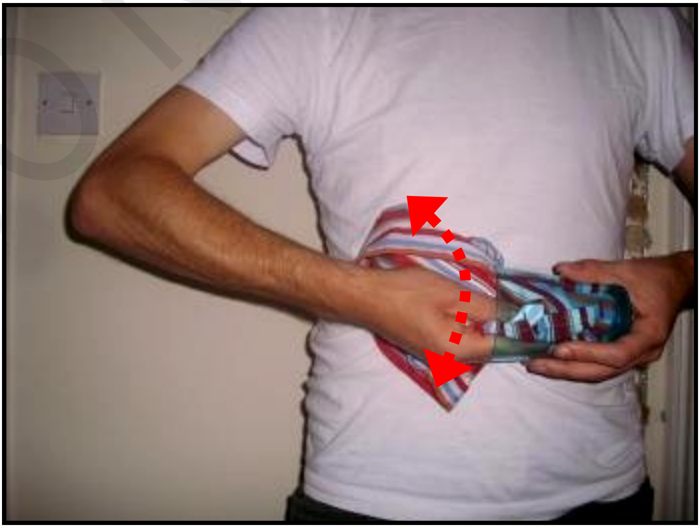

DO NOT PRINT

Wring out towel

- Hold a towel between both hands with both hands facing down
- Rotate the forearm of your weaker arm to wring out the towel
- Try to keep your other hand still

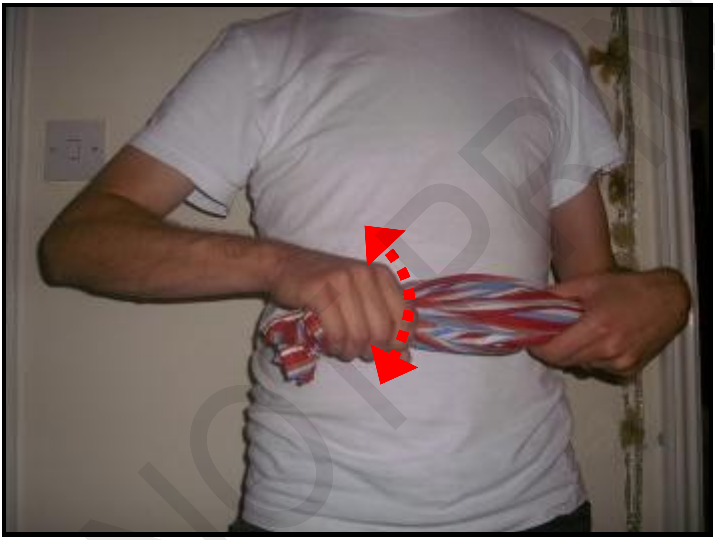

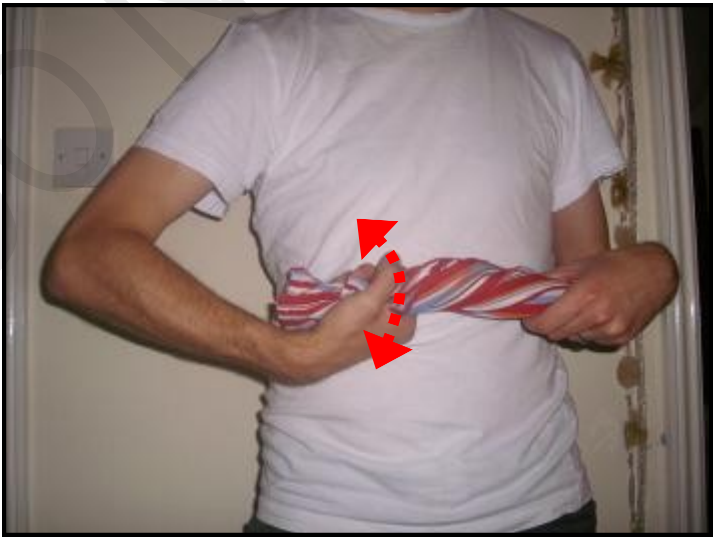

DO NOT PRINT

Drop object from one hand into the other

- Hold an object, for example a paper ball or tennis ball, in your weaker hand
- Drop the object into your other hand
- Swap your hands over for next try so you now catch with your weaker hand

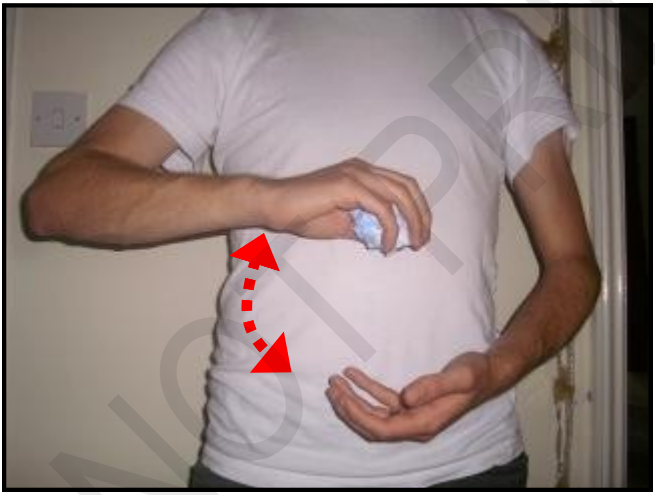

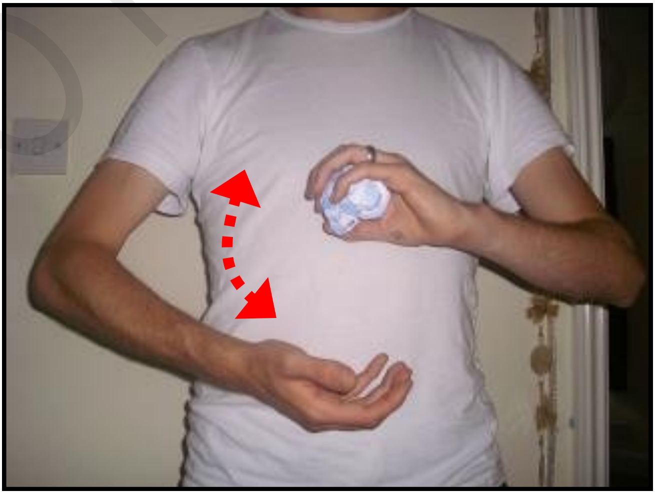

DO NOT PRINT

4.c.1

Lift bags from floor to table and back

- Stand next to a table or worktop with a bag on the floor
- Pick up the bag with your hand facing down
- Lift the bag and place on the table with your hand facing up
- Return to the floor and the hand facing down forearm position

DO NOT PRINT

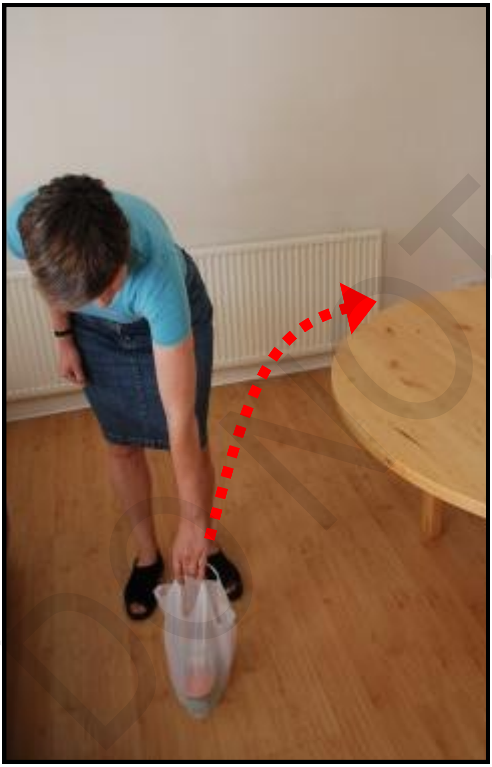

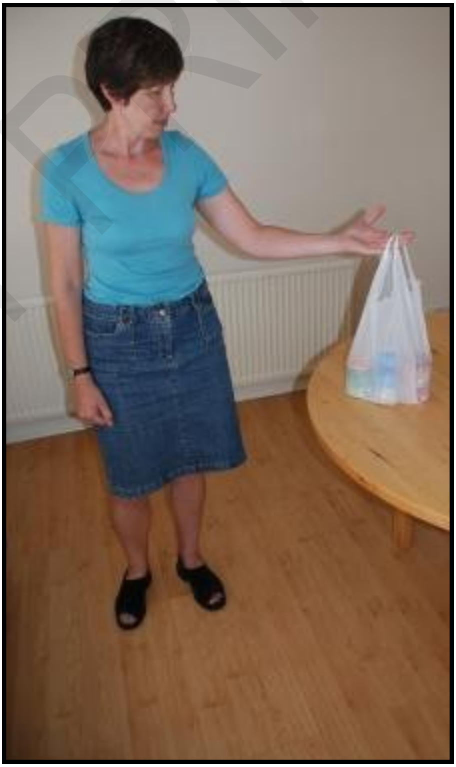

Pour kettle

- Stand in front of a work surface
- Hold a kettle in your hand
- Practice pouring **cold water only** into a cup
- You may need to stabilise the cup with other hand

DO NOT PRINT

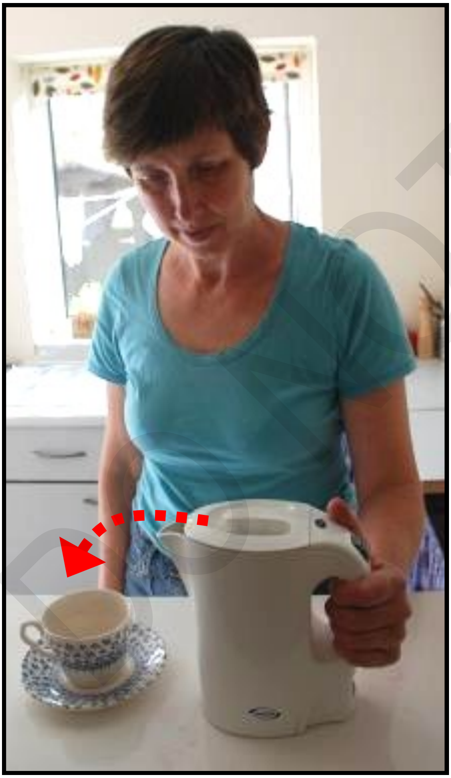

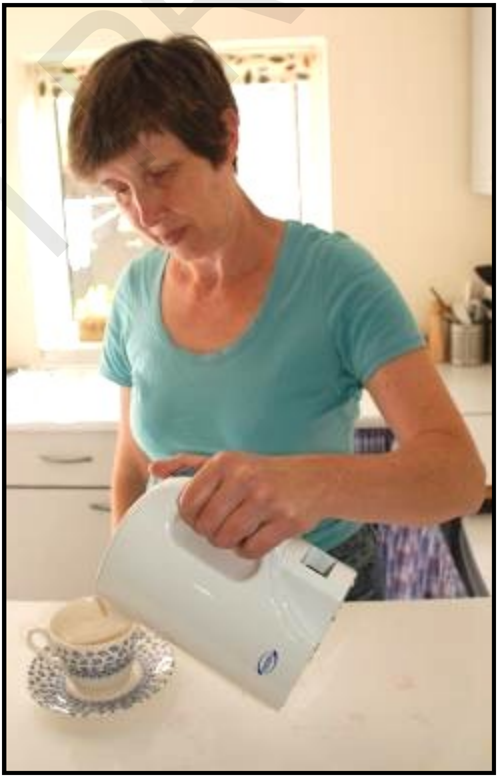

Touch knuckles to ruler

- Sit at a table with your forearm supported but your wrist over the edge of the table - hand facing down
- Place a ruler on the back of your forearm and secure with tape or your other arm - your therapist will advise you about this
- Lift your hand to try to touch your knuckles to the ruler
- Lower your hand after as slowly as possible

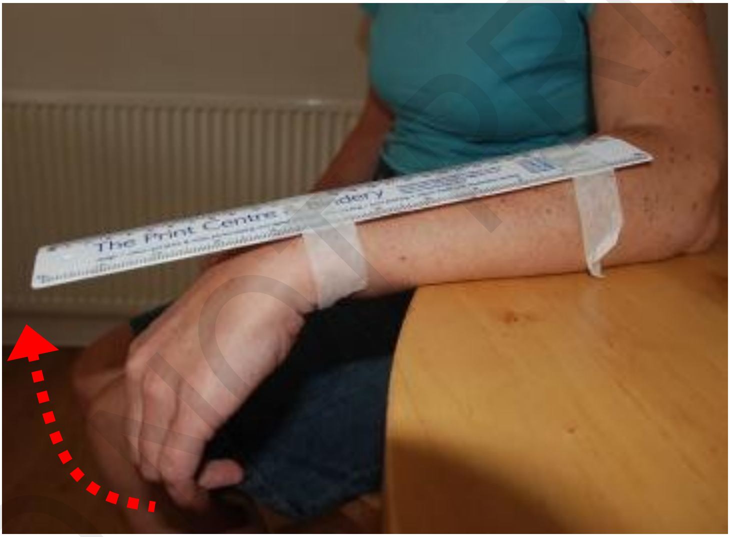

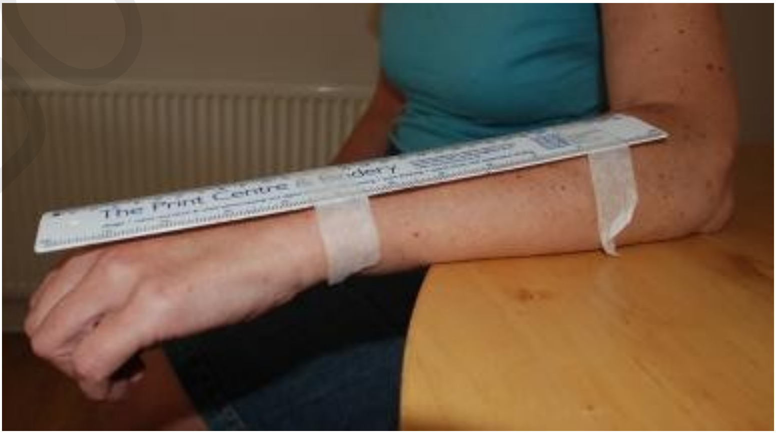

DO NOT PRINT

Lift hand to touch straw

- Sit with your forearm resting on a table in front of you and your hand facing down.
- Place a flexible straw on the top of your forearm so that the bend of the straw is over your wrist.
- Secure the straw with tape or your other arm—your therapist will advise you about this
- Practice lifting your hand to touch the tip of the straw then relaxing your hand back down again as slowly as possible.

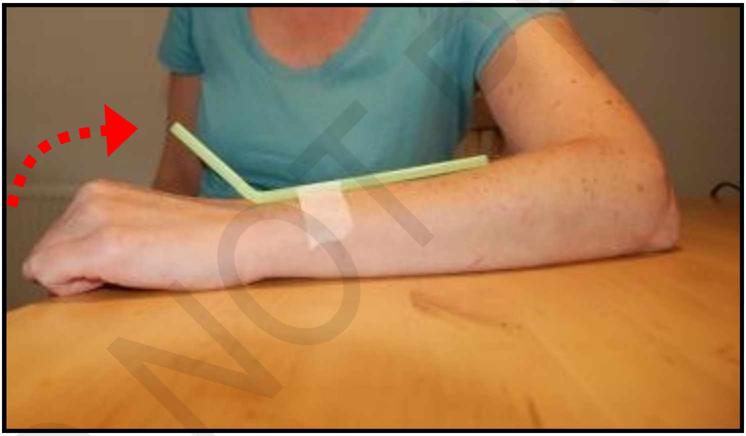

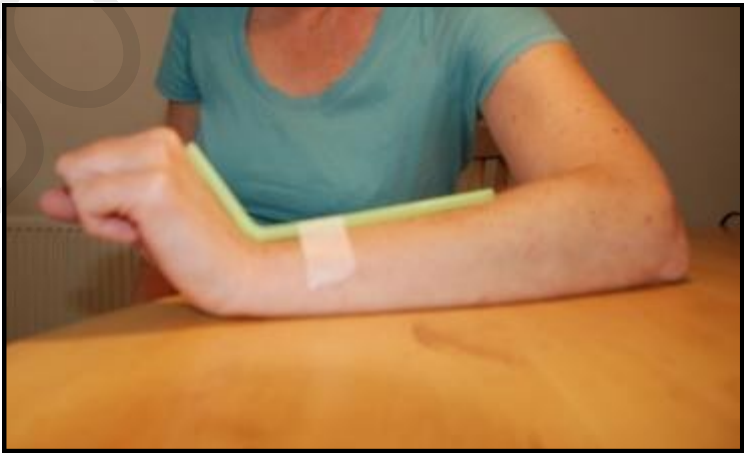

DO NOT PRINT

5.a.3

Bend and straighten wrist to touch 'target's

- Sit at a table with a weight, for example a bag of rice or pasta, on your forearm.
- Place 2 'target's either side of your hand and place an object in your hand, for example, a tumbler
- Practice bending your wrist backwards and forwards to touch the 'target's
- Try to keep your forearm still throughout.

DO NOT PRINT

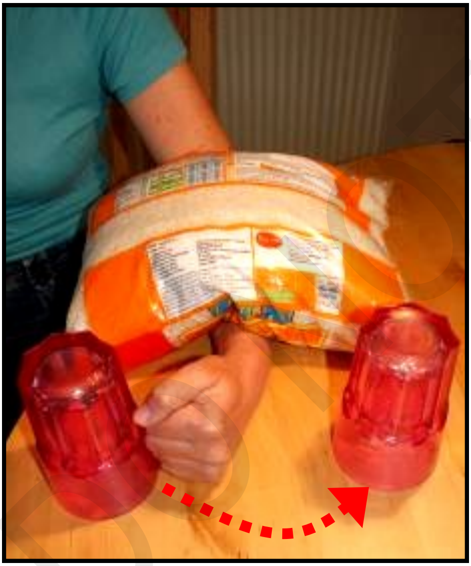

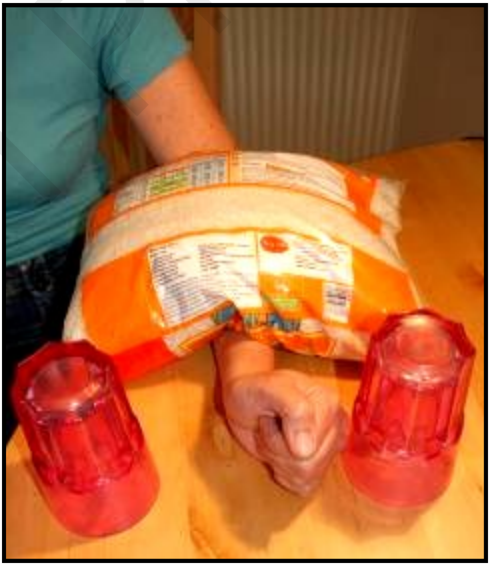

Draw curved lines with pen

- Sit at a table with your forearm supported and a piece of paper in front of you
- Draw curved lines on the paper by bending and straightening your wrist
- Try to keep your forearm still throughout.
- You may need to use your other arm to stabilise the paper

DO NOT PRINT

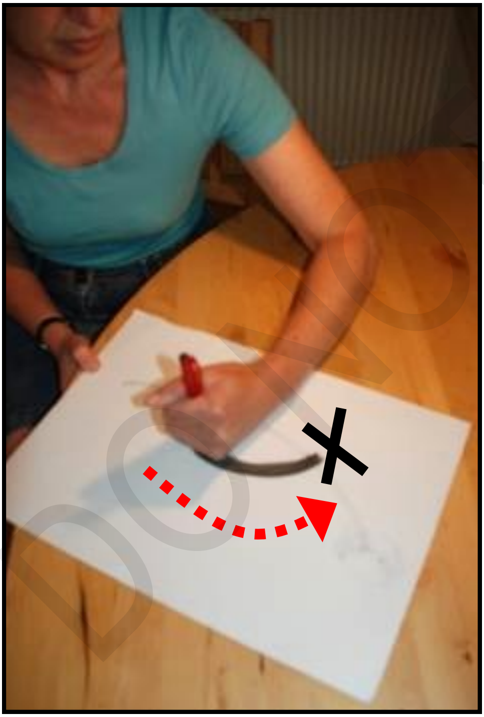

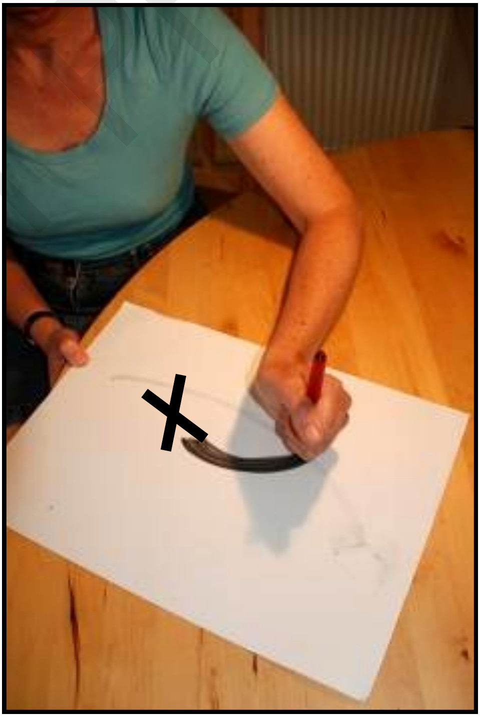

5.a.5

Knock on wall with knuckles

- Sit or stand with your forearm supported on a table or worktop with a wall or another vertical surface next to you
- Straighten your wrist to touch the back of your hand to touch the wall
- Try to keep your forearm still throughout.

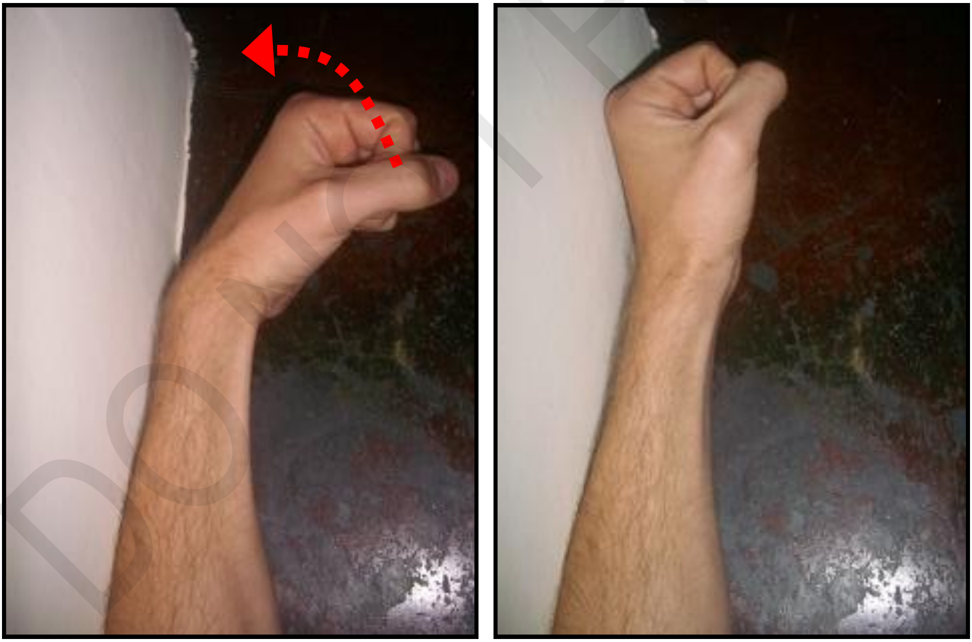

DO NOT PRINT

Roll ball along table between hands

- Sit at a table with both forearms supported and parallel - your therapist may provide you with a piece of paper with parallel lines on for you to position your forearms between
- Keeping your forearms still practice rolling a ball between your hands

DO NOT PRINT

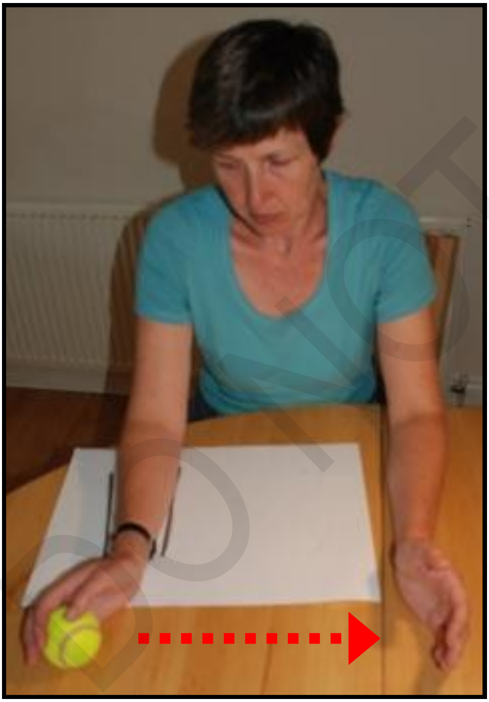

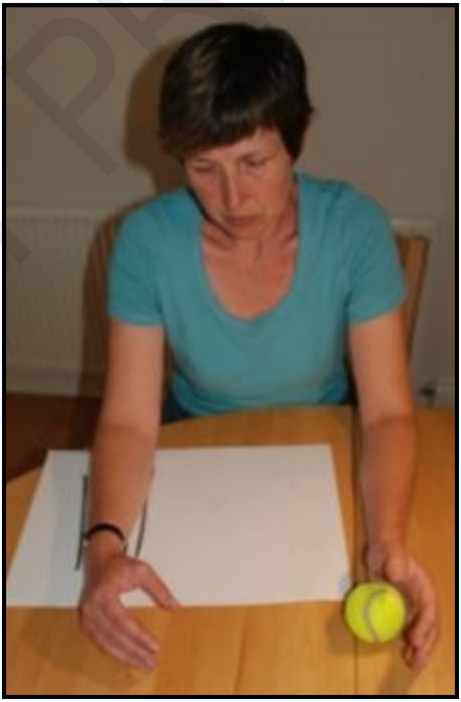

Knock ball against wall on table with back of hand

- Sit at a table with your forearm supported and a wall or other vertical surface, such as a box, next to you.
- Practice knocking a ball against the wall / box with the back of your hand
- Try to keep your forearm as still as possible

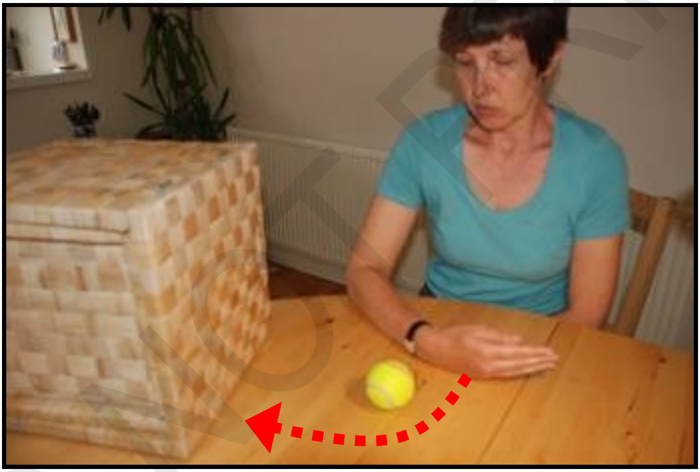

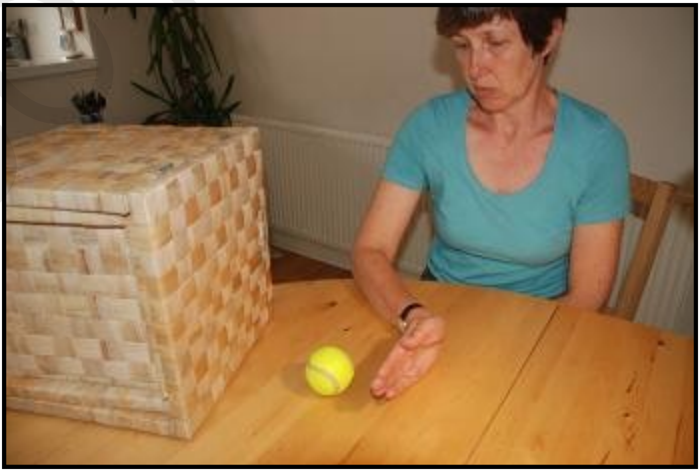

DO NOT PRINT

5.b.1

Touch thumb to ruler

- Sit at a table with the side of your forearm supported but your wrist over the edge of the table - thumb facing up
- Place a ruler on your forearm and secure with tape or your other arm - your therapist will advise you about this
- Lift your hand to try to touch your thumb to the ruler
- Lower your hand after as slowly as possible

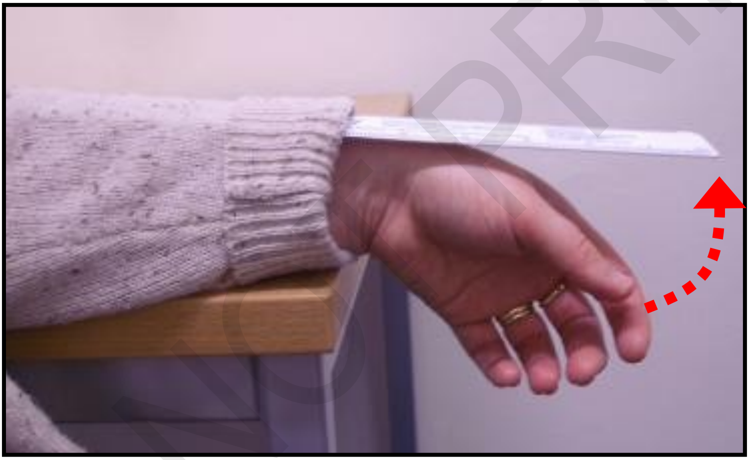

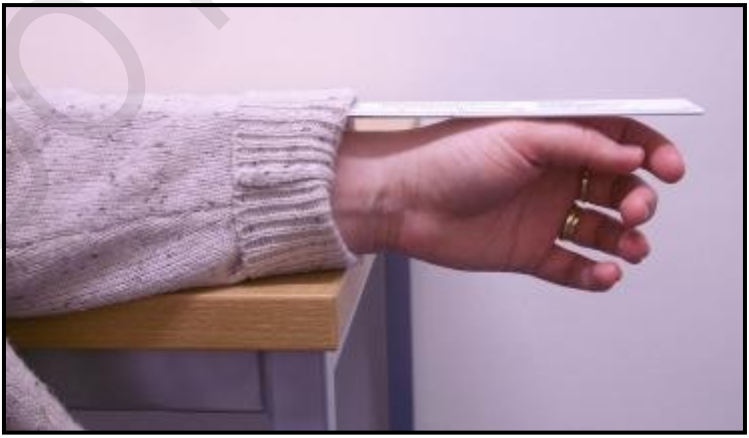

DO NOT PRINT

Lift tumbler off table

- Sit at a table with your forearm supported
- Place an object in your hand, for example a tumbler
- Keeping your forearm in contact with the table, lift the object as high off the table as possible
- Lower back down to table as slowly as possible

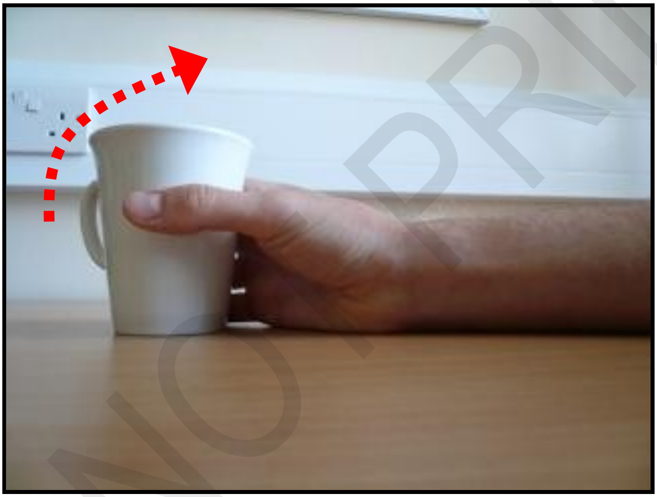

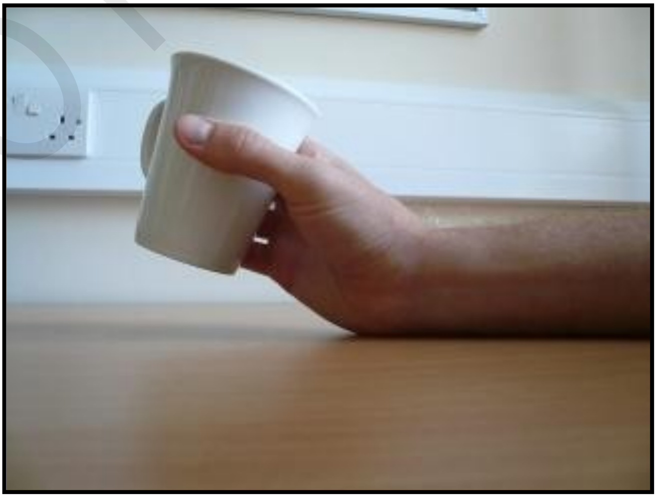

DO NOT PRINT

5.b.3

Screw on and unscrew jar lid

- Sit at a table with a jar in front of you
- The jar may need to be loosened by your therapist or carer
- Practice unscrewing and screwing jar lid with your weaker hand
- Stabilise the jar with your other hand

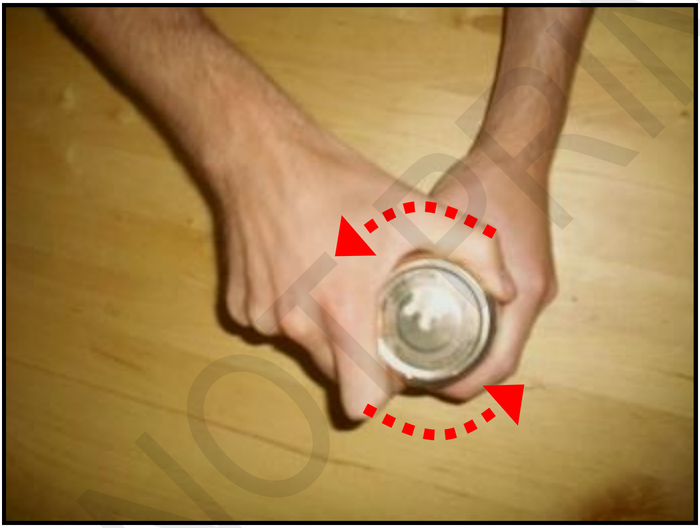

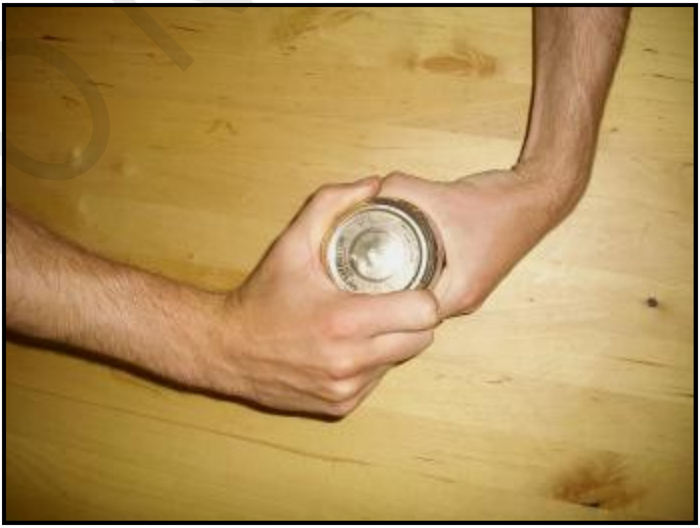

DO NOT PRINT

Lift tumbler over object

- Sit at a table
- Your therapist will provide you with a piece of paper with parallel lines on for you to position your forearm between
- Place an object, for example a tumbler, in your hand.
- Start with your wrist bent then extend to move the tumbler to the 'target' position

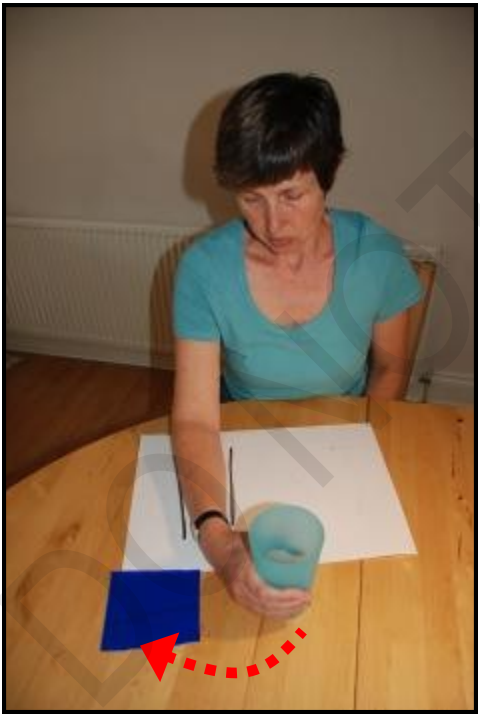

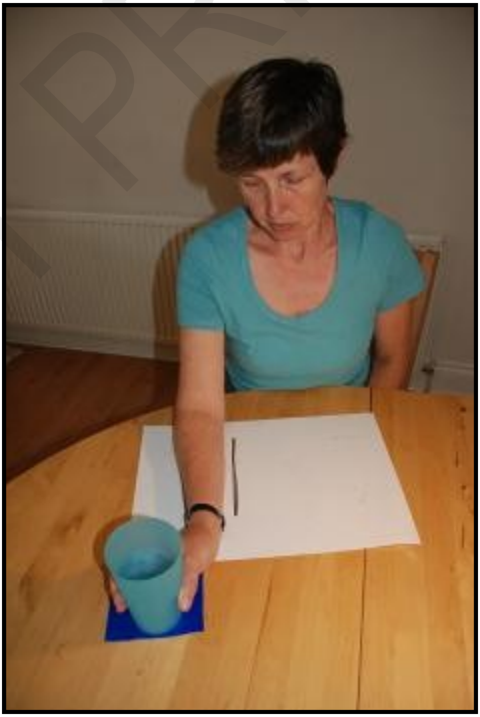

DO NOT PRINT

Cut up food with knife and fork

- Sit at a table
- Practice using knife and fork to cut up food
- Your therapist may provide you with different cutlery to assist with the task if needed

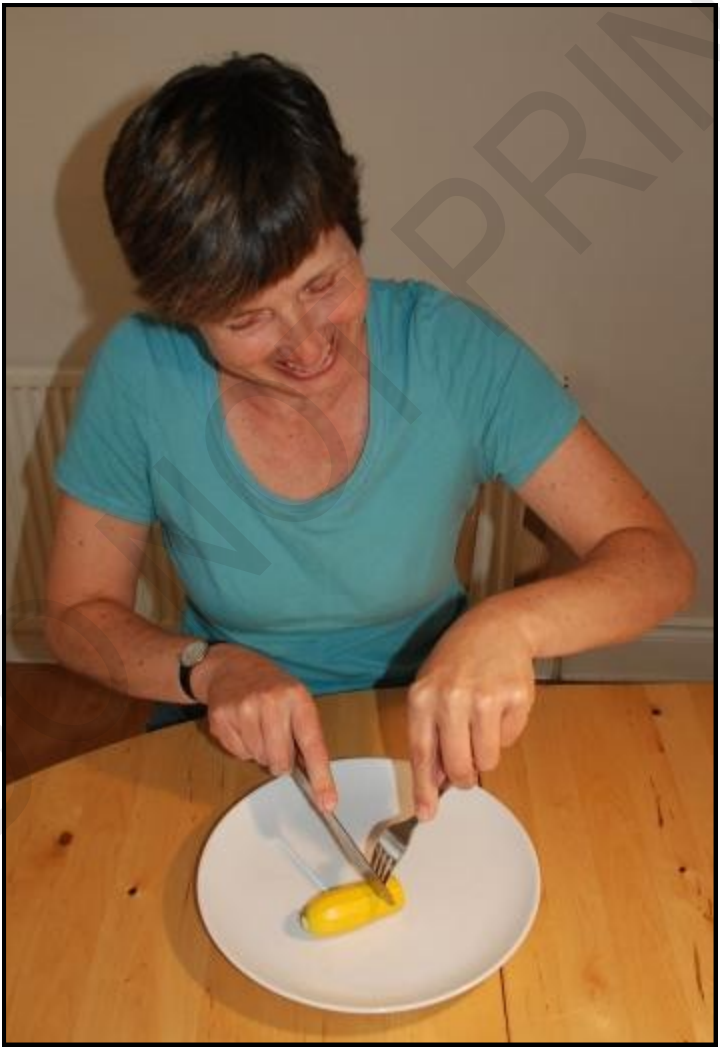

DO NOT PRINT

5.d.2

Lift cup onto saucer

- Sit at a table with your forearm supported on the table and a cup in your hand—this is the ‘start’ position
- Position a saucer in front of you
- Practice lifting the cup onto the saucer then returning to the ‘start’ position
- Move the saucer between each attempt so that you practice reaching in different directions

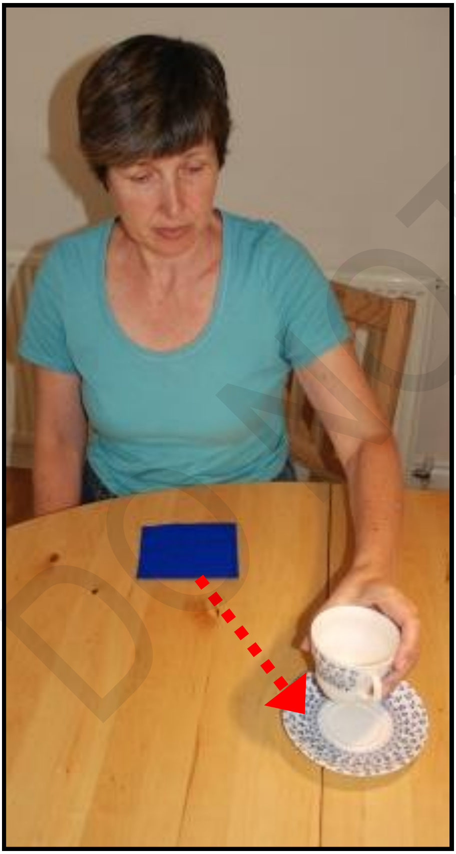
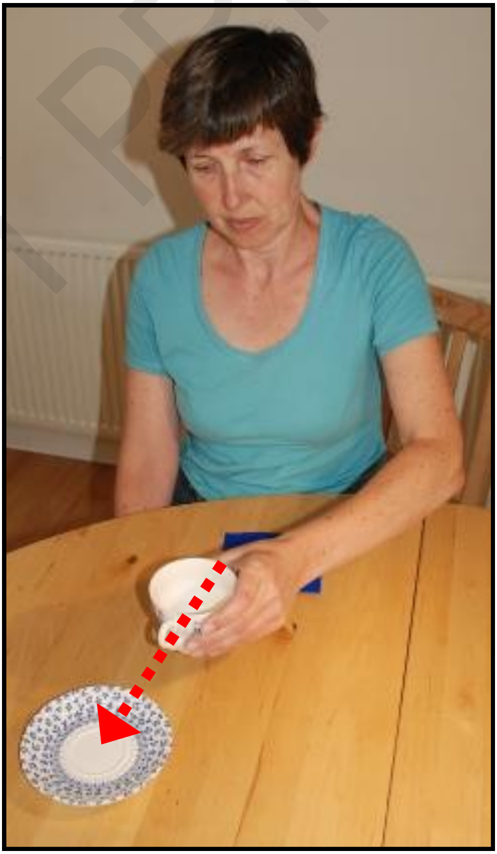

DO NOT PRINT

Take cup to mouth

- Sit at a table
- Place a tumbler in your hand
- Practice lifting the tumbler to your mouth
- Your therapist will advise you whether to practice with or without water
- Try to stay sat upright with your back against the chair throughout

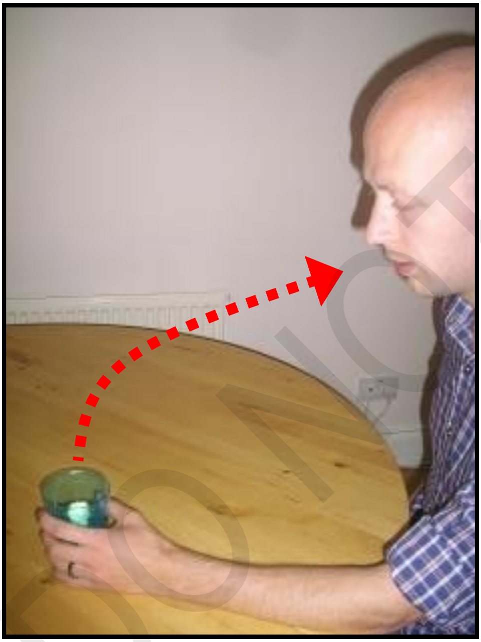

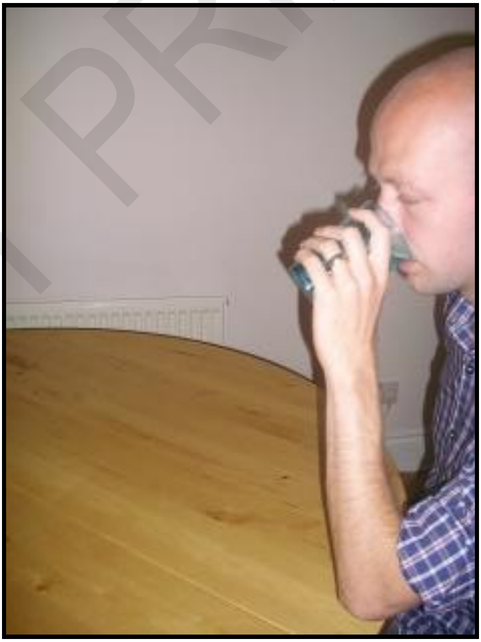

DO NOT PRINT

Bounce tennis ball on racquet

- Stand with plenty of space around you
- Hold a tennis racquet in your weaker hand
- Practice bouncing a tennis ball on the racquet
- Count how many bounces you do

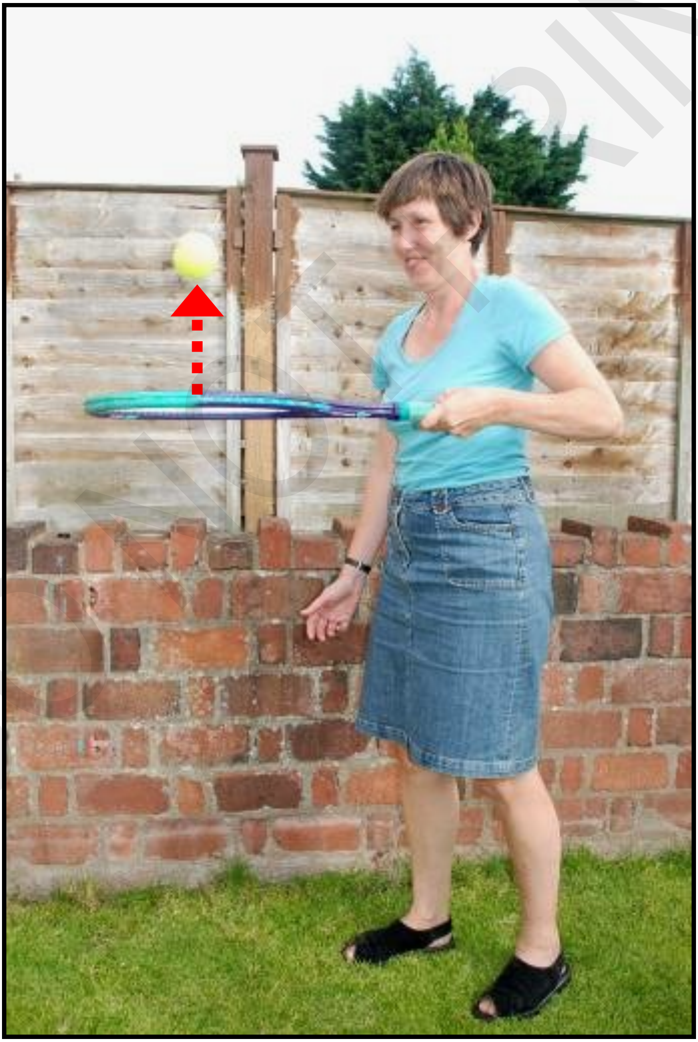

DO NOT PRINT

Bounce ball

- Stand with plenty of space around you
- Practice bouncing a football / basketball / tennis ball with your weaker hand
- Count how many bounces you do

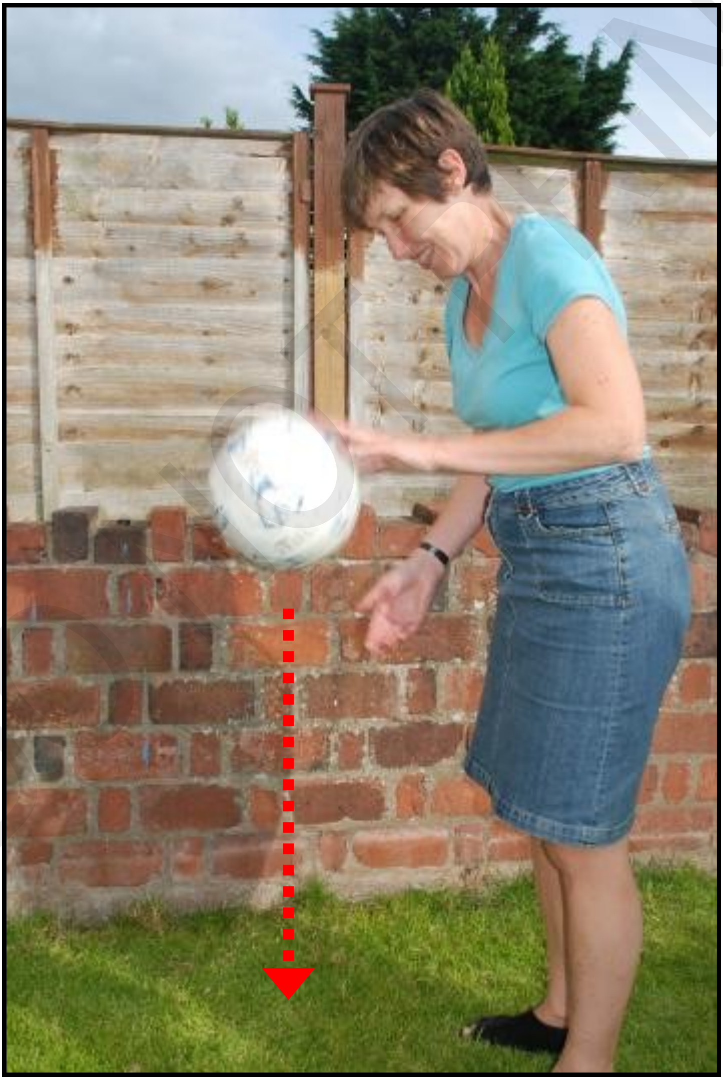

DO NOT PRINT

5.d.6

Reach for chair arms and push up to stand up and sit down

- Sit in a chair with arms and place your hands on your lap
- Reach for the chair arms with both hands
- Stand up using your hands to assist
- When sitting down, reach back for chair arms and try to sit down slowly

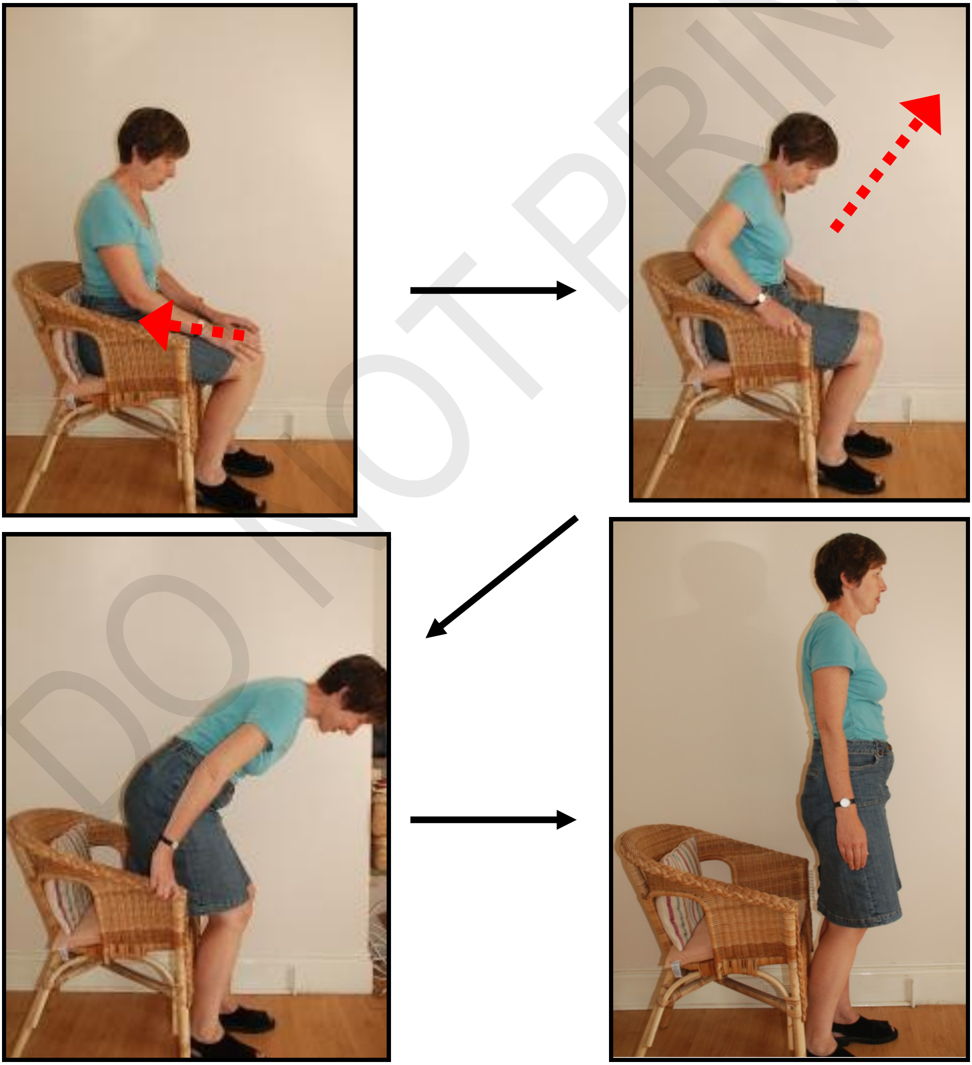

DO NOT PRINT

6.a.1

Straighten fingers to touch ruler

- Sit at a table with your forearm supported and hand facing down but with your fingers over the edge of the table
- Place a ruler on the back of your forearm and secure with tape or your other arm—your therapist will advise you about this
- Lift your fingers to try to touch your fingertips to the ruler
- Lower your fingers after as slowly as possible

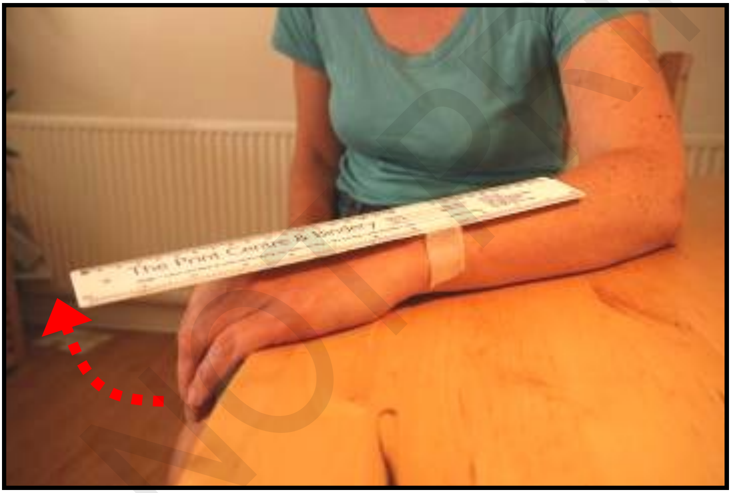

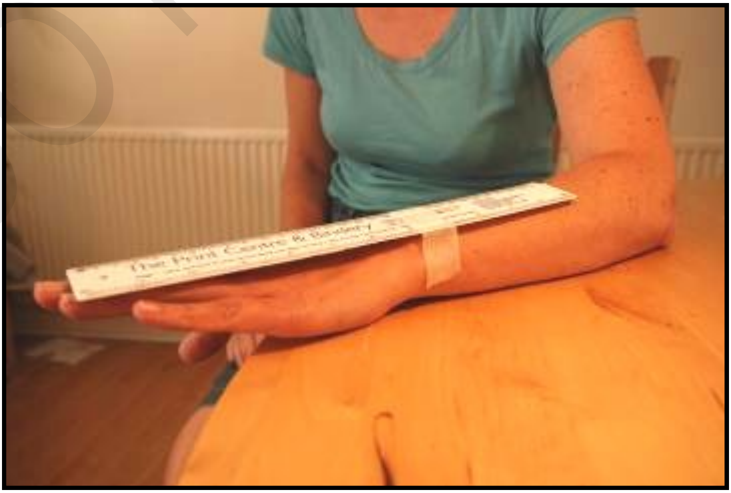

DO NOT PRINT

Touch fingers to straw

- Sit with your forearm resting on a table in front of you and your hand facing down.
- Place a flexible straw on the top of your forearm so that the bend of the straw is over your knuckles.
- Secure the straw with tape or your other arm—your therapist will advise you about this
- Practice lifting your fingers to touch the straw then lowering your fingers back down again as slowly as possible.

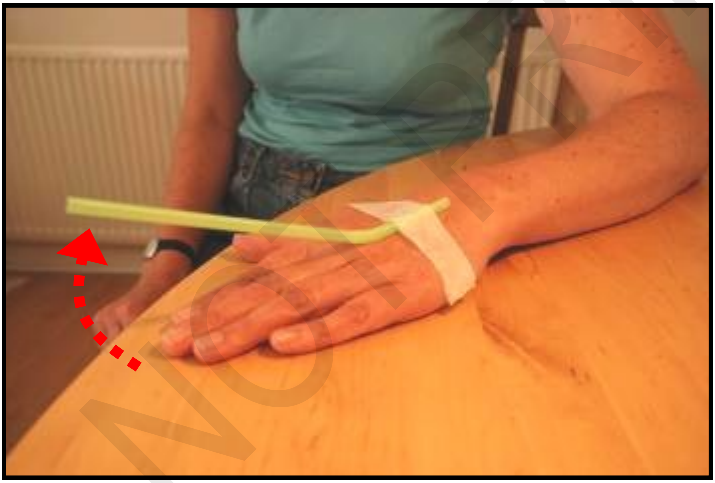

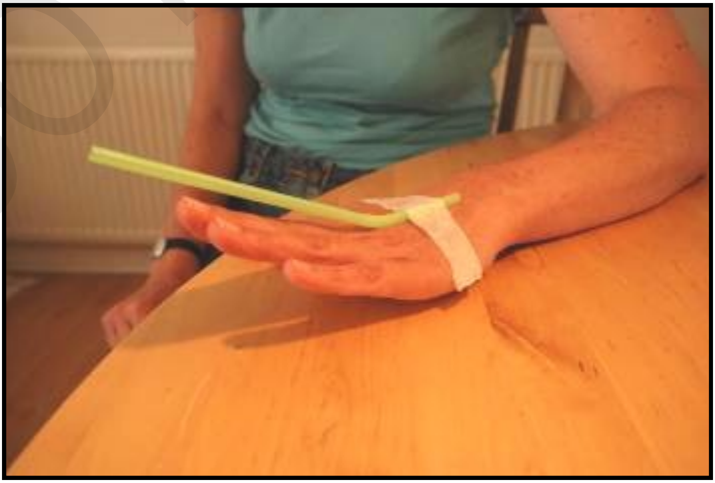

DO NOT PRINT

6.a.3

Lift finger up along ruler

- Sit with your hand face down on a table
- Place a ruler vertically next to your hand - your therapist will advise you about how this can be secured by taping it to a glass
- Practice lifting one finger at a time to see how high you are able to lift your finger up the ruler
- Aim to increase height able to lift each finger

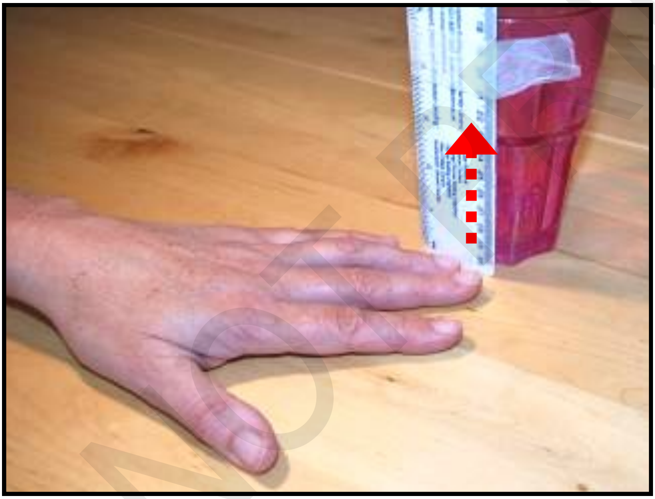

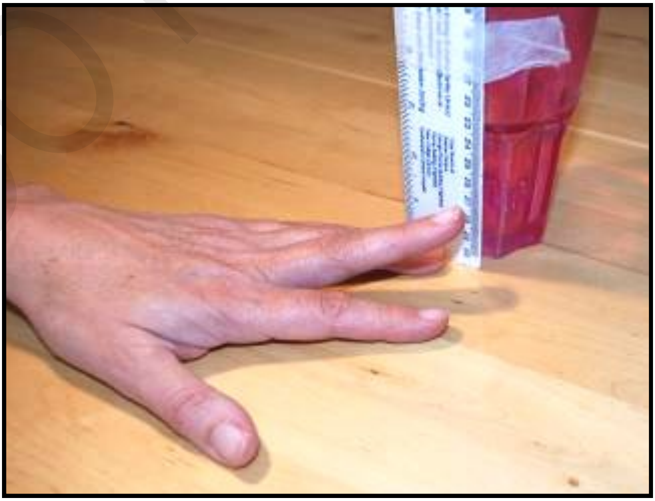

DO NOT PRINT

Straighten and bend fingers using 'target's

- Sit at a table with an object in your hand, for example, a tumbler.
- Keeping your forearm, wrist and thumb still, practice straightening your fingers to touch a 'target' object with your finger nails.

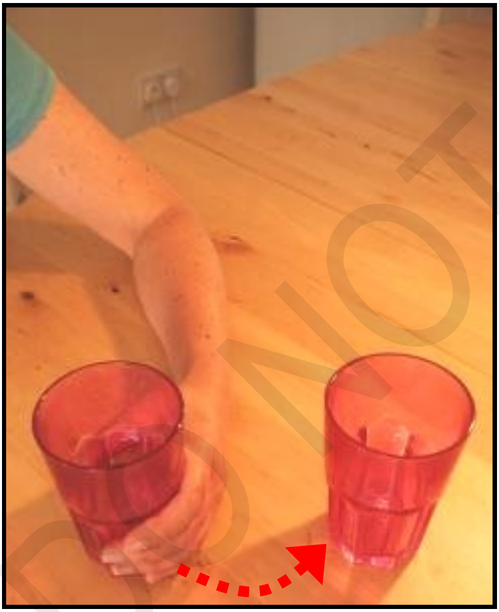

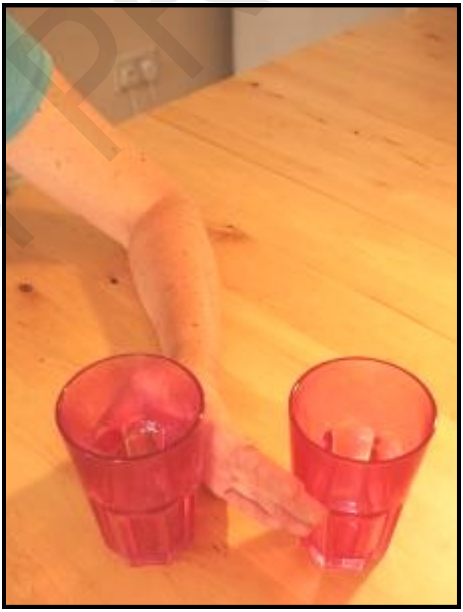

DO NOT PRINT

6.a.5

Knock object over with finger extension.

- Sit at a table with your forearm supported
- Keep your forearm and wrist still
- Extend your fingers to knock an object over

DO NOT PRINT

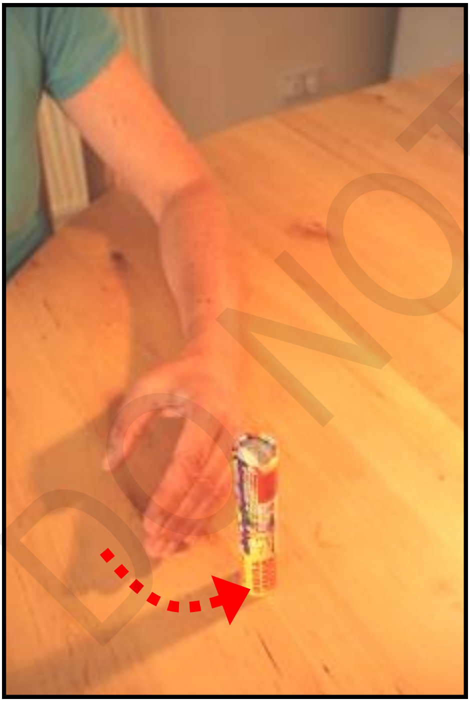

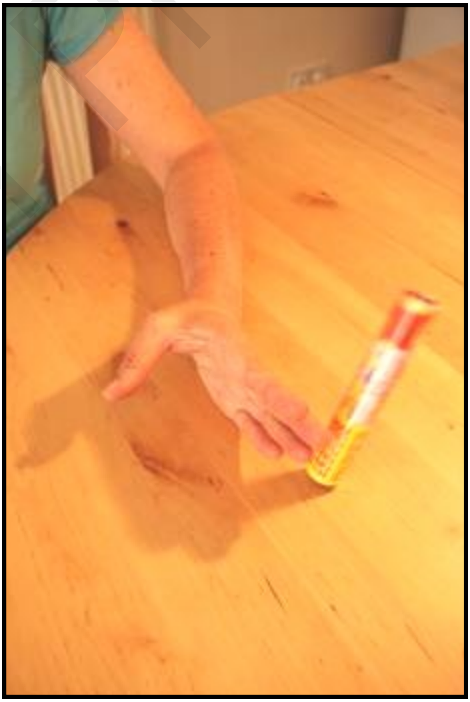

6.a.6

Straighten fingers to touch peg

- Sit at a table with your forearm supported and your little finger in contact with the table
- Attach a peg / ice lolly stick to the back of your finger - your ther-apist will advise you how to do this and whether to do one at a time or all 4 at the same time
- Straighten your fingers to touch the peg / ice lolly stick with your finger nail.

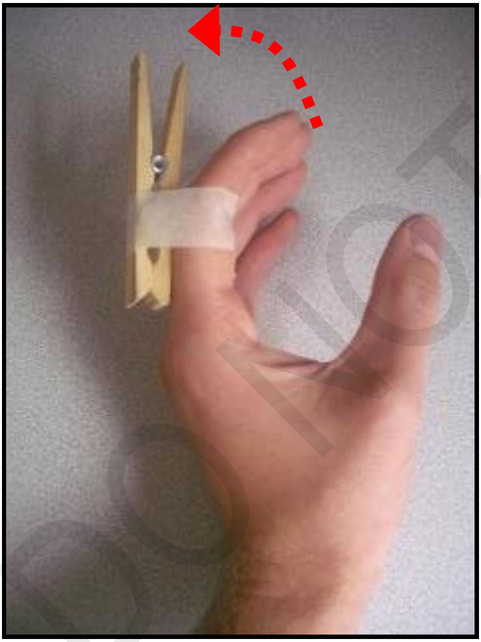

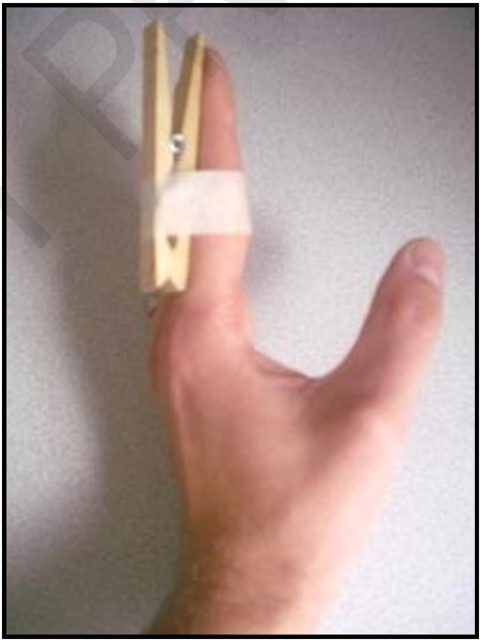

DO NOT PRINT

6.b.1

Tap thumb on tumbler

- Sit at a table with your forearm supported
- Hold an object, for example a tumbler, in your hand
- Move your thumb as far away from the object as possible then tap your thumb on the object

DO NOT PRINT

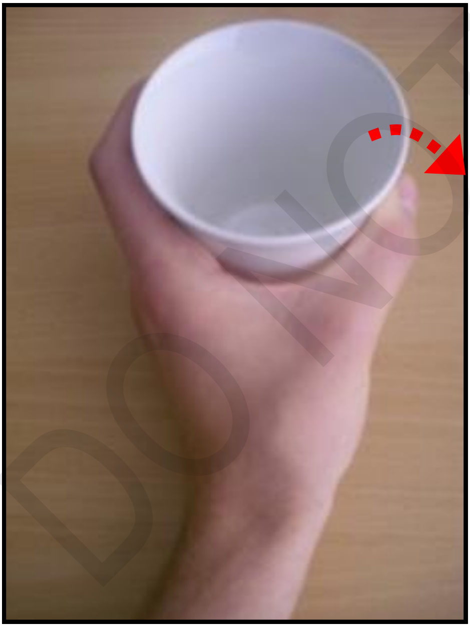

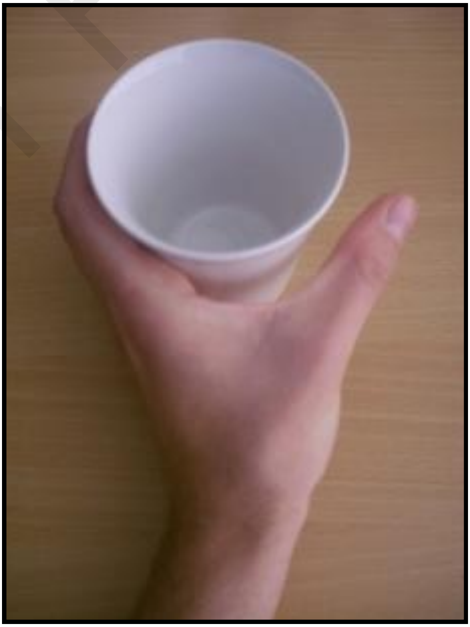

6.b.2

Knock object over with thumb

- Sit at a table with your hand on the edge and your thumb supported on the table
- Move your thumb away from the edge to knock an object over
- Aim to move the object further away to increase the thumb movement needed

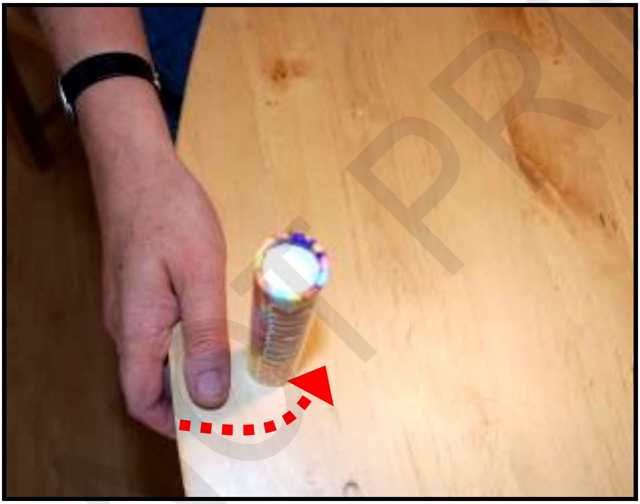

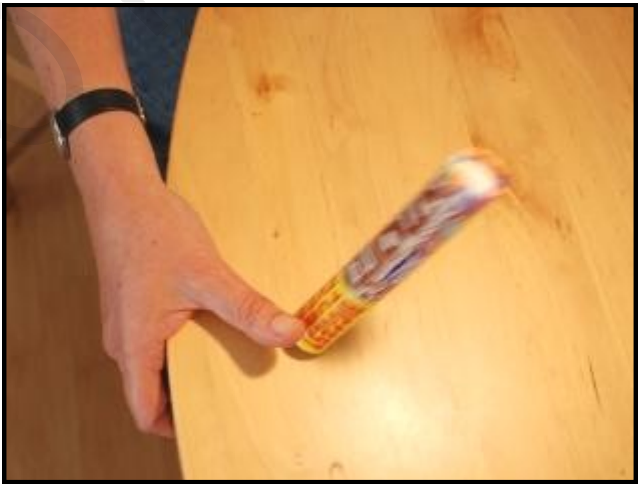

DO NOT PRINT

6.b.3

Slide thumb on table

- Sit at a table with your hand on the edge and your thumb supported on the table
- Place a piece of paper under your thumb and mark your starting position
- You may need to stabilise the paper with your other hand
- Move your thumb away from the edge as far as possible and mark again

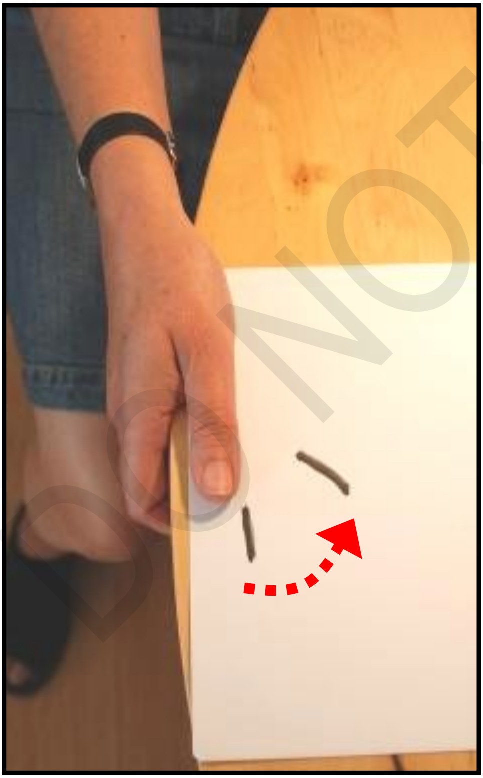

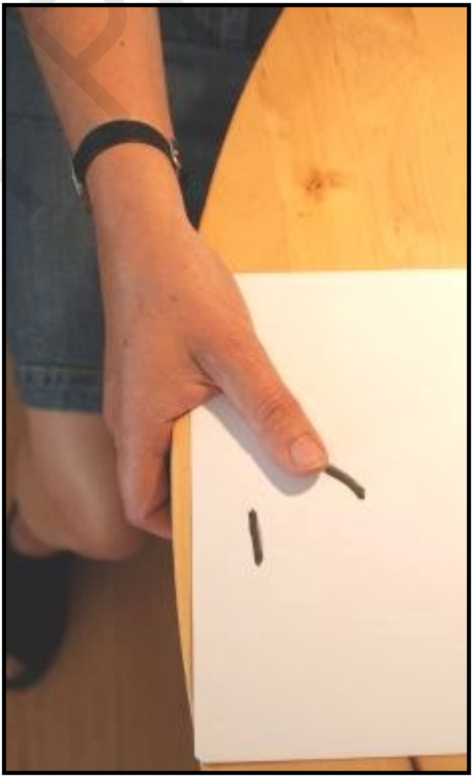

DO NOT PRINT

Slide thumb on a ruler

- Sit at a table with your forearm supported and a ruler balanced between 2 objects - your therapist will show you how to set this up
- Position your hand vertically with your thumb resting on top of the ruler.
- Practice sliding your thumb along the ruler away from your index finger.
- Ensure that only your thumb moves - try to keep your forearm and wrist still.

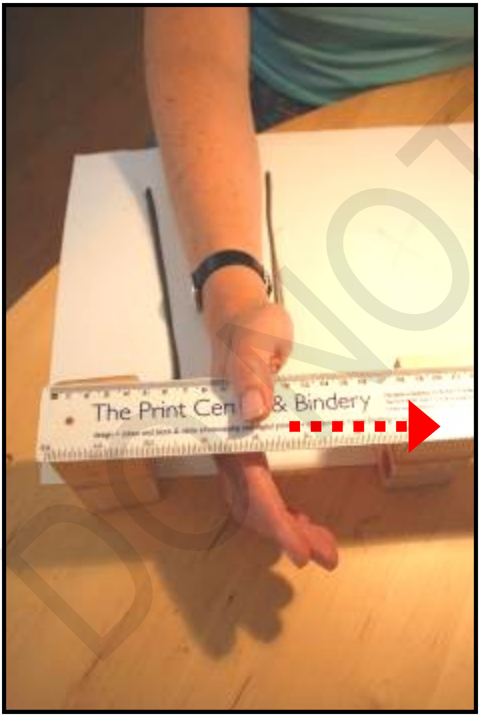

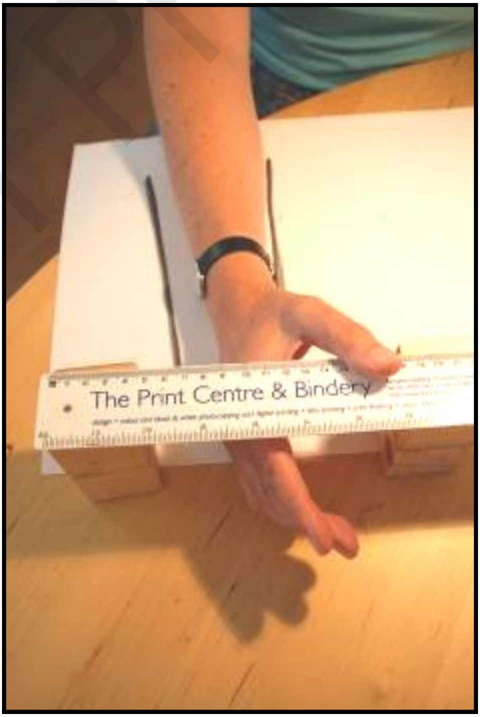

DO NOT PRINT

Slide thumb along a stick with hand around cup

- Sit at a table
- Your therapist will provide you with a cylindrical object that has a stick attached to the top of it .
- Grasp the object and position your thumb and index finger on top of the stick.
- Practice moving your thumb away from the object to reach a 'target' line.
- Ensure that you don`t squeeze the object and only your thumb moves.

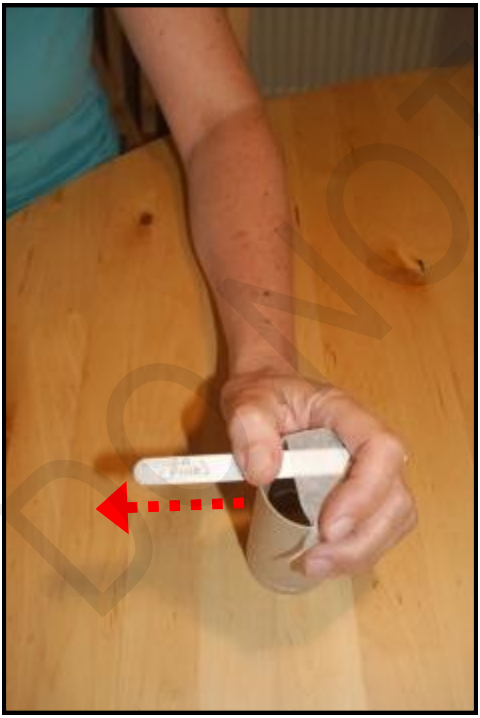

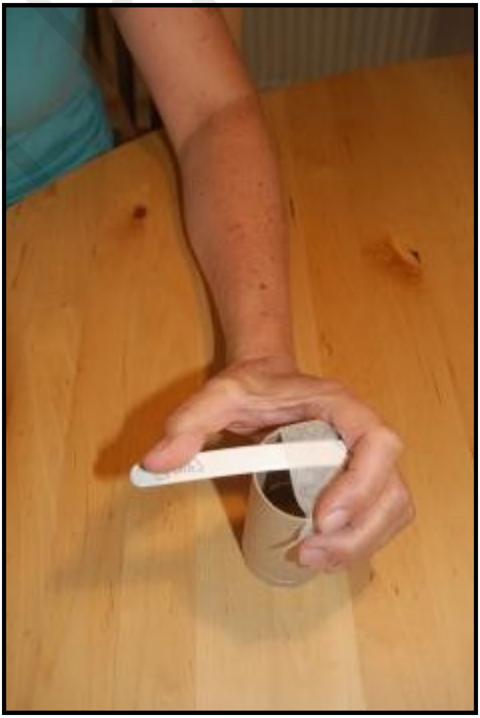

DO NOT PRINT

6.b.6

Slide thumb down a stick with hand around cup

- Sit in front of a table
- Your therapist will provide you with a cylindrical object that has a stick pushed through it.
- Grasp the object and position your thumb on top of the stick.
- Practice moving your thumb away from the object to reach a 'target' line.
- Ensure that you don`t squeeze the object and only your thumb moves.

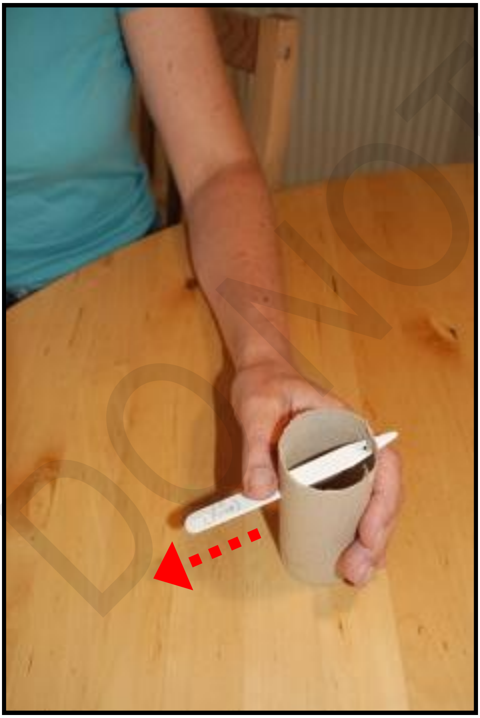

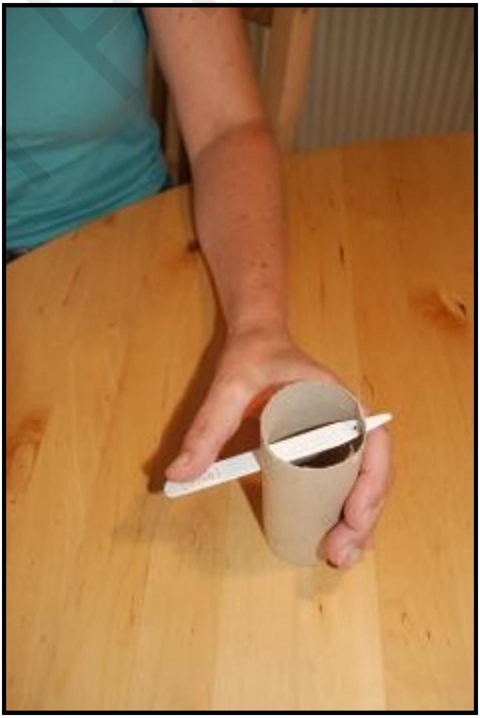

DO NOT PRINT

6.c.1

Squeeze and release tumbler

- Sit at a table with your forearm supported and an object, for example a tumbler, in your hand
- Squeeze the tumbler then release
- Try to move all of your fingers and thumb away from the tumbler without moving your forearm

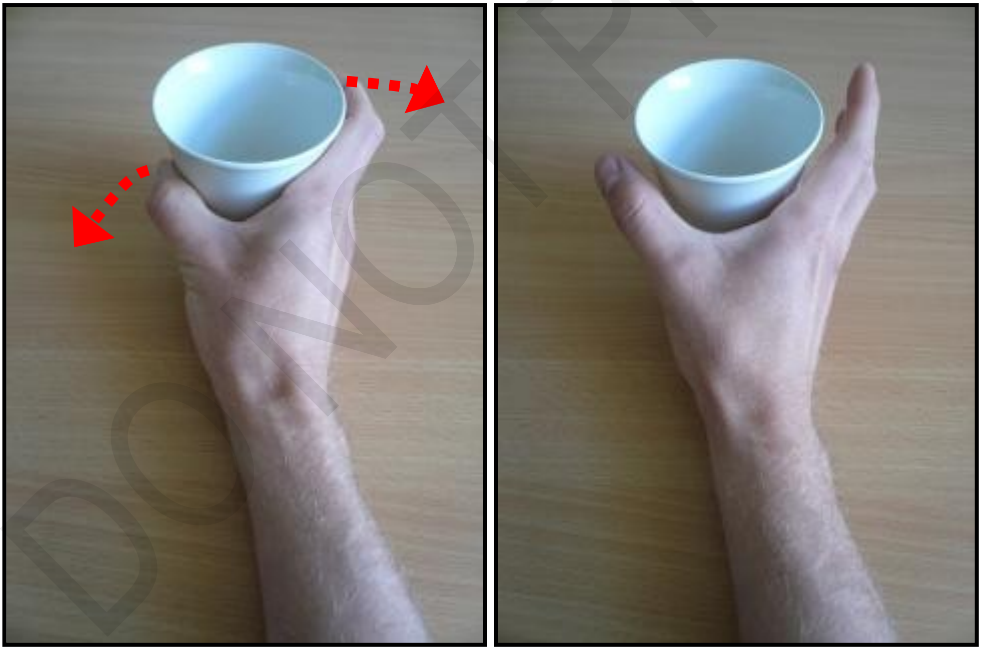

DO NOT PRINT

Grasp different sized objects

- Sit at a table with your forearm supported and a number of different sized objects in front of you
- Keep your forearm still and practice grasping and releasing the different objects

DO NOT PRINT

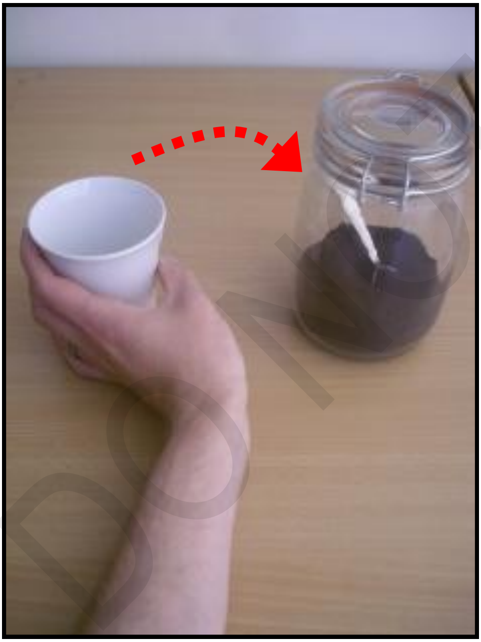

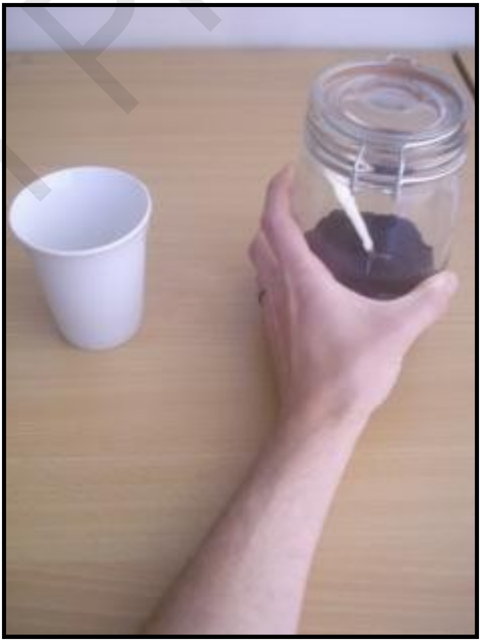

6.c.3

Mark measurements on ruler with thumb and index finger

- Sit at a table with a ruler lay horizontally in front of you
- Using your thumb and index finger, mark increments on a ruler, e.g. 1cm, then 2cm, then 3cm, or 1 inch, 2 inches, etc.

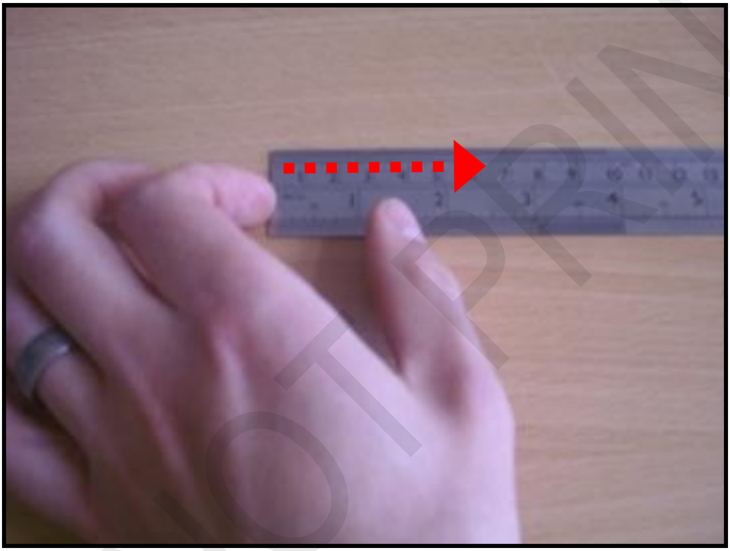

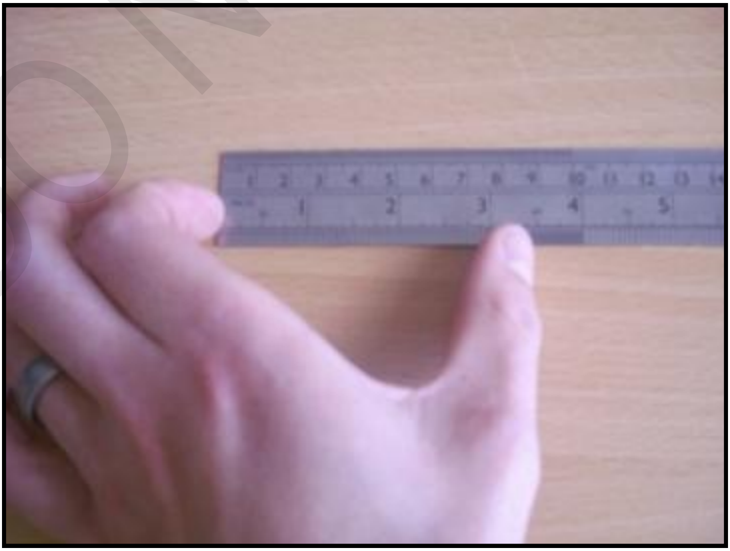

DO NOT PRINT

6.c.4

Drop different sized objects into bin

- Sit at a table with your forearm supported but your hand over the edge
- Place an object, for example a paper ball, in your hand
- Practice dropping the object by opening your fingers and thumb

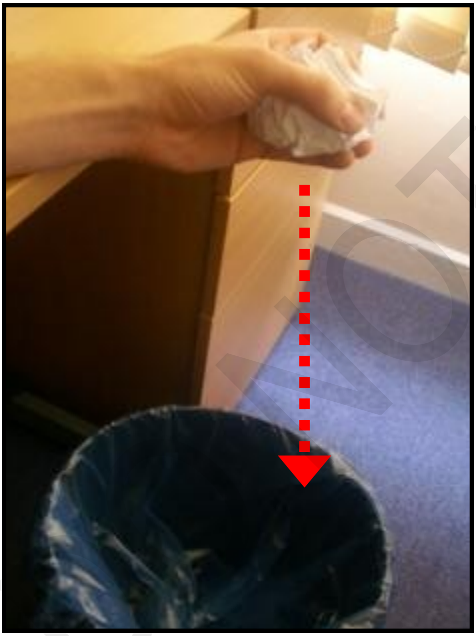

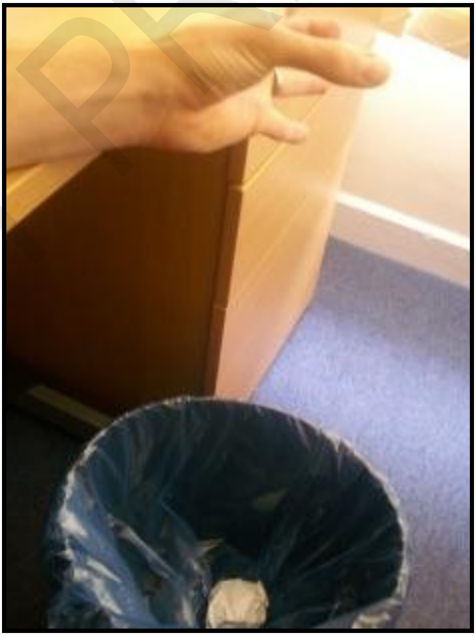

DO NOT PRINT

Drop wash rag

- Grasp wash rag and lift off table
- Drop back onto table by opening your hand

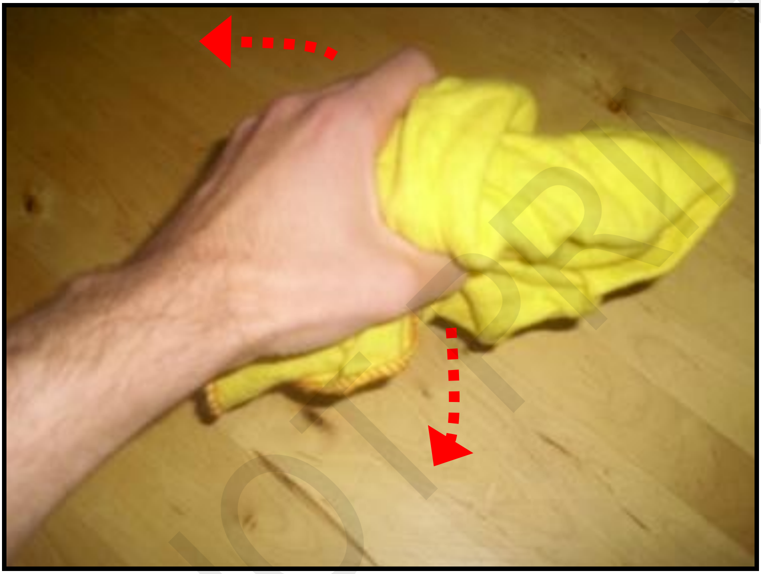

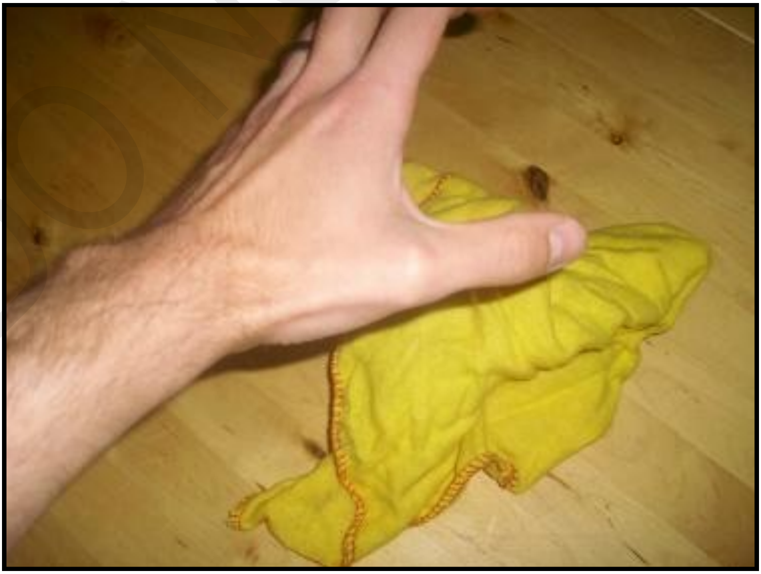

DO NOT PRINT

Move object around

- Sitting or standing
- Practice grasping different objects, for example tumblers, mugs, books, etc, and moving them to different positions
- Vary the height and position you reach for and release the objects - your therapist will be able to guide you on this.

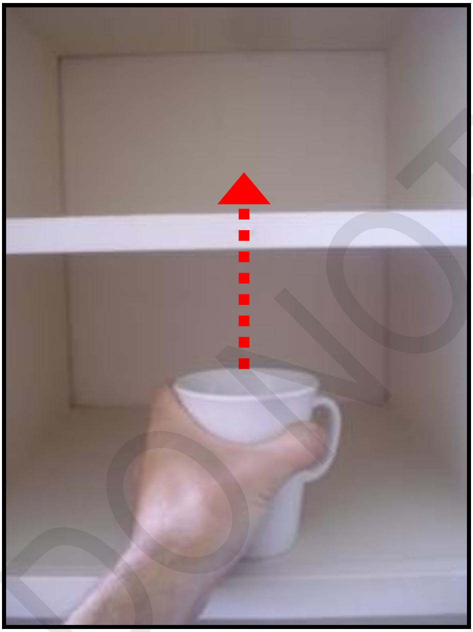

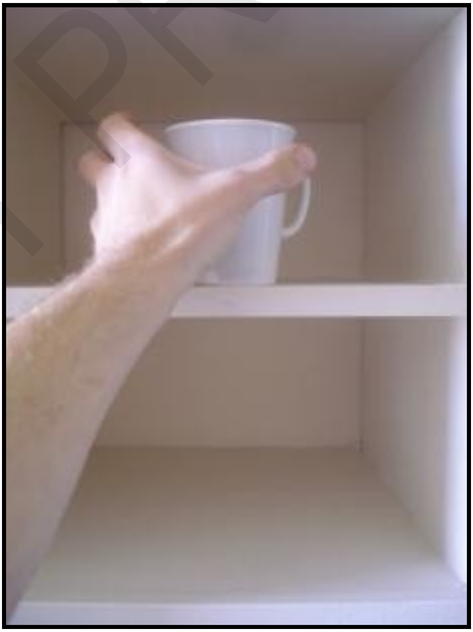

DO NOT PRINT

6.d.3

Drop ball on table and catch again

- Sit at table
- Practice dropping a ball onto the table
- Catch the ball as it bounces if you are able

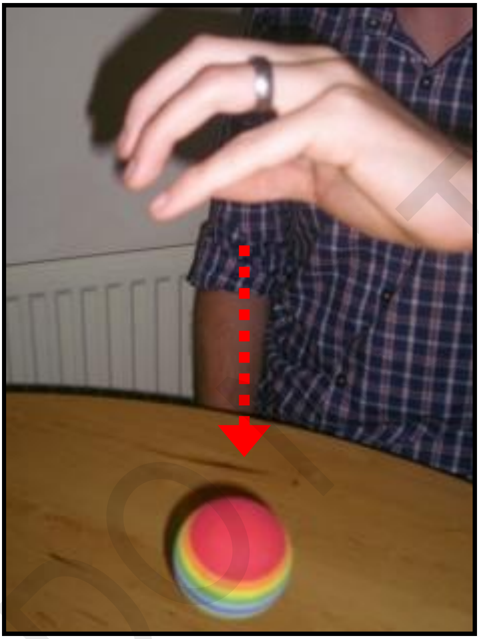

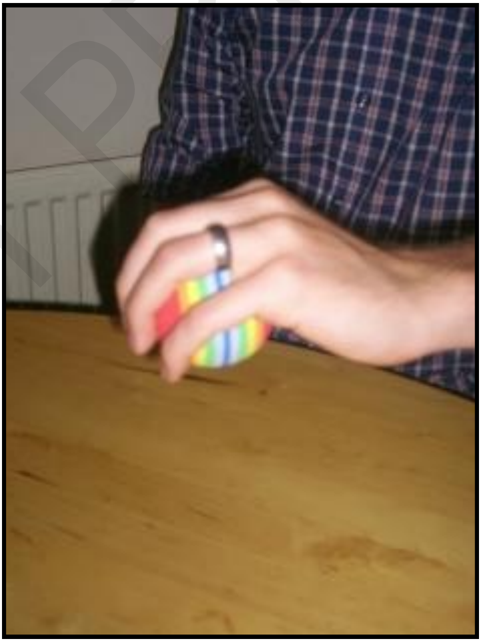

DO NOT PRINT

7.a.1

Grip with ring and little fingers

- Sit with a small piece of foam between the palm of your hand and your ring and little fingers
- Practice squeezing your ring and little fingers to squash the foam into your palm

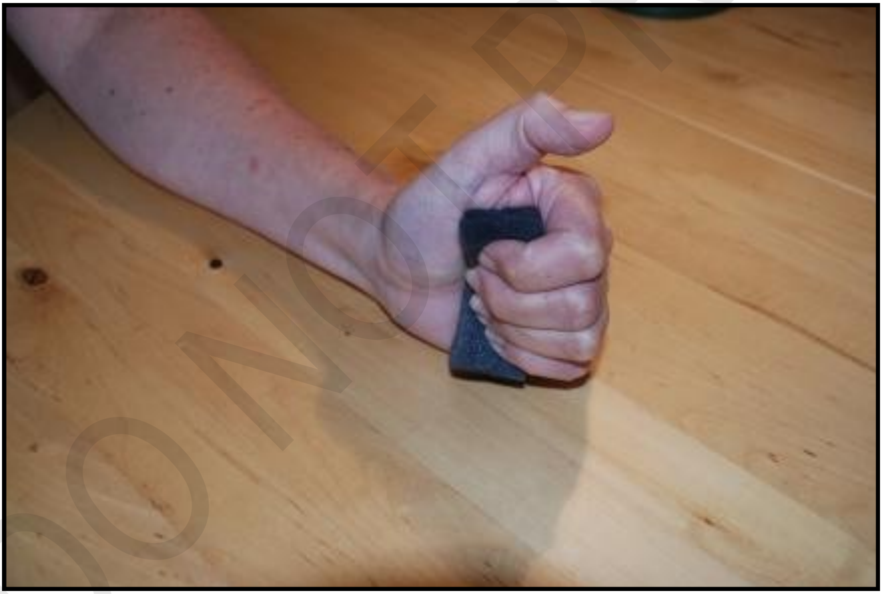

DO NOT PRINT

7.b.1

Squeeze clothes peg

- Grip the end of a peg between your index finger and thumb
- Squeeze to open the peg
- You may need to use your other hand to stabilise the peg initially

DO NOT PRINT

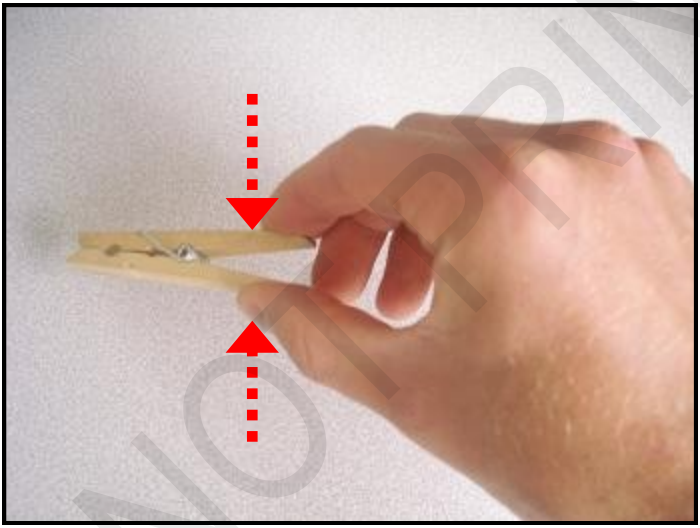

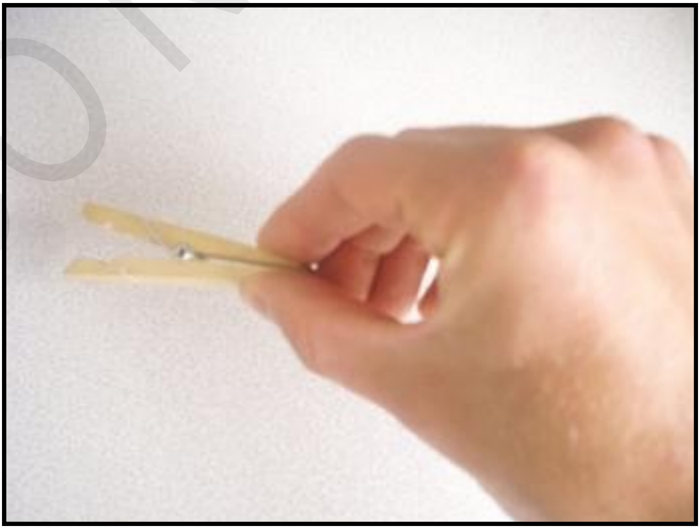

7.b.2

Lower peg from horizontal to vertical

- Sit at a table and hold the end of a peg between your index finger and thumb.
- Hold the peg horizontally.
- Practice reducing the pressure on the peg to allow the peg to lower slowly to a vertical position.
- Ensure that you don't let go of the peg.

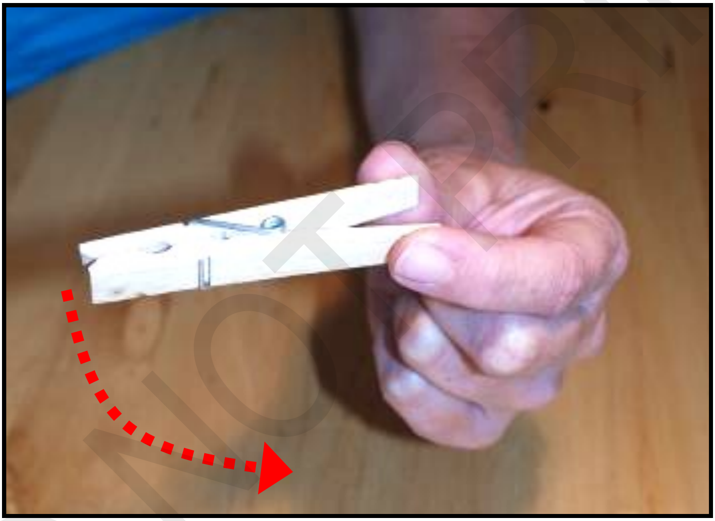

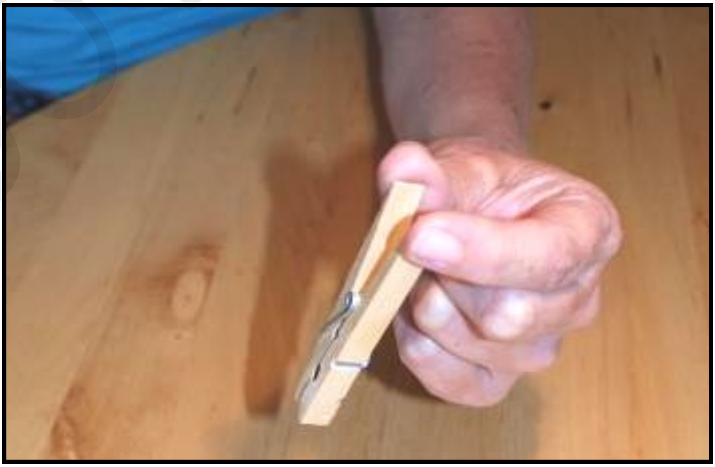

DO NOT PRINT

7.b.3

Lift a sealed bottle and straw

- Sit with a sealed, thin plastic bottle and straw on a table in front of you.
- Fill the plastic bottle most of the way with some coloured liquid and mark a line on the straw - your therapist will assist you to set this up
- Practice lifting up the bottle without letting the fluid raise above the marked line on the straw.

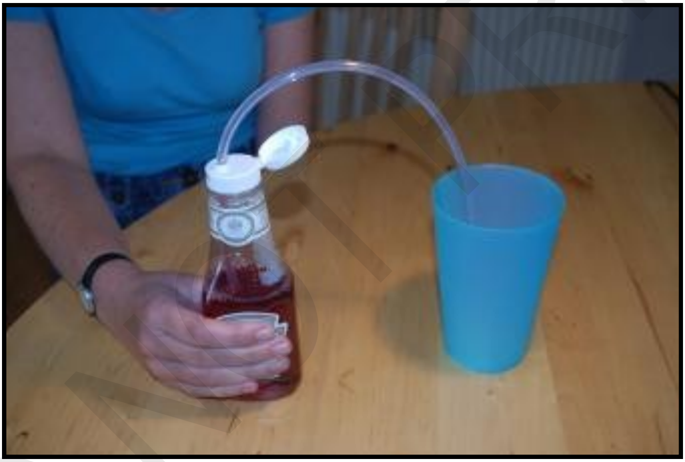

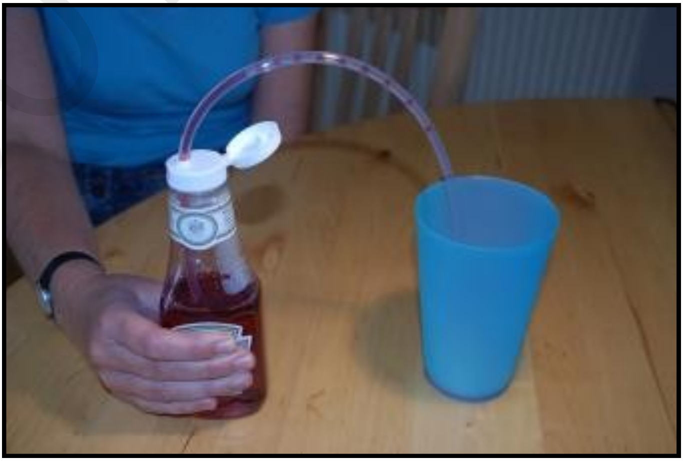

DO NOT PRINT

Pick up small object then drop

- Sitting or standing in front of table
- Pick up a small object between your index finger and thumb
- Drop the object onto the table by releasing the pinch grip

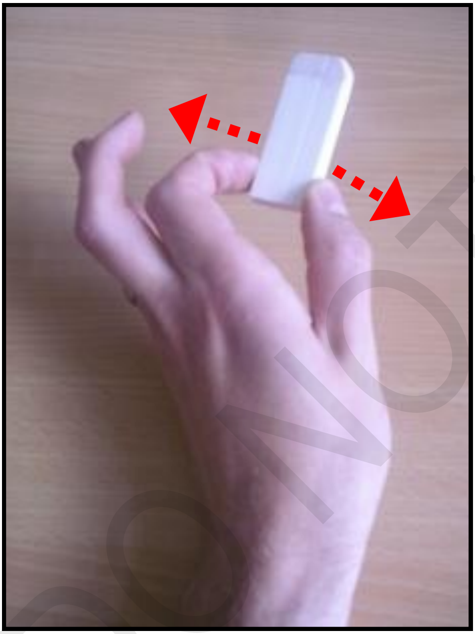

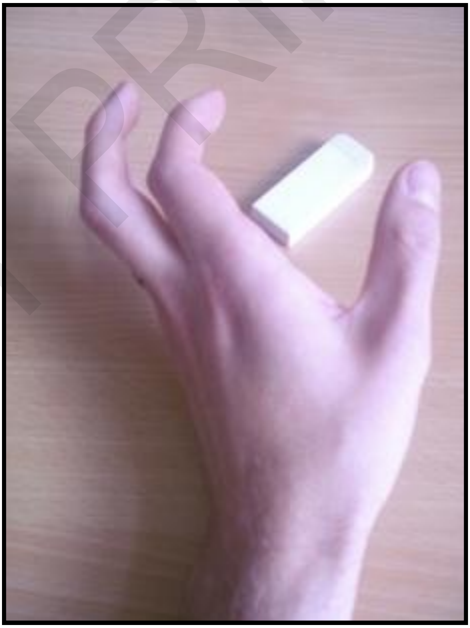

DO NOT PRINT

7.b.5

Pick up small object then replace

- Sitting or standing in front of table
- Pick up a small object between your index finger and thumb
- Replace the object without knocking over by releasing pinch grip

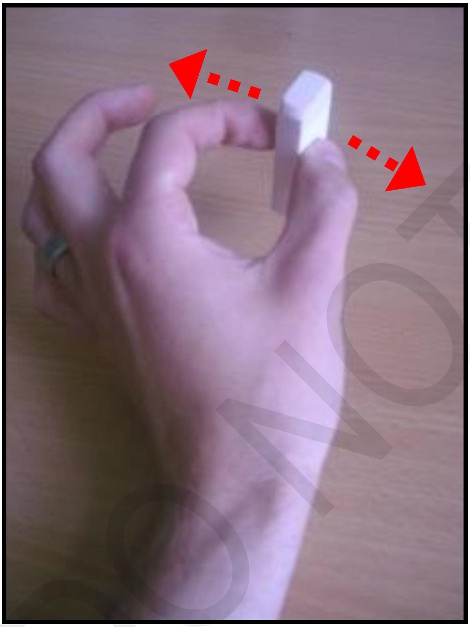

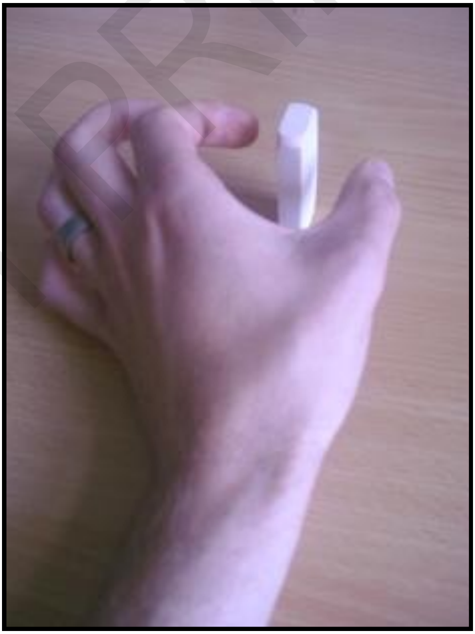

DO NOT PRINT

7.b.6

Pick up a coin

- Place your index finger and thumb either side of a coin with no other fingers touching the table
- Pick up the coin by stabilising with your thumb and pinching underneath with your index finger
- Try to replace on table as gently as possible

DO NOT PRINT

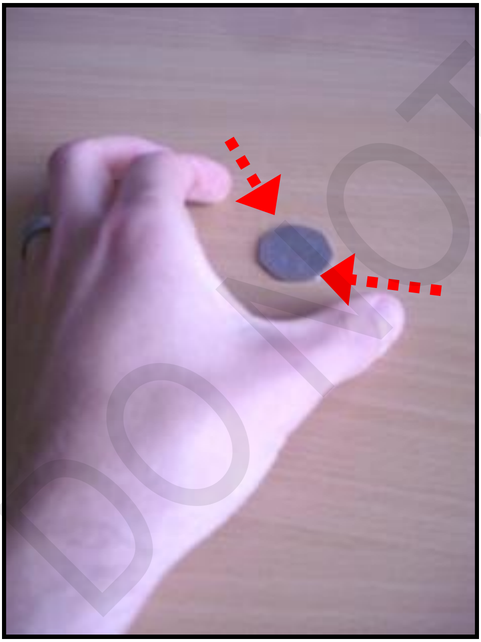

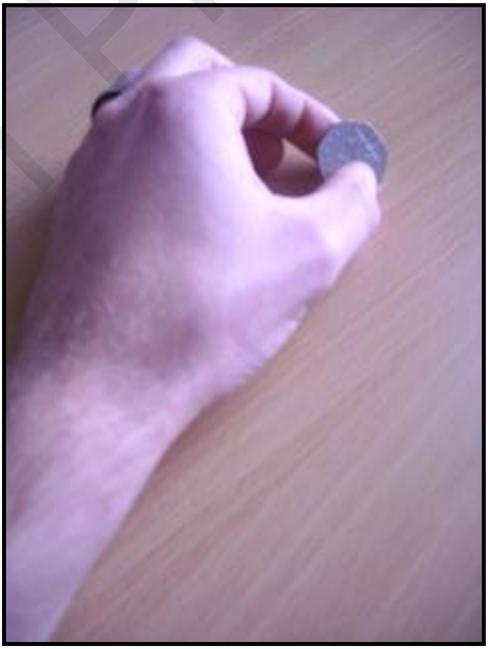

7.c.1

Pinch through cup handle

- Sit at a table
- Reach for a cup handle in front of you
- Form an ‘O-shape’ with your thumb and index finger through the cup handle without touching the cup
- Release and withdraw your hand again without touching the cup

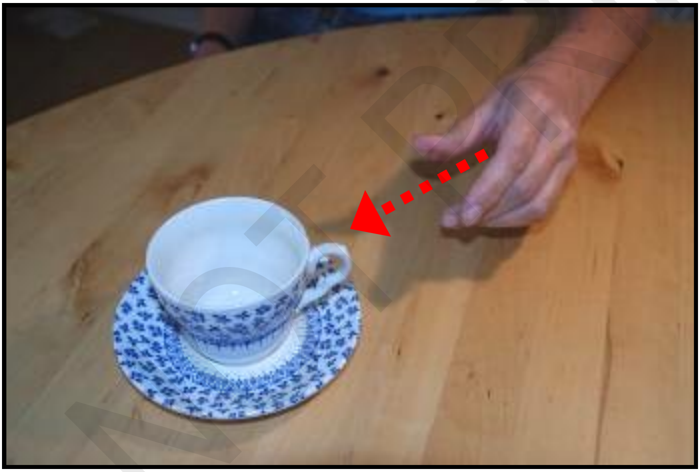

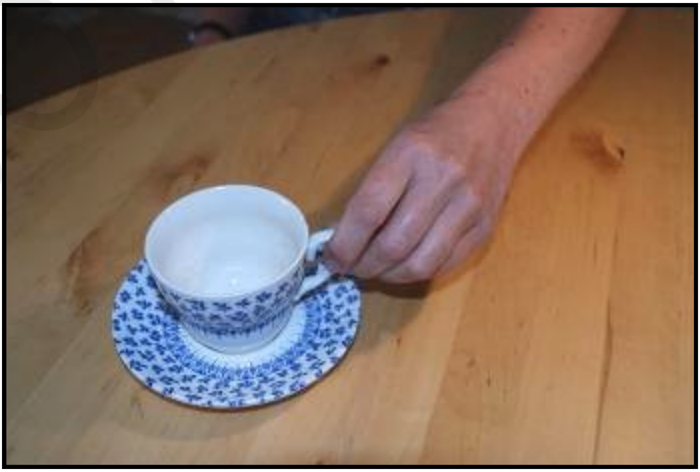

DO NOT PRINT

Take and replace small items on shelves

- Practice grasping and releasing small objects, for example herb jars, on shelves
- Your therapist can advise you on varying the heights and positions of the objects.
- Your therapist may also recommend to use only plastic / metal / non-breakable items

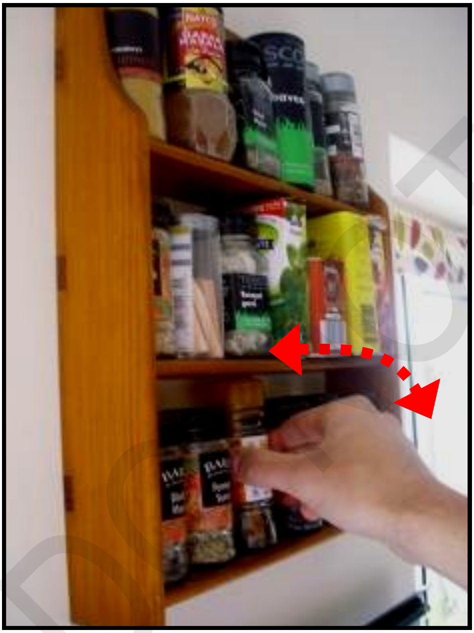

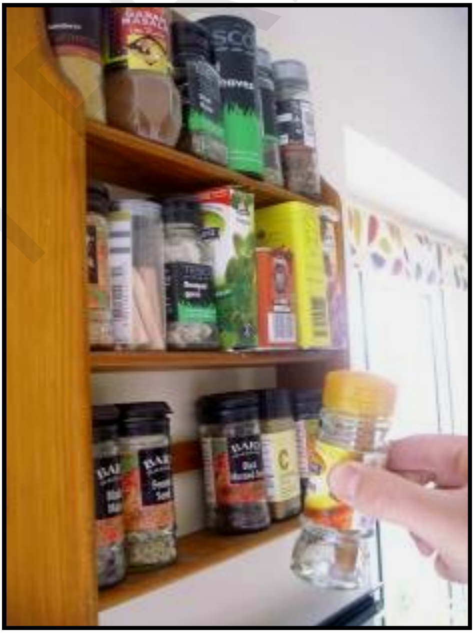

DO NOT PRINT

Maintain grasp using tweezers and a coin

- Sit with some plastic tweezers in your hand as shown.
- Place a coin in between the end of the tweezers and squeeze on the tweezers with your ring and little fingers to hold the coin.
- Practice turning your wrist over without dropping the coin, then practice bending your wrist with your wrist turned over.

DO NOT PRINT

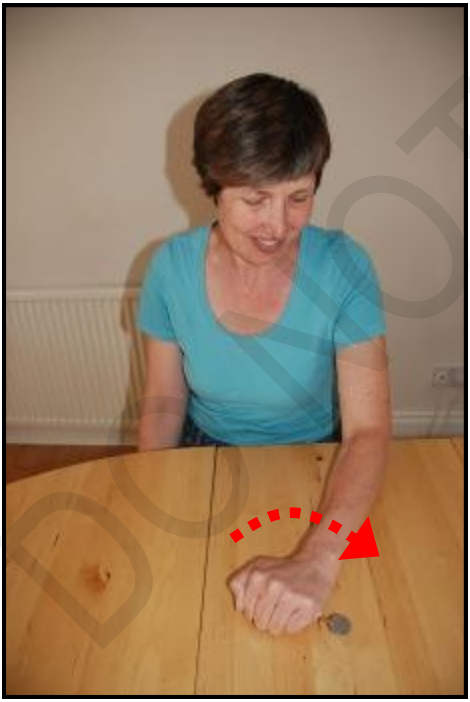

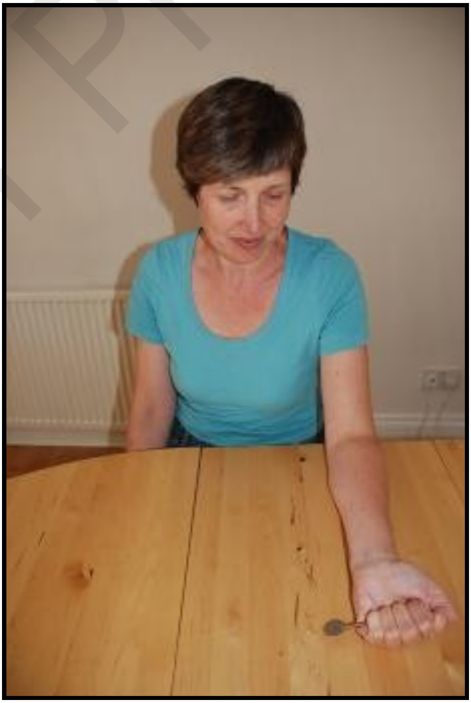

7.c.4

Buttoning and un-buttoning

- Wearing an item of clothing with buttons on practice un-buttoning with your weaker hand only
- Practice then buttoning up again - you will probably need to assist with your other hand

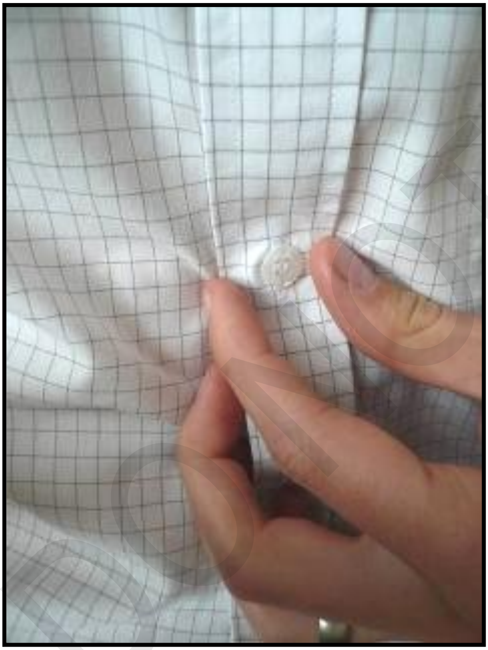

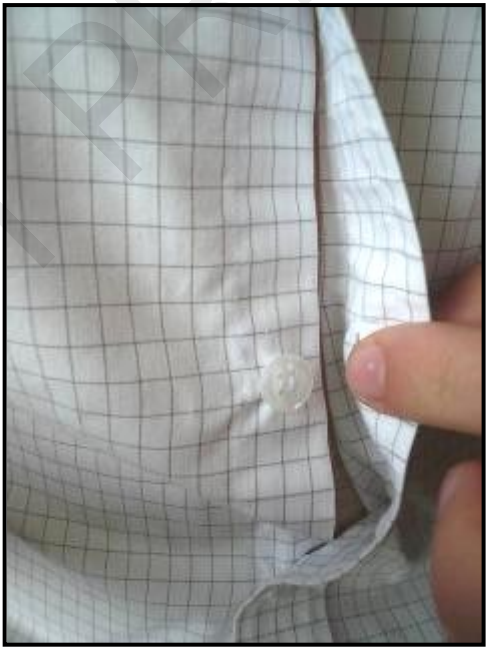

DO NOT PRINT

7.c.5

Turn pages

- Sitting or standing in front of a table
- Lay a newspaper / magazine / book out flat
- Practice turning the pages in both directions
- You may need to use your other hand to stabilise

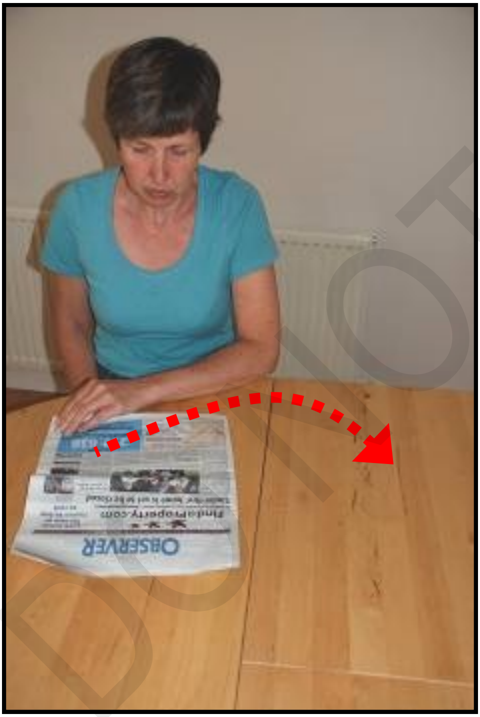

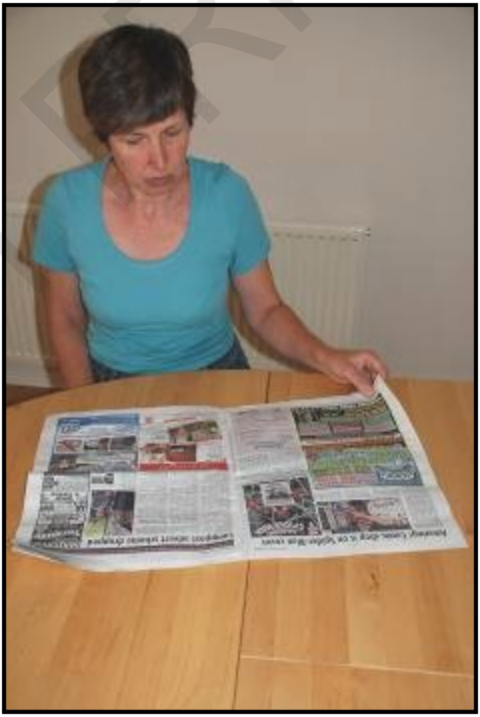

DO NOT PRINT

7.c.6

Fold washing on table

- Sitting or standing in front of a table
- Place an item of clothing flat on the table with your weaker hand only if possible
- Fold the item using your weaker hand only

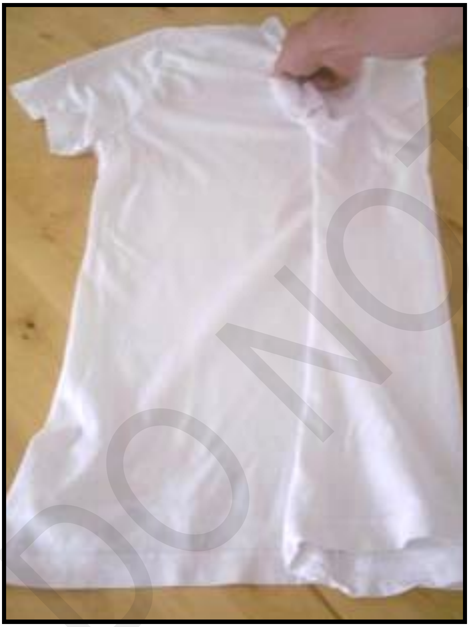

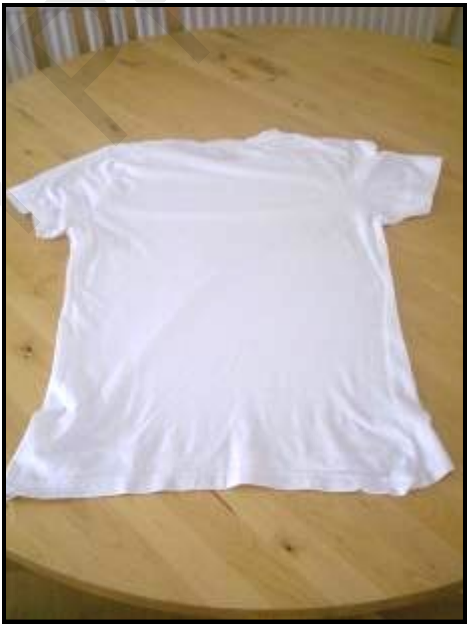

DO NOT PRINT

7.c.7

Remove and re-pin items on notice board

- Sitting or standing
- Remove and replace pin
- Change the position the pin is replaced each time
- Progress to pinning up paper

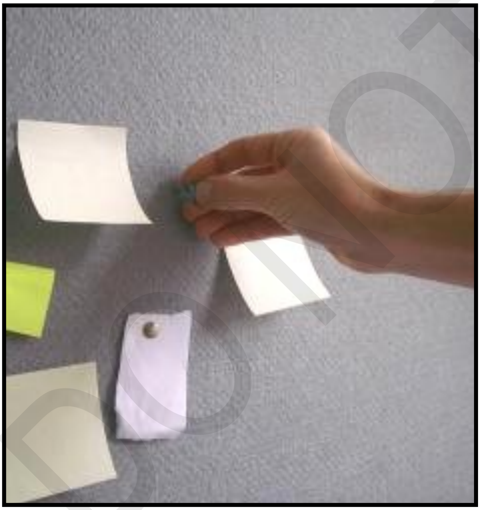

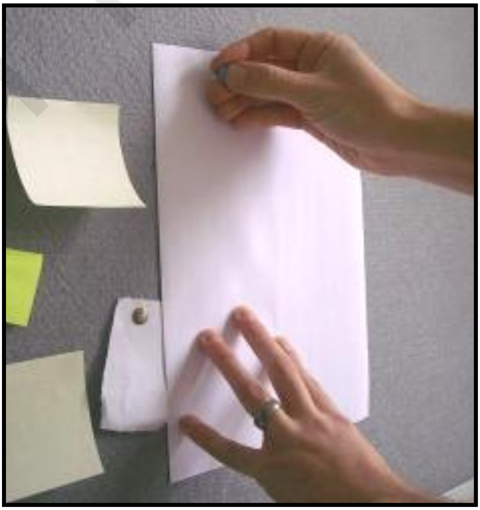

DO NOT PRINT

Turn over cards

- Practice taking one card at a time from stack and turning over
- Your therapist may also advise you to practice:
    - Shuffling cards
    - Fanning cards out
    - Dealing cards

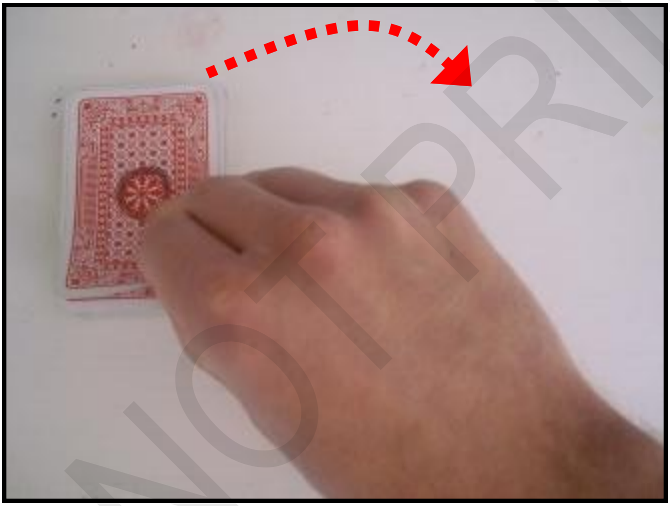

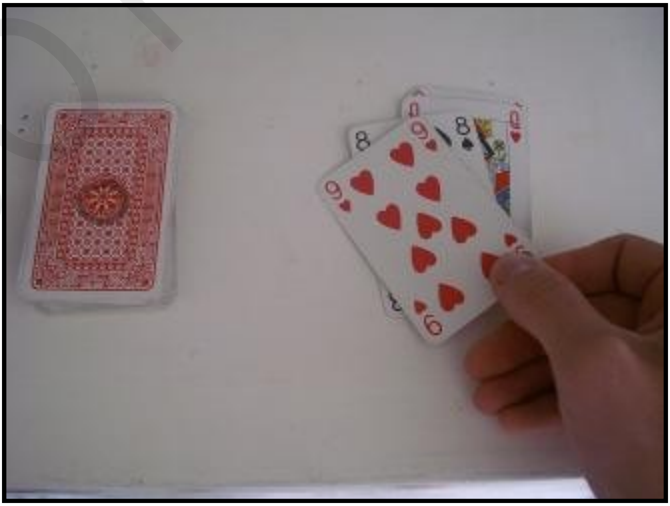

DO NOT PRINT

7.c.9

Play game involving dexterity

- Practice picking up pieces of games / puzzles between your thumb and index finger
- Your therapist may also advise on other games involving dexterity you could practice

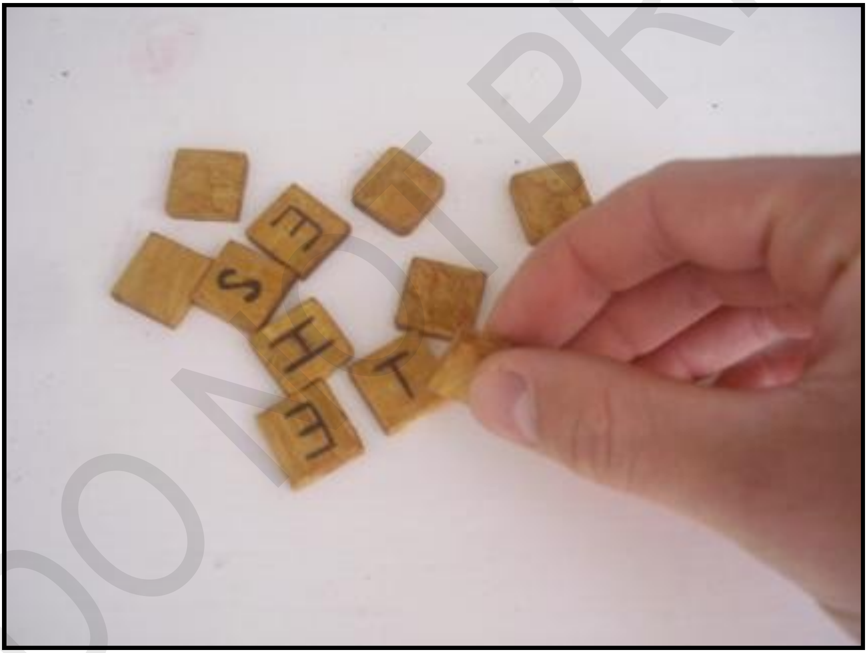

DO NOT PRINT

7.c.10

Place pegs in a peg board

- Sit with a peg board and some cylindrical pegs on a table in front of you.
- Practice picking up the pegs one at a time and placing them in the holes in the peg board.
- Ensure that your shoulder does not hitch and your trunk does not bend to the side when reaching.

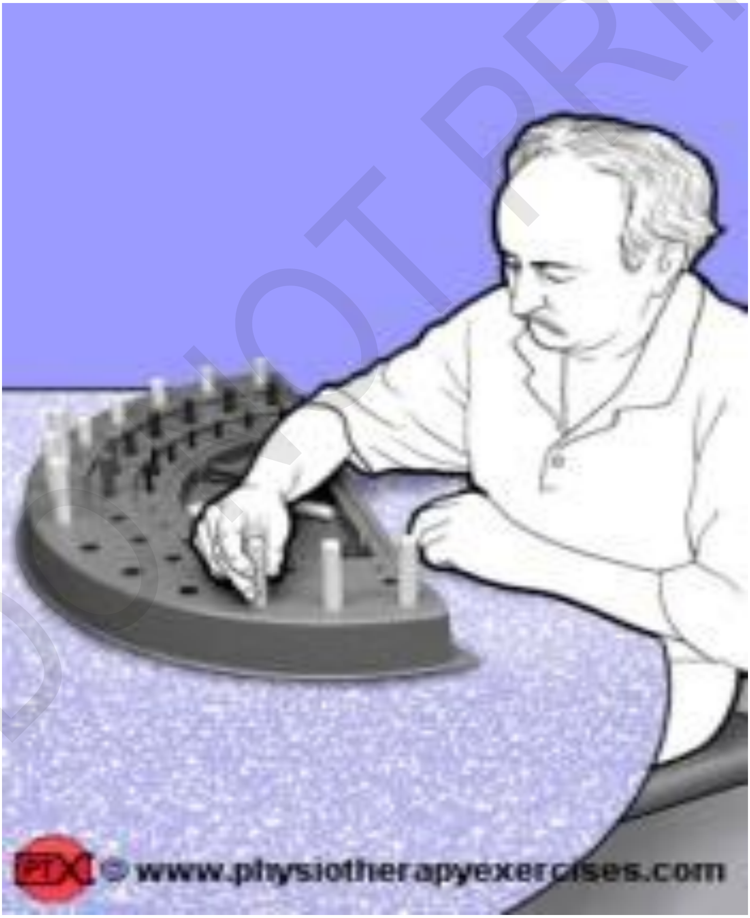

DO NOT PRINT

Take matches out of match box

- Practice taking individual matches out of a match box then placing back in the box

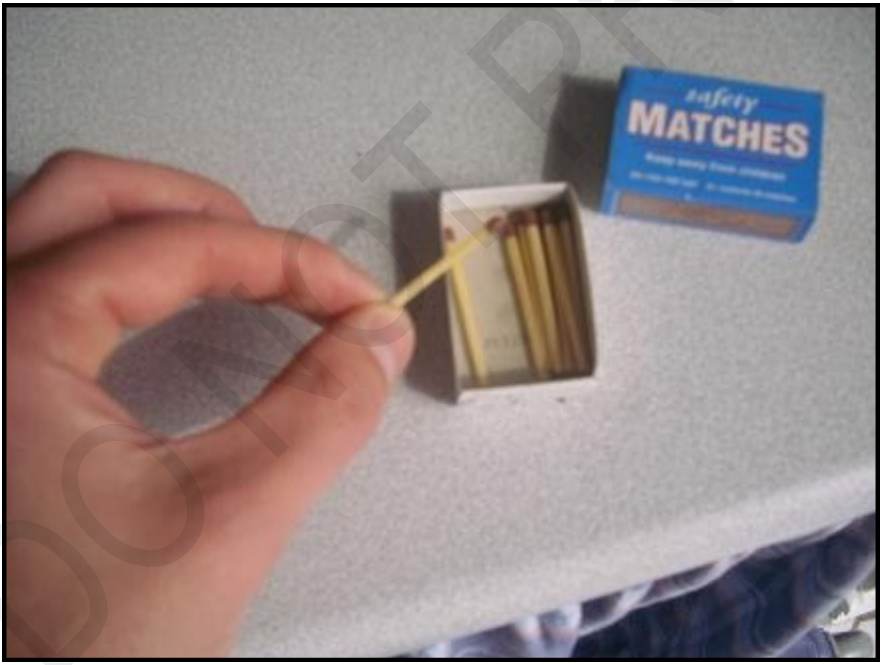

DO NOT PRINT

8.a.1

Cup hand to pick up a plate

- Sit with a plate on a table in front of you.
- Practice picking up the plate by placing your fingers underneath and your thumb on top of the plate.

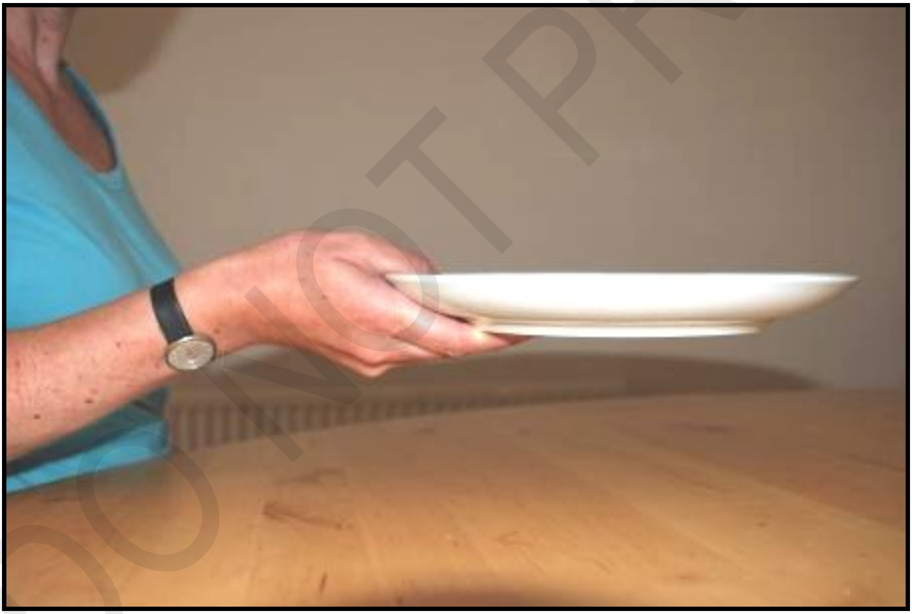

DO NOT PRINT

8.b.1

Pick up and move bowl

- Sit at a table with a bowl on a mat
- Pick up the bowl by reaching from above and spreading your fingers around the rim
- Lift the bowl off the mat and move to another mat on the table
- Place the bowl on the mat gently

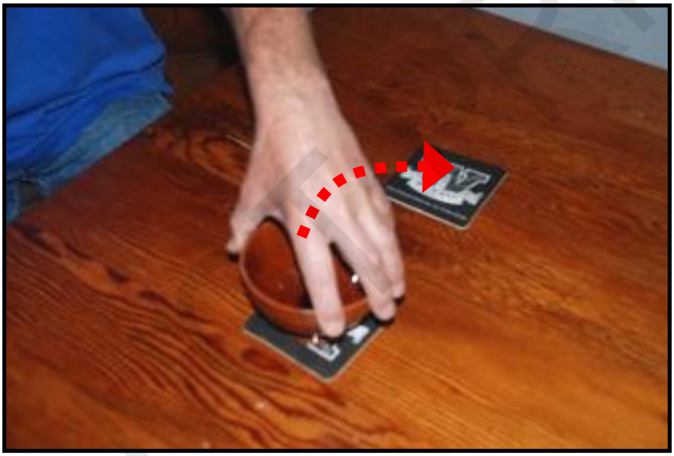

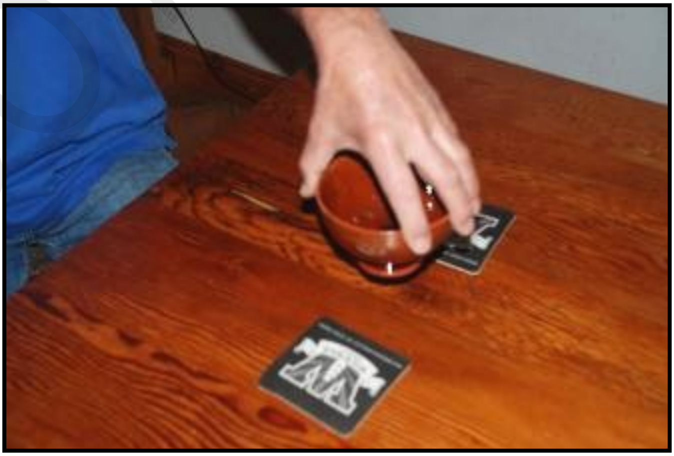

DO NOT PRINT

8.b.2

Unscrew jar lid

- Sit at a table with a jar in front of you
- The jar may need to be loosened by your therapist or carer
- Spread the fingers of your weaker hand around the jar lid and practice unscrewing and screwing back on
- Stabilise the jar with your other hand

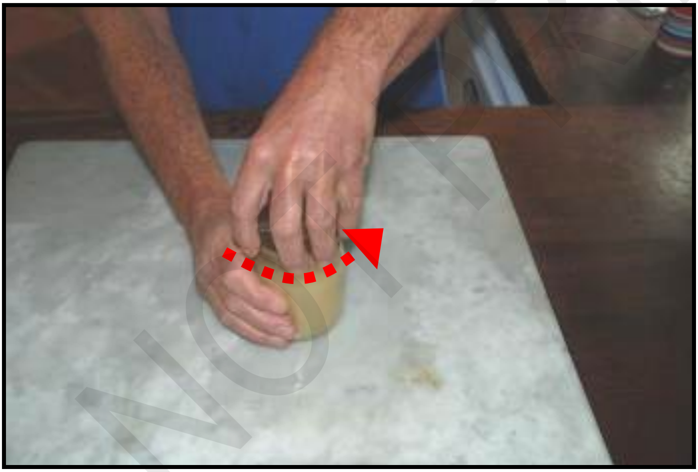

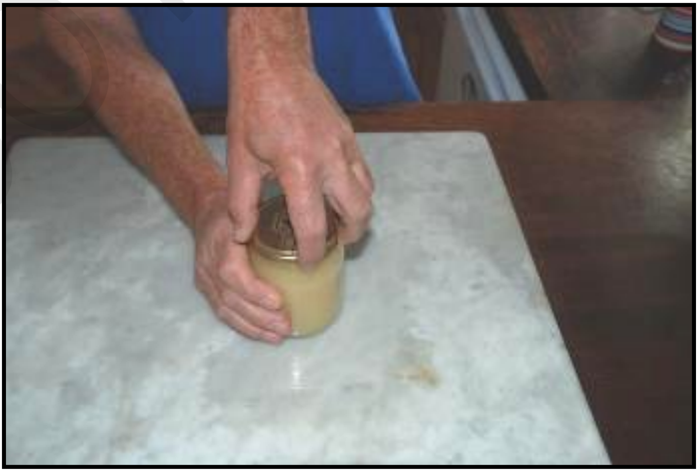

DO NOT PRINT

8.c.1

Stack cups

- Take one cup at a time from a stack and build a separate stack
- You will likely need to use your other hand to stabilise the cups

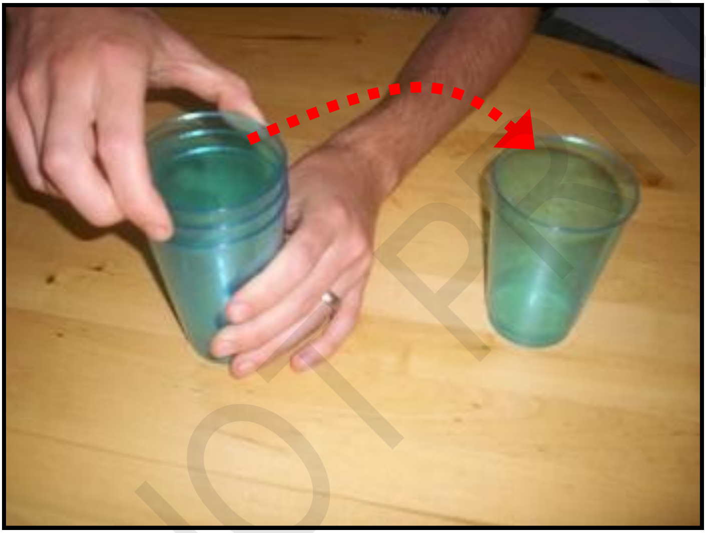

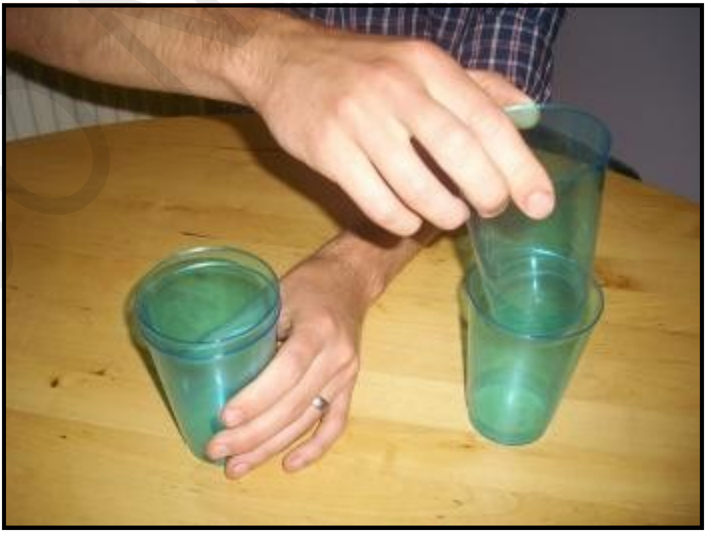

DO NOT PRINT

8.c.2

Stack coins

- Pick up coins one at a time and hold all the coins in your hand as you pick them up
- Try to stack the coins into a pile without dropping any.
- Once stacked, you can then try removing the coins from the stack one at a time

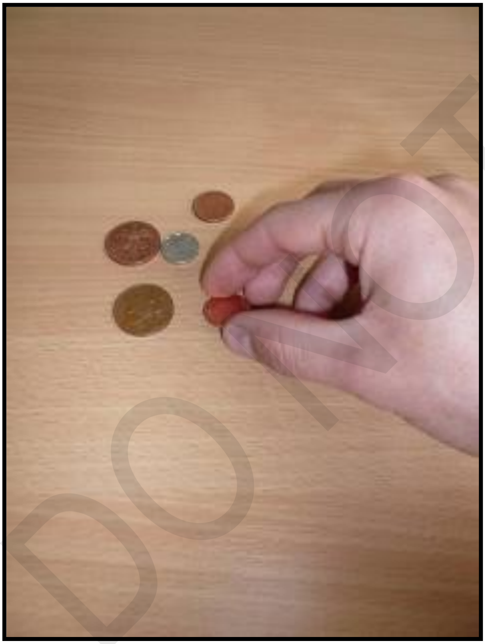

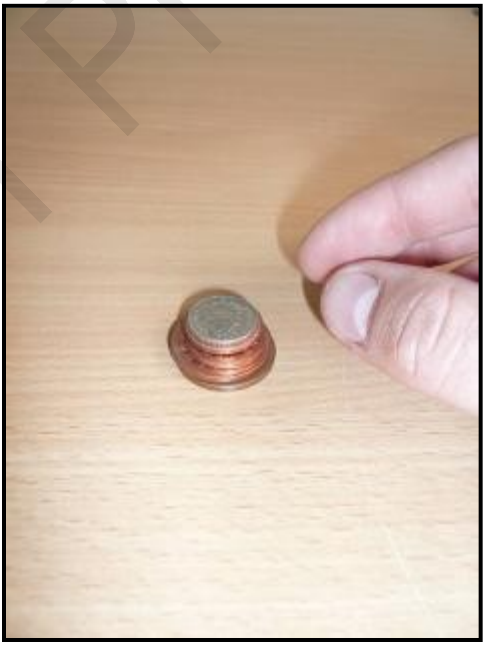

DO NOT PRINT

Use purse / wallet

- Practice using your purse / wallet.
- Specific tasks you can practice are:
  - Opening and closing the purse or wallet
  - Removing and replacing cards
  - Removing and replacing notes
  - Removing and replacing coins

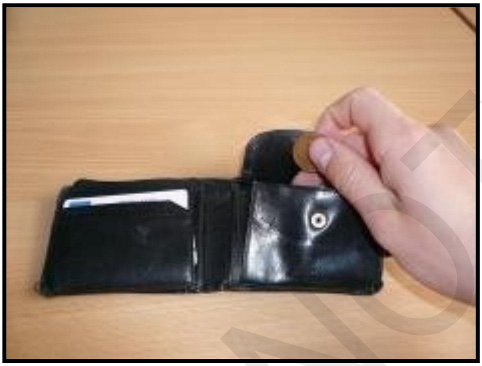

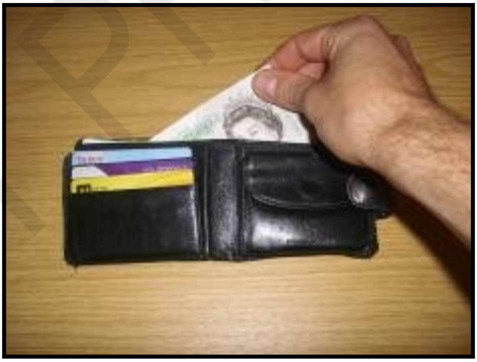

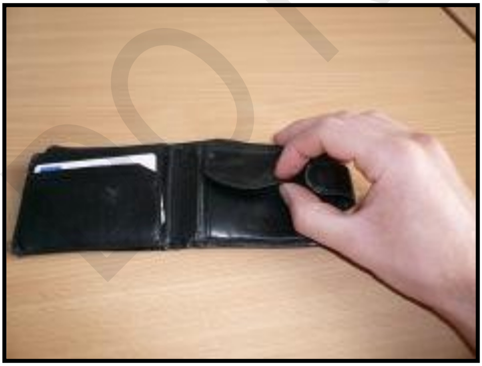

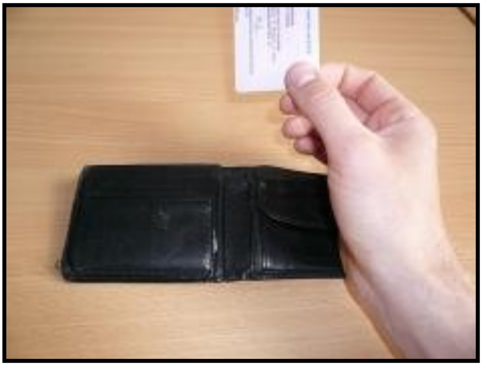

DO NOT PRINT

8.c.4

Choose key from a bunch

- Hold a bunch of keys in your weaker hand
- Practice choosing each key on the ring individually using your weaker hand only

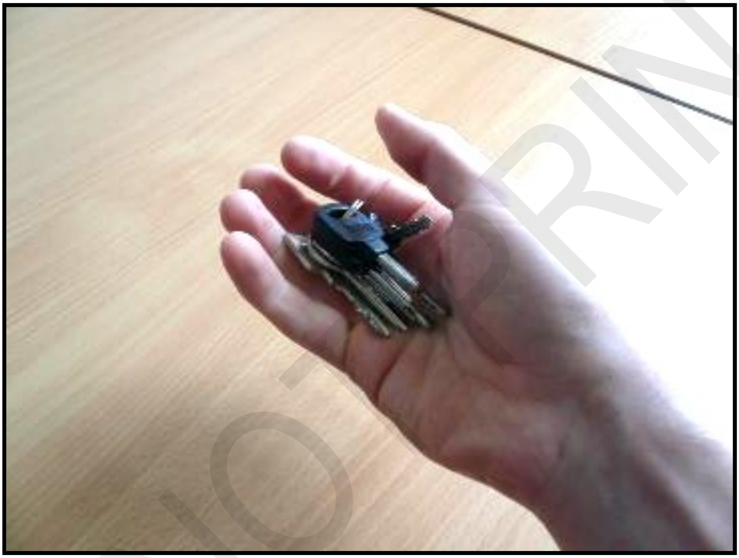

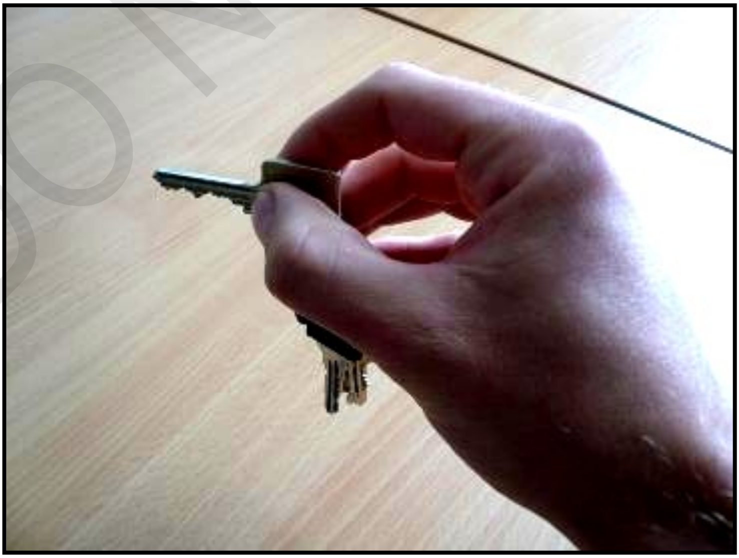

DO NOT PRINT

8.c.5

Make a chain of paperclips

- Pick up paper clips one at a time
- Hold all the paper clips in your hand as you pick them up
- Once you have picked them all up, try to make a chain out of them - you will need to use both hands for this part of the task

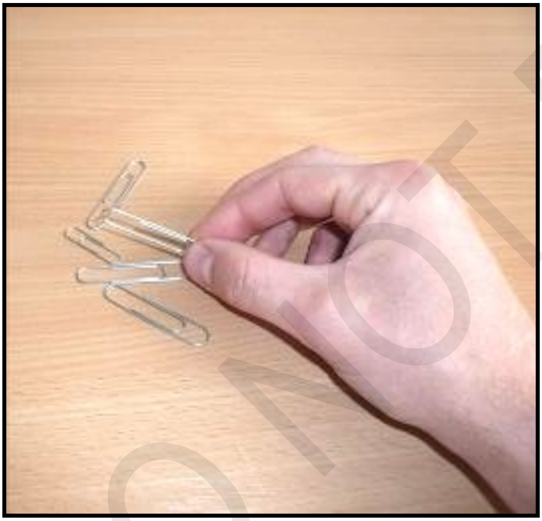

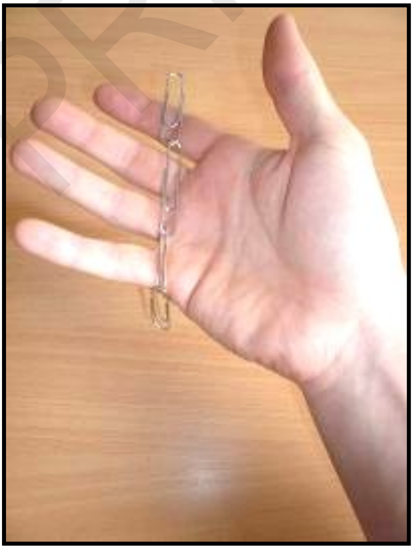

DO NOT PRINT

8.c.6

Tie and un-tie shoe laces

- Sitting
- Put your shoes on first
- Practice tying and untying your laces - you will need to use both hands

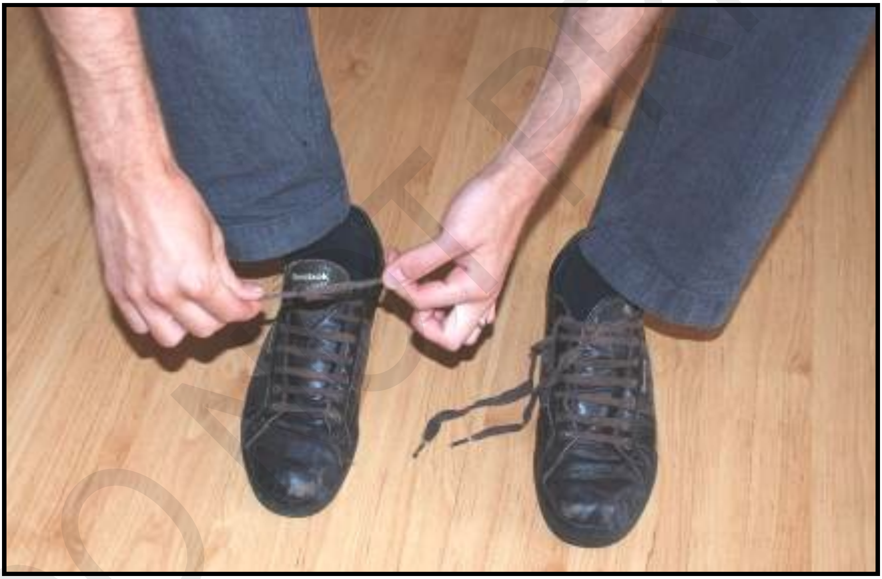

DO NOT PRINT

9.a.1

Use computer

- Practice typing and using the mouse

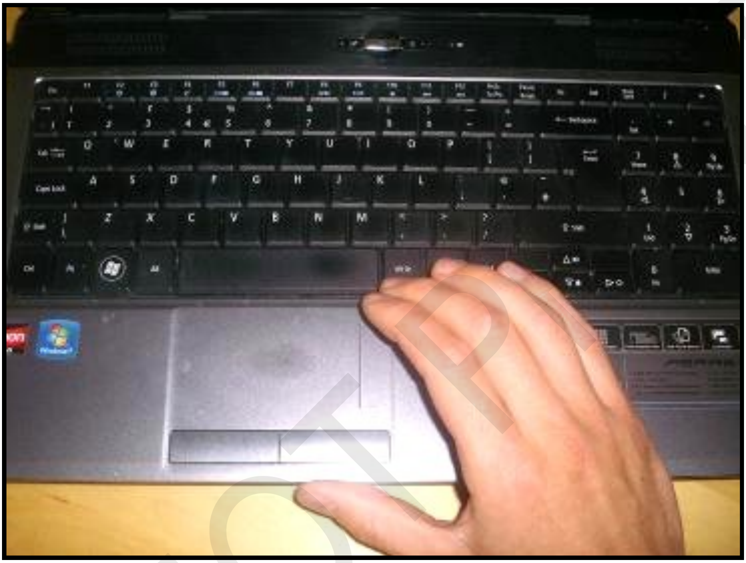

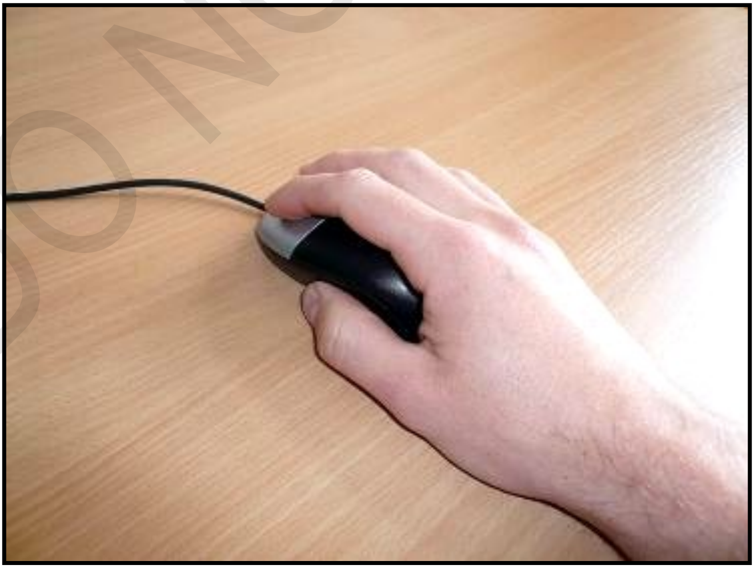

DO NOT PRINT

9.a.2

Knitting

- Practice knitting with your normal needles and wool

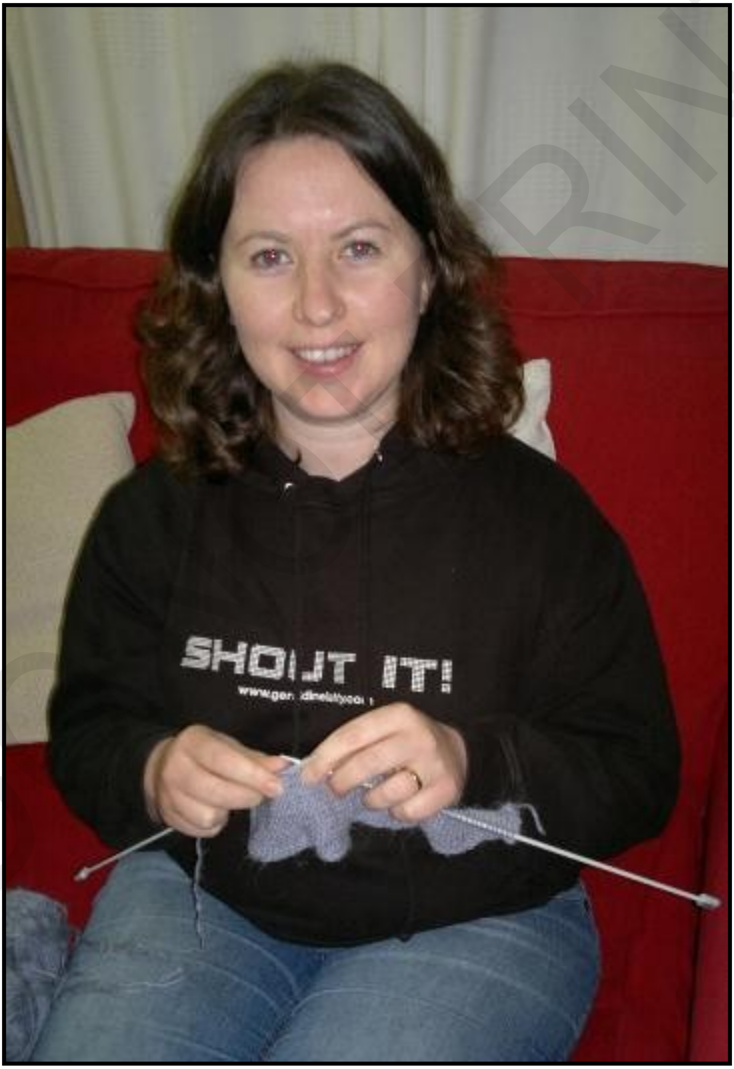

9.a.3

Wash up cutlery

- Hold cutlery in one hand and clean with the other
- Alternate which hand stabilises and which cleans

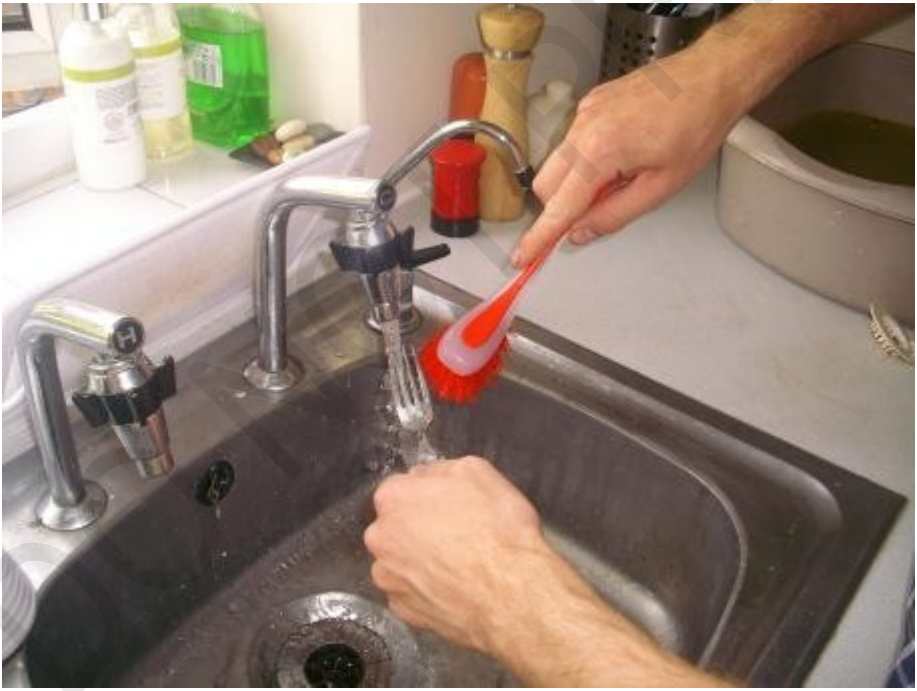

DO NOT PRINT

Wash up crockery

- Hold a plate in your weaker hand between your thumb and fingers with your hand cupped
- Clean the plate with your other hand
- Practice stacking in the draining board once clean

DO NOT PRINT

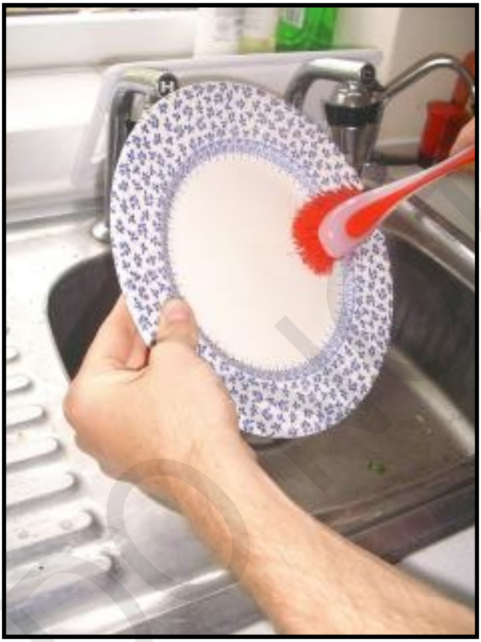

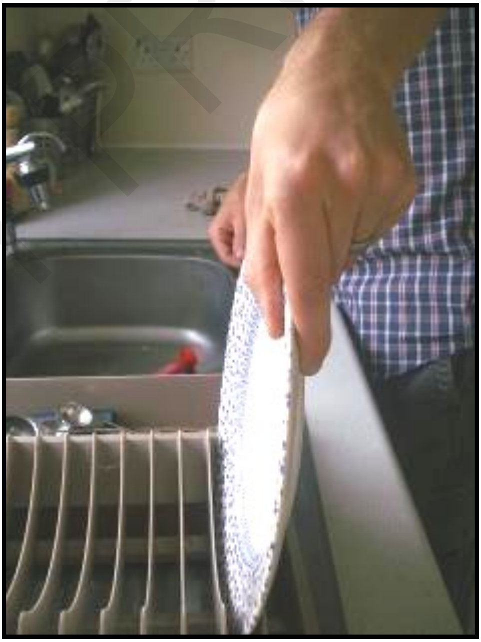

9.a.5

Brush teeth

- Can practice ‘teeth brushing action’ as an exercise without toothpaste, with water only
- When actually brushing your teeth also practice squeezing the tube with your weaker hand

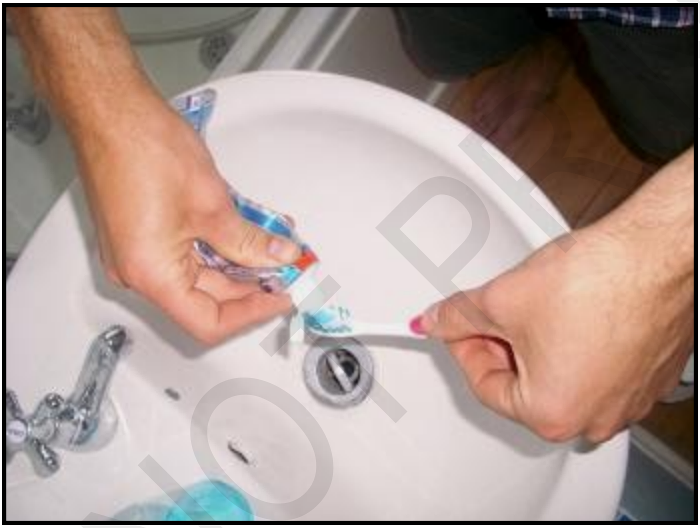

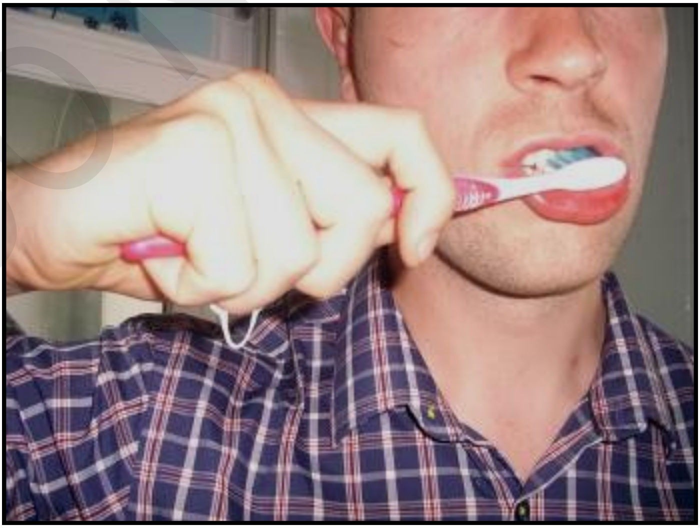

DO NOT PRINT

9.a.6

Shaving

- Practice shaving with the razor you normally use
- It may be safest to practice this initially with supervision - your therapist will give you advice on this

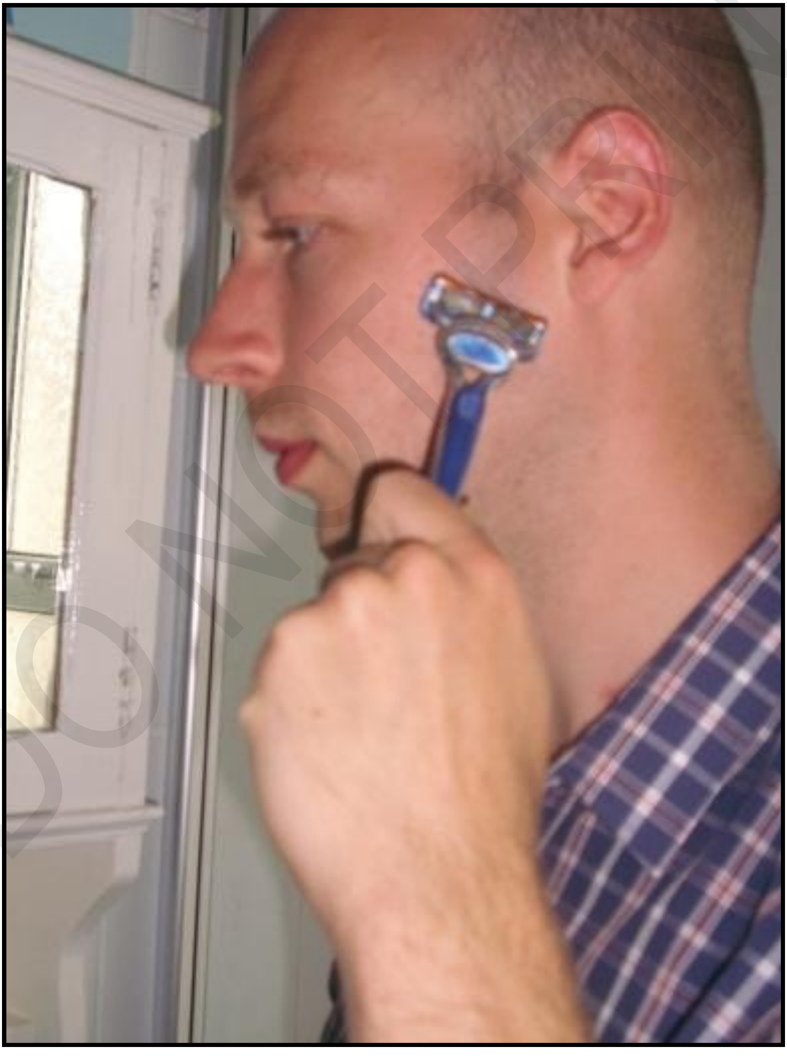

DO NOT PRINT

Brush hair

- Practice brushing your hair using the brush or comb you usually use

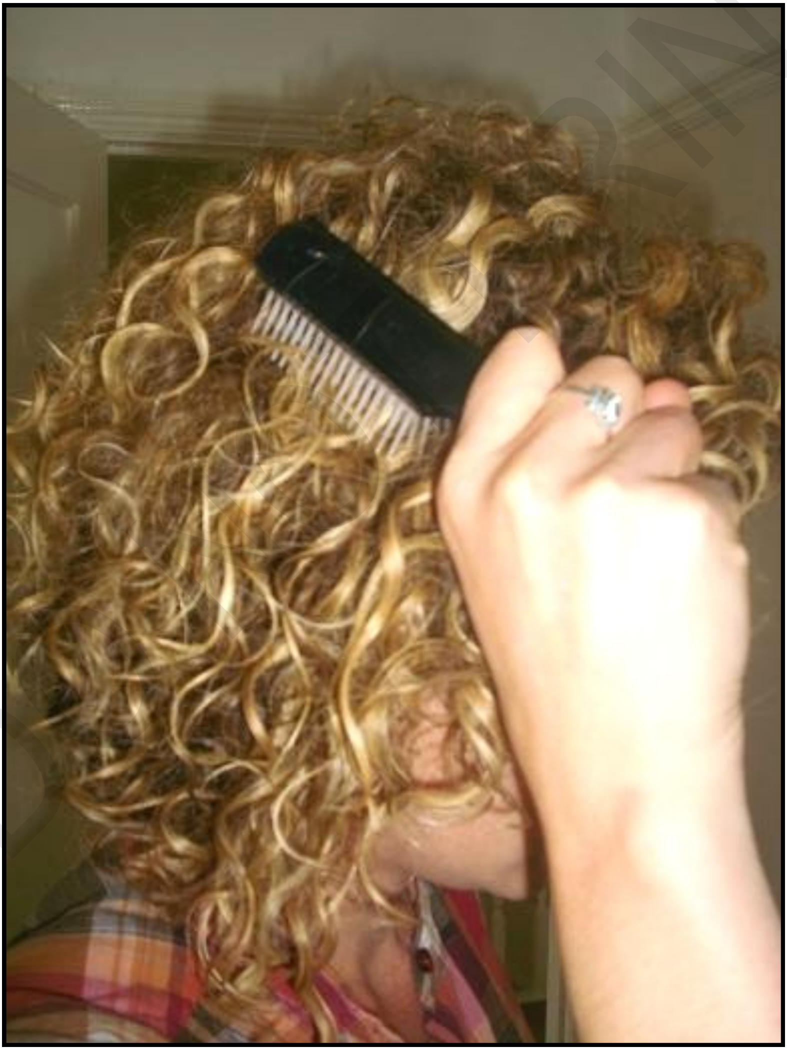

Writing

- Using your weaker hand only:
    - Pick up a pen
    - Remove the lid
    - Practice writing - you may need to steady the paper with your other hand

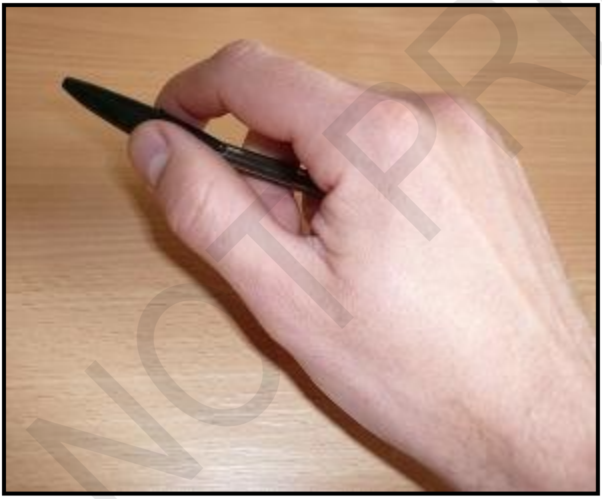

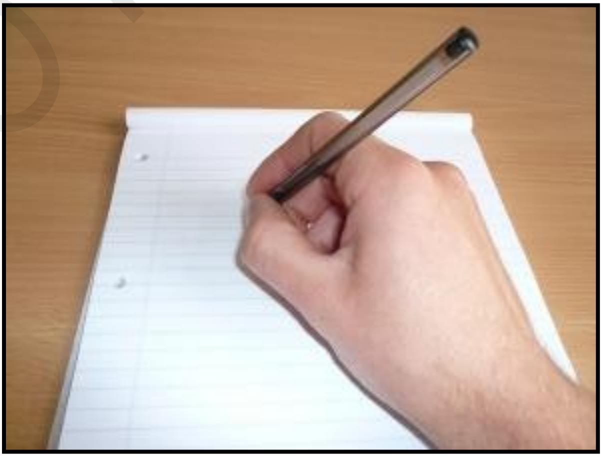

DO NOT PRINT

9.a.9

Peg out washing

- Using 2 hands, practice pegging items of clothing onto a washing line.

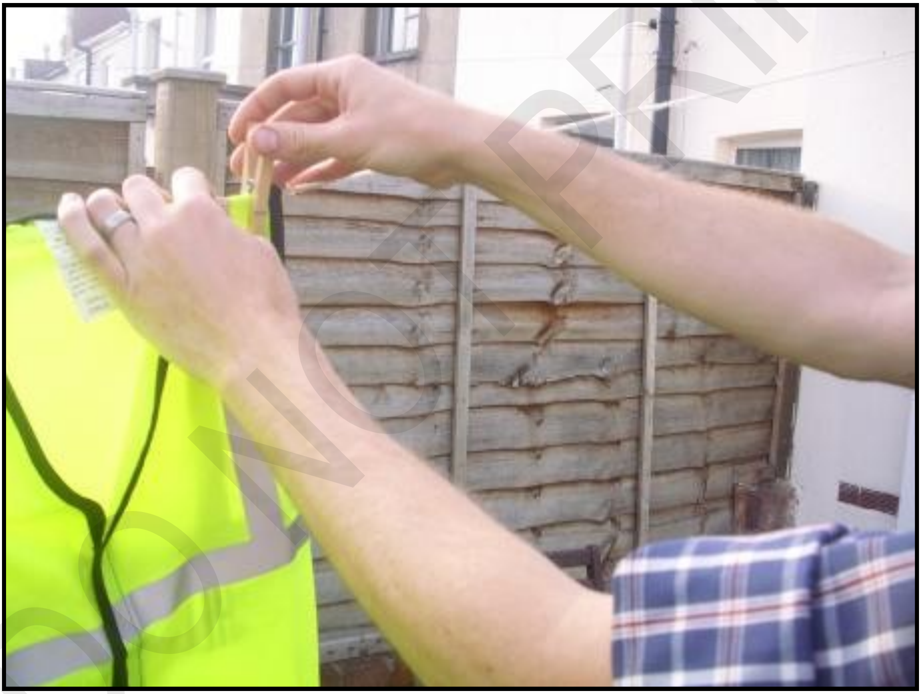

DO NOT PRINT
