# Supplementary material for: Home-based reach-to-grasp training for people after stroke: study protocol for a feasibility randomized controlled trial
Source: Trials. 2013 Apr 25;14:109. doi: 10.1186/1745-6215-14-109 (PMC3675391; doi:10.1186/1745-6215-14-109)
Supplement: Additional file 3 — Intervention group Recovery after Stroke booklet. [file 1745-6215-14-109-S3.pdf]

This trial is funded by
**The Stroke Association**

And supported by the
**Stroke Research Network**

***Thank you for reading this booklet***

Images on pages 3,4,5 and 6 of the booklet courtesy of
Jo Hutton and re-printed with kind permission.

DO NOT PRINT

University of the
West of England
BRISTOL

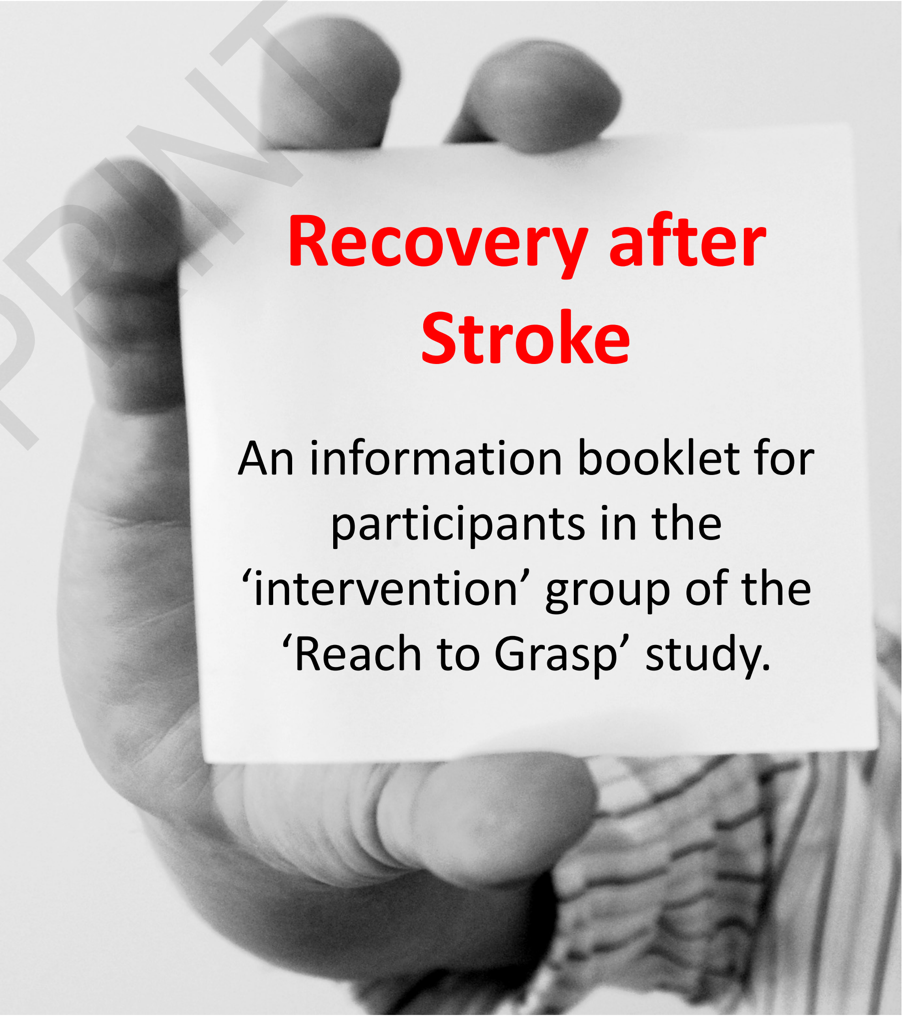


***North Bristol Trust, REC Version 1, 26/08/2011***

## About this booklet

You have agreed to take part in a pilot study for a randomised controlled trial of home based reach to grasp training for people after stroke.

This booklet explains how recovery occurs after stroke and how you can try to guide this recovery.

The booklet will cover:

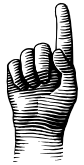

**What is a stroke?**

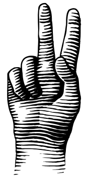

**Recovery after a stroke**

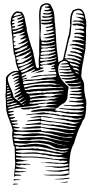

**How the brain can 're-wire'**

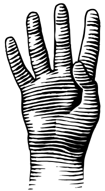

**Why practice is important**

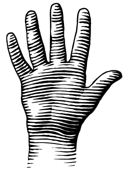

**What to practice**

Due to the focus of the study this booklet will concentrate specifically on recovery of the arm and hand.

DO NOT PRINT



## Occupational Therapy

The Occupational Therapy team provide specialist advice to adults to promote independence, goal orientated rehabilitation, provision of specialist equipment and adaptations, to support services users to live as independently as possible, in the community.

You may be referred by your GP **or may contact the service directly on 01454 868007.**

Further information is also available at their **website**:

**www.nbt.nhs.uk/our_services/a_-_z_of_services/s/south_gloucestershire_communit/rehabilitation.aspx**

## The Stroke Association

The **Stroke Association's** main focus is to support people who have had a stroke and their families. They do this by providing information and community services.

Information about services in your area, local contacts, news of local activities, details about stroke clubs and much more are available  is available on:

The **Stroke helpline:** **0303  3033  100**
Or on their **website:** **www.stroke.org.uk**

If you would like to talk to your local Stroke Association Information and Advice  coordinator please call **0117 953 1200** Monday-Friday 9am-5pm.

## Disabled Adult Resource Team (DART)

The DART service is a **community multi-disciplinary team of healthcare staff** who work with disabled adults with primarily physical impairments.

You can access this service by **speaking to your GP.**

DO NOT PRINT



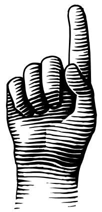

## What is a Stroke?

The **brain** is an important organ that does many things including **controlling movement**.

The brain is made up of lots of brain cells called **neurons**. These **need oxygen from blood** to work. Stroke is caused by something that stops the blood getting to parts of the brain and damages some of the **cells** in this part of the brain. This **stops them** from carrying out the jobs they would normally do and causes the symptoms of stroke.

Although these cells can't be repaired, **other parts** of the brain can learn to **take over** their **jobs.**

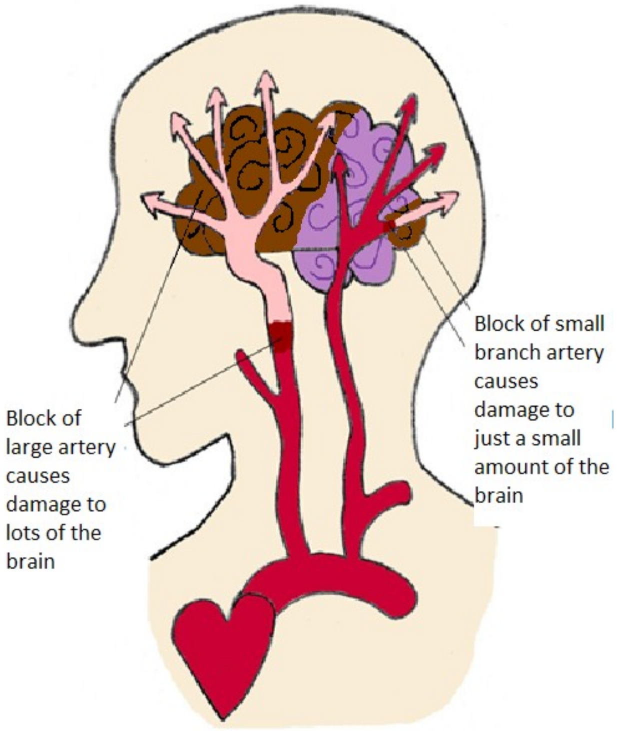

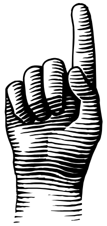

## What is a Stroke?

**There are 2 main types of stroke:**

### Ischaemic stroke

- Caused by a blood clot in an artery which blocks the flow of blood
- Cause of more than 8 out of 10 strokes

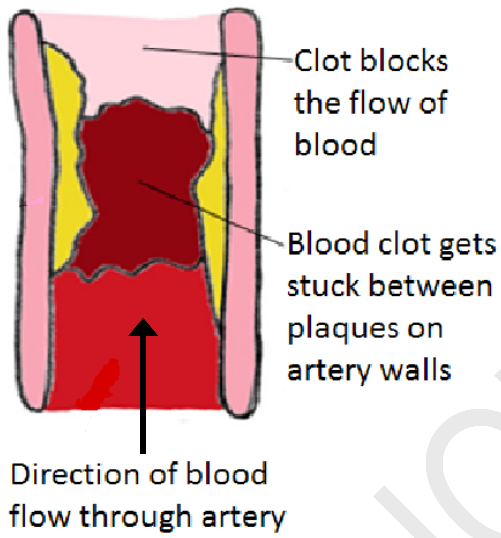


### Haemorrhagic stroke

- Caused by a weak or damaged blood vessel bursting and bleeding into the brain

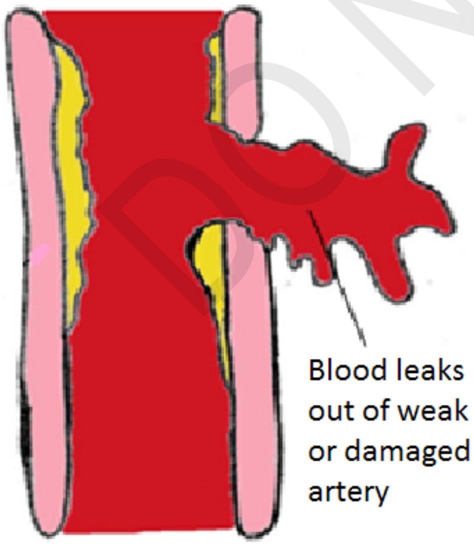




## Bristol Area Stroke Foundation (BASF)

**BASF** is a local organisation providing **social, emotional and practical support** to stroke affected individuals and their family. They provide a number of services including:

- **Individual counselling and support for stroke affected people and their carers**
- **Young stroke groups**
- **Stroke groups**

Stroke groups are run **weekly** in:

- **Fishponds**
- **Horfield**
- **Longwell Green**
- **Yate**

For further **advice and information**, please ring on:

**0117 964 7657**

Further information is also available on their **website** at:

[www.basf.uk.com](http://www.basf.uk.com)

The BASF also run a '**Garden for the Disabled'** group in a specially adapted glasshouse at Frenchay Hospital every Monday, Wednesday and Friday afternoon.

DO NOT PRINT

## After 6 Weeks

Once the 6 weeks of 'reach-to-grasp' therapy has finished, the **research physiotherapist** will no longer need to visit you. If you are currently receiving input from any **other community services** this **will not be affected.**

**If** at the end of the 6 weeks the research physiotherapist thinks you could benefit from further therapy input they **may** refer you.

Alternatively, you may wish to contact one of the **community services** available in your area which **do not require a referral from a health professional.**

Information about these and **how you can refer yourself** are detailed over the next few pages.

DO NOT PRINT



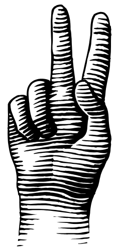

## Recovery after Stroke

After your stroke, you will need time to **heal** and **get back abilities** you have lost. This takes **time, practice, determination and patience. Your recovery** will be **unique** to you. Even if your symptoms are **similar** to someone else's, your ability to recover may be very **different**.

After an early burst of **recovery** in the first few weeks, progress tends to slow down. **This is normal.** Some people however continue to notice changes and improvements many months and even years after their stroke.

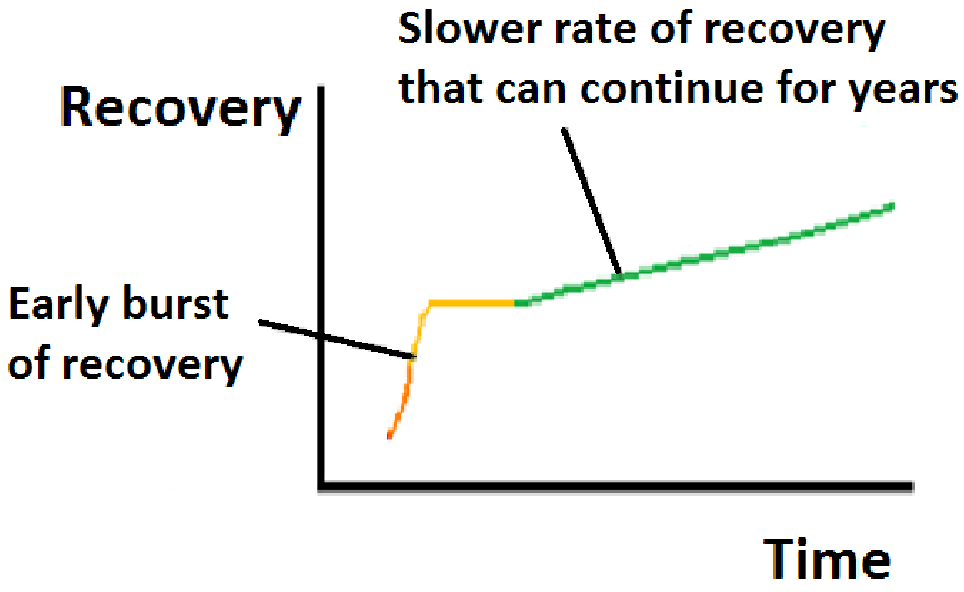

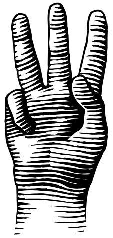

## How the brain can 're-wire'

Improvements after stroke can be attributed to a process called **neuroplasticity**. The word **neuroplasticity** comes from:

- **neuro** – meaning **'nerves'** and,
- **plasticity** – meaning **'mouldable'** or **'adaptable'**.

**Neuroplasticity** means therefore that **nerves**, which make up the brain, are **adaptable**. If **one area** of the brain gets **damaged**, **nearby areas** can **learn to take over** the job of that area. This means that if a **stroke disrupts** one area, the **brain can re-route signals** along different paths. This is like **redirecting traffic** if there is an accident on a major road.

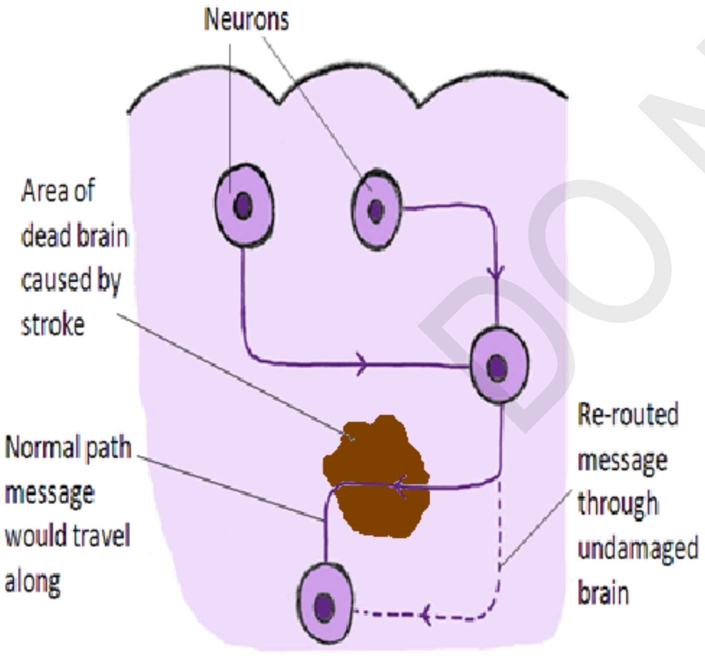




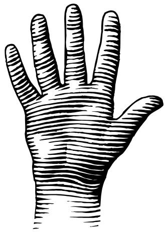

## What to Practice

Your therapist will **supervise** you at times to ensure you are happy with what they have **recommended you practice.** Over time they will change their recommendations to match your abilities.

It is **very important** that you **practise** what your therapist recommends **by yourself** as:

**The more you practise, the more recovery that is likely.**

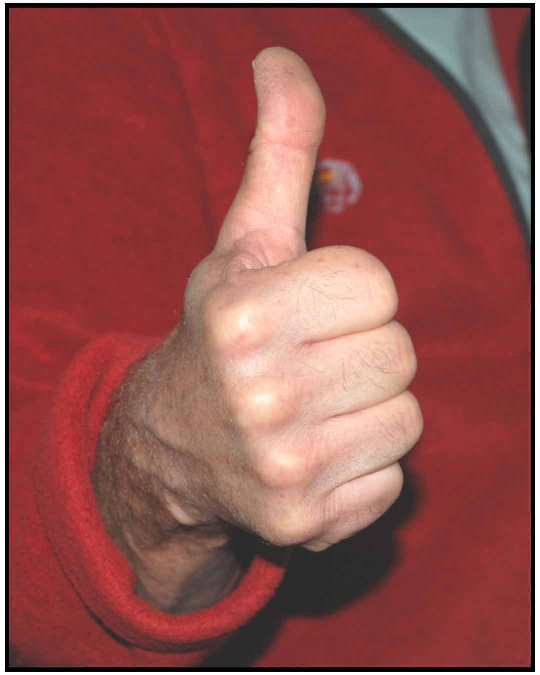

DO NOT PRINT

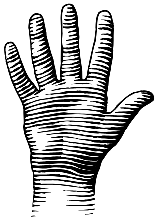

## What to Practice: Part Practice

It may be that you are not able to practice **whole** movements**.** If this is the case, your therapist can provide exercises in which you practice **part** of a movement. **For example**:

You may not presently be able to lift up a cup and place it on to a saucer:

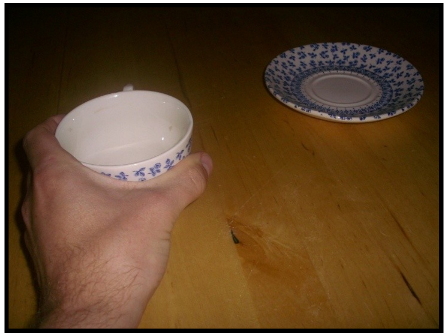

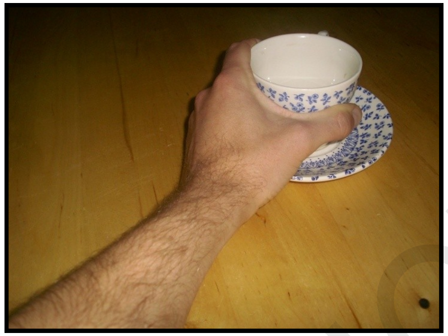

Instead your therapist **may** advise you to practice **sliding** a cup on the table.

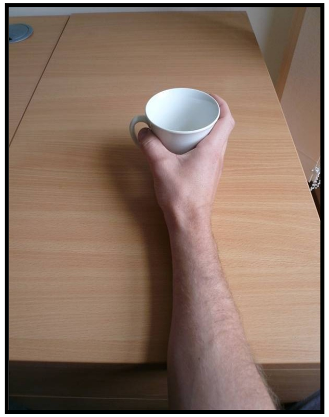

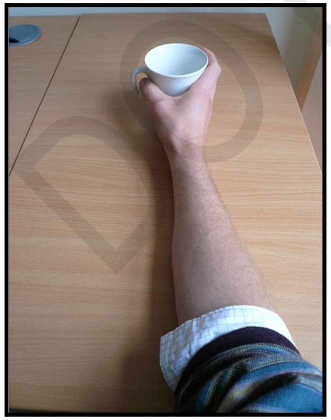

You could then **aim** to work up to being able to lift the cup.



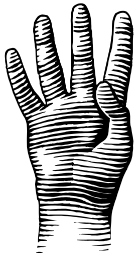

## Why is practice important?

The **nerves** in our brains are **constantly 're-wiring'** through our lives in response to our experiences. For example, **practice** of something, e.g. driving, sport or musical instruments, usually leads to **improvement**. These improvements are due in part to **neuroplasticity** in the brain. **Neuroplasticity** is especially important after a stroke.

The purpose of **rehabilitation** is to **guide** the **practice you do.** Your therapist will discuss with you what activities you are finding particularly difficult and will advise **exercises and tasks** for **you to practice.** Through **repetition** of these you can try to **'re-wire'** the brain to re-gain as much of your previous abilities as possible.

The rehabilitation you receive as part of this **research** will focus on your **arm and hand,** in particular, your ability to **reach to grasp** objects.

DO NOT PRINT

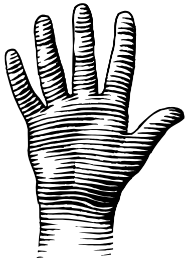

## What to Practice: Reach to Grasp

This research is about training **reach to grasp** movements.

**Reach to grasp** is essential for everyday tasks and is used **more frequently** than other arm movements.

We usually **reach to grasp** in order to **move or retrieve objects** we need to use. Some examples of this are:

**Getting dressed**

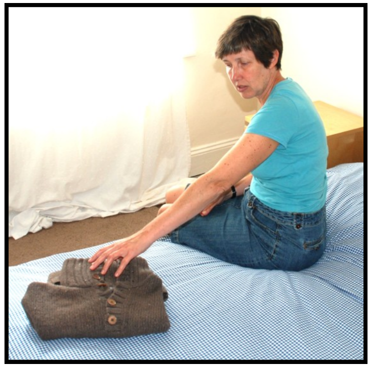

**Lifting and carrying shopping**

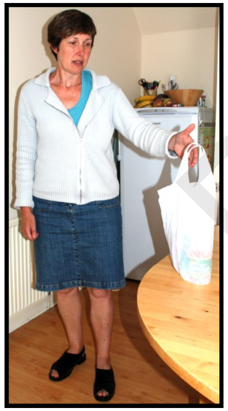

**Preparing and eating food**

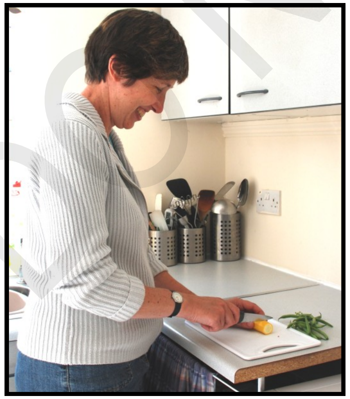



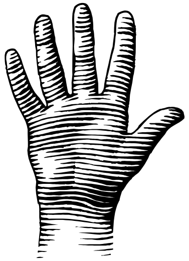

## What to Practice: Exercises

Your **therapist** for this study will **assess** your arm and hand function and will **recommend specific tasks and exercises** that are **most important** for you to **practice**. These **could** include:

**Turning a key**

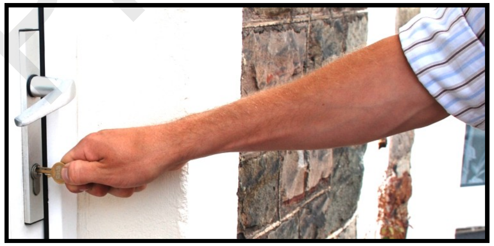

**Moving a pan between hobs**

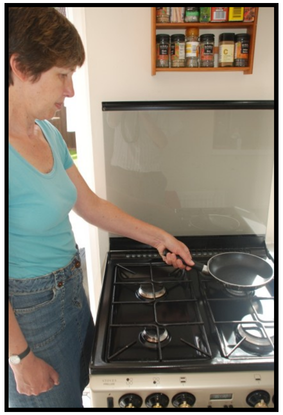

**Using your mobile phone**

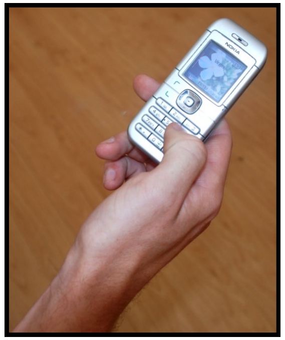

**Rolling, dropping, catching or throwing a ball**

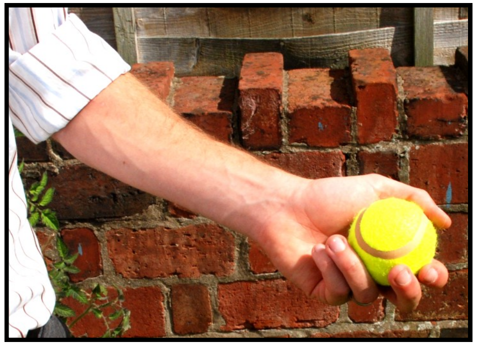

DO NOT PRINT
