# Supplementary material for: Home-based reach-to-grasp training for people after stroke: study protocol for a feasibility randomized controlled trial
Source: Trials. 2013 Apr 25;14:109. doi: 10.1186/1745-6215-14-109 (PMC3675391; doi:10.1186/1745-6215-14-109)
Supplement: Additional file 4 — Control group Recovery after Stroke booklet. [file 1745-6215-14-109-S4.pdf]

This trial is funded by
The Stroke Association

And supported by the
Stroke Research Network

Thank you for reading this booklet

DO NOT PRINT

Images on pages 3,4,5 and 6 of the booklet courtesy of
Jo Hutton and re-printed with kind permission.

University of the
West of England
BRISTOL

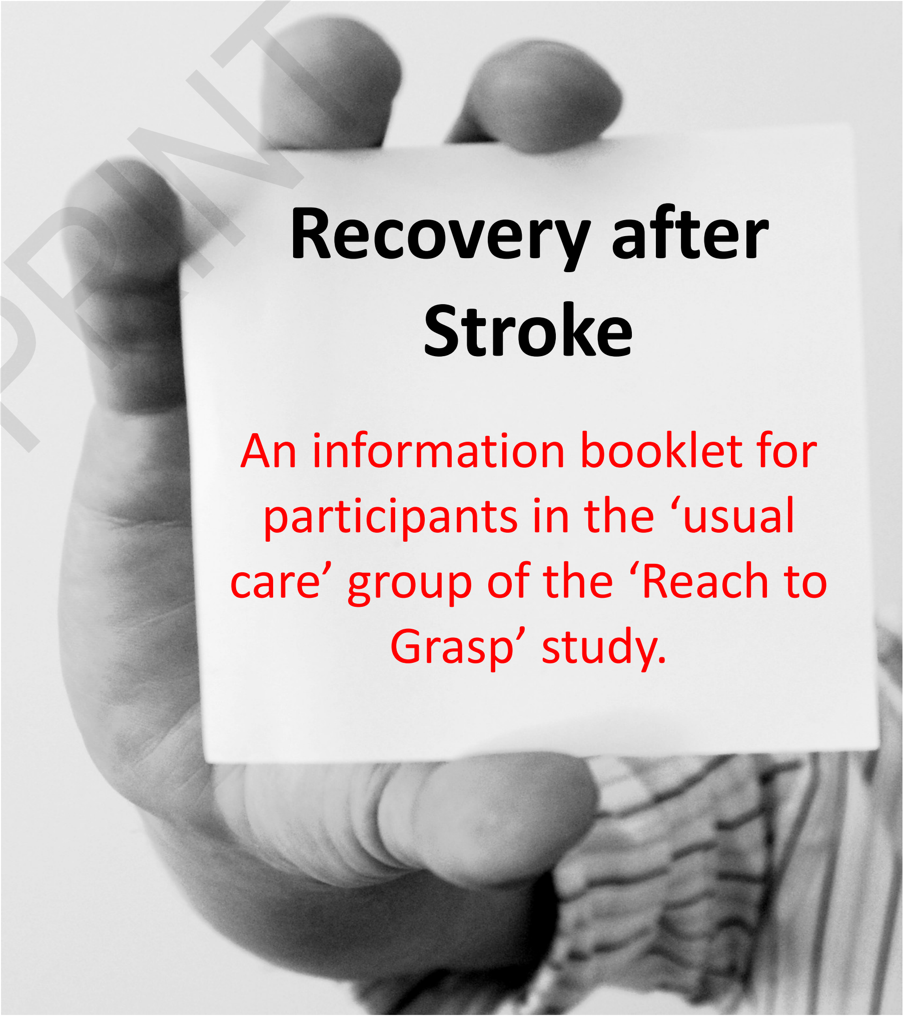

North Bristol Trust, REC Version 1, 26/08/2011

NHS

About this booklet

You have agreed to take part in a pilot study of home based reach to grasp training for people after stroke. You have been allocated to the 'intervention' group.

This booklet explains how recovery occurs after stroke and how you can try to guide this recovery.

The booklet will cover:

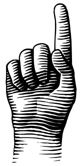

What is a stroke?

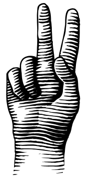

Recovery after a stroke

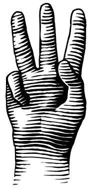

How the brain can 're-wire'

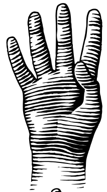

Why practice is important

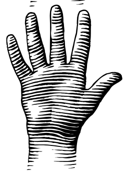

Study participation

DO NOT PRINT

Bristol Area Stroke Foundation (BASF)

BASF is a local organisation providing **social, emotional and practical support** to stroke affected individuals and their family. They provide a number of services including:

- **Individual counselling and support for stroke affected people and their carers**
- **Young stroke groups**
- **Stroke groups**

Stroke groups are run **weekly** in:

- **Fishponds**
- **Horfield**
- **Longwell Green**
- **Yate**

For further **advice and information**, please ring on:

0117 964 7657

Further information is also available on their **website** at:

www.basf.uk.com

The BASF also run a '**Garden for the Disabled'** group in a specially adapted glasshouse at Frenchay Hospital every Monday, Wednesday and Friday afternoon.

The Stroke Association

The **Stroke Association's** main focus is to support people who have had a stroke and their families. They do this by providing information and community services.

Information about services in your area, local contacts, news of local activities, details about stroke clubs and much more are available is available on:

The **Stroke helpline:** **0303 3033 100**
Or on their **website:** **www.stroke.org.uk**

If you would like to talk to your local Stroke Association Information and Advice coordinator please call **0117 953 1200** Monday-Friday 9am-5pm.

Disabled Adult Resource Team (DART)

The DART service is a **community multi-disciplinary team of healthcare staff** who work with disabled adults with primarily physical impairments.

You can access this service by **speaking to your GP.**

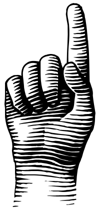

What is a Stroke?

The **brain** is an important organ that does many things including **controlling movement**.

The brain is made up of lots of brain cells called **neurons**. These **need oxygen from blood** to work. Stroke is caused by something that stops the blood getting to parts of the brain and damages some of the **cells** in this part of the brain. This **stops them** from carrying out the jobs they would normally do and causes the symptoms of stroke.

Although these cells can't be repaired, **other parts** of the brain can learn to **take over** their **jobs.**

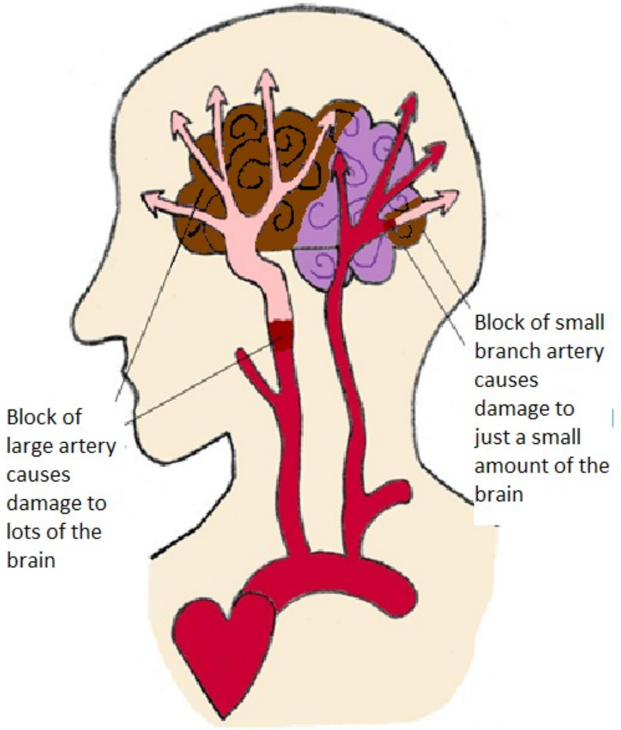

DO NOT PRINT

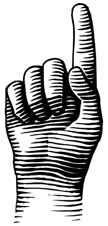

What is a Stroke?

There are 2 main types of stroke:

Ischaemic stroke

- Caused by a blood clot in an artery which blocks the flow of blood
- Cause of more than 8 out of 10 strokes

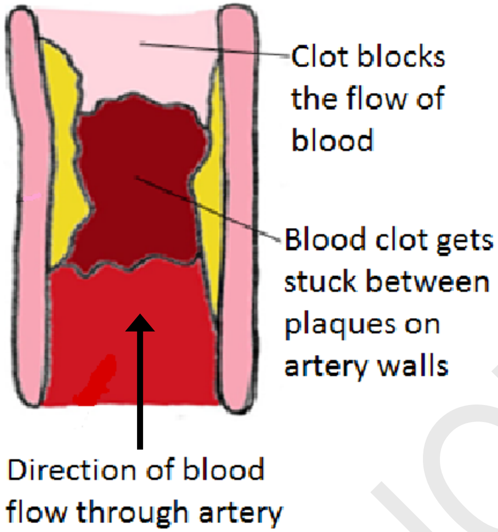

Haemorrhagic stroke

- Caused by a weak or damaged blood vessel bursting and bleeding into the brain

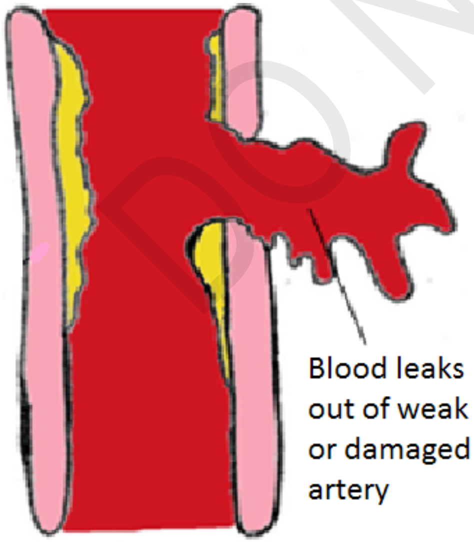

Available Services

If the **physiotherapist** thinks you could **benefit from further therapy input** they **may** refer you.

Alternatively, you can refer yourself at any time to the **community services** detailed over the next few pages:

DO NOT PRINT

Occupational Therapy

The Occupational Therapy team provide specialist advice to adults to promote independence, goal orientated rehabilitation, provision of specialist equipment and adaptations, to support services users to live as independently as possible, in the community.

You may be referred by your GP **or may contact the service directly on 01454 868007.**

Further information is also available at their **website**:

www.nbt.nhs.uk/our_services/a_-_z_of_services/s/south_gloucestershire_communit/rehabilitation.aspx

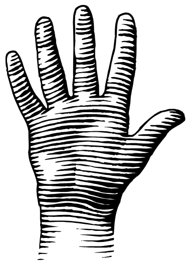

Study Participation

A **researcher** will visit you when you first consent to the study to complete a number of **assessments.** They will then visit you again six weeks, 3 months and 6 months later to repeat these assessments.

Once these assessments have been completed, if you are not already receiving therapy services, you will be offered **a 1 hour visit from a physiotherapist.**

At this visit the physiotherapist **may**:

- **Discuss** with you what you are currently finding difficult as a result of weakness in your arm or hand, and what you would like to be able to do
- **Assess** the movement and function of your arm which has been affected by stroke
- Provide you with **advice, exercises or activities** to continue to practice by yourself

Within the session, the physiotherapist will **supervise** you doing any exercises that they recommend to ensure you are happy with them.

DO NOT PRINT

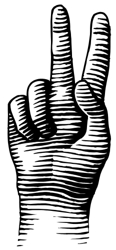

Recovery after Stroke

After your stroke, you will need time to **heal** and **get back abilities** you have lost. This takes **time, practice, determination and patience. Your recovery** will be **unique** to you. Even if your symptoms are **similar** to someone else's, your ability to recover may be very **different**.

After an early burst of **recovery** in the first few weeks, progress tends to slow down. **This is normal.** Some people however continue to notice changes and improvements many months and even years after their stroke.

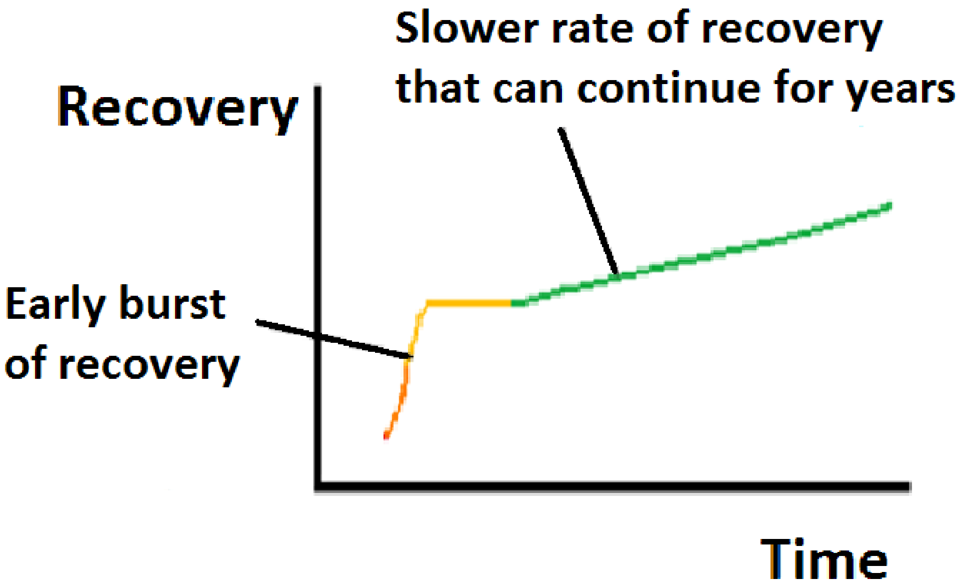

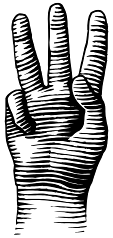

How the brain can 're-wire'

Improvements after stroke can be attributed to a process called **neuroplasticity**. The word **neuroplasticity** comes from:

- **neuro** – meaning **'nerves'** and,
- **plasticity** – meaning **'mouldable'** or **'adaptable'**.

Neuroplasticity means therefore that **nerves**, which make up the brain, are **adaptable**. If **one area** of the brain gets **damaged**, **nearby areas** can **learn to take over** the job of that area. This means that if a **stroke disrupts** one area, the **brain can re-route signals** along different paths. This is like **redirecting traffic** if there is an accident on a major road.

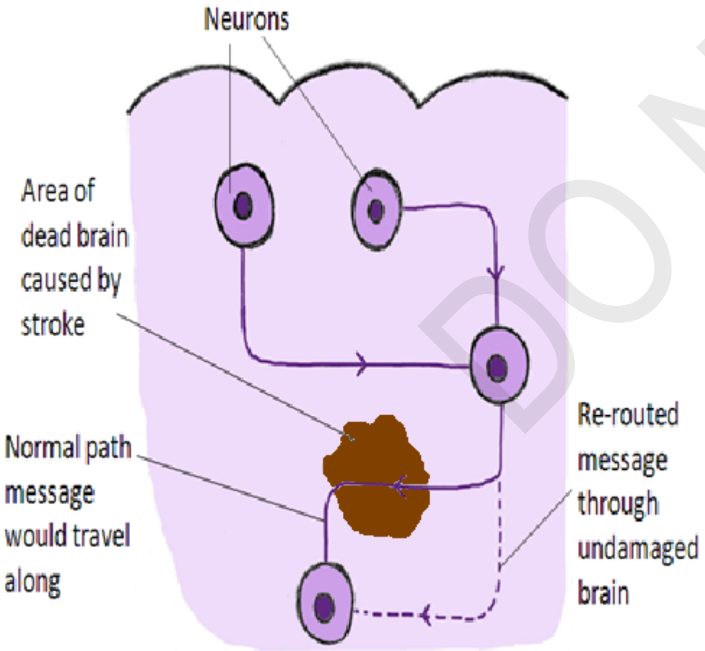

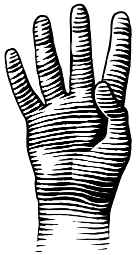

Why is practice important?

The **nerves** in our brains are **constantly 're-wiring'** through our lives in response to our experiences. For example, **practice** of something, e.g. driving, sport or musical instruments, usually leads to **improvement**. These improvements are due in part to **neuroplasticity** in the brain. **Neuroplasticity** is especially important after a stroke.

You have been allocated to the **control** group of this study. As such, you will continue to receive your **usual care**. This **may or may not** include community therapy services.

You should **continue to practice** any exercises or activities that have been recommended to you. Through **repetition** of these you can try to **'re-wire'** the brain to re-gain as much of your previous abilities as possible.

The more you practice, the more recovery that is likely.

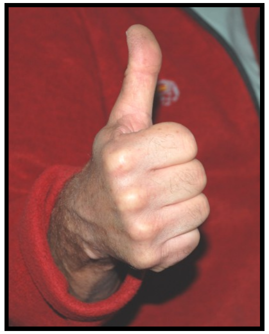

DO NOT PRINT
